# Cellular network modeling and single cell gene expression analysis reveals novel hepatic stellate cell phenotypes controlling liver regeneration dynamics

**DOI:** 10.1186/s12918-018-0605-7

**Published:** 2018-10-03

**Authors:** Daniel Cook, Sirisha Achanta, Jan B. Hoek, Babatunde A. Ogunnaike, Rajanikanth Vadigepalli

**Affiliations:** 1 Department of Chemical and Biomolecular Engineering, University of Delaware, Newark, DE USA; 2 Department of Pathology, Anatomy, and Cell Biology, Thomas Jefferson University, Philadelphia, PA USA

**Keywords:** Single cells, Hepatic stellate cell, Liver regeneration, Mathematical modeling, High-throughput data analysis

## Abstract

**Background:**

Recent results from single cell gene and protein regulation studies are starting to uncover the previously underappreciated fact that individual cells within a population exhibit high variability in the expression of mRNA and proteins (i.e., molecular variability). By combining cellular network modeling, and high-throughput gene expression measurements in single cells, we seek to reconcile the high molecular variability in single cells with the relatively low variability in tissue-scale gene and protein expression and the highly coordinated functional responses of tissues to physiological challenges. In this study, we focus on relating the dynamic changes in distributions of hepatic stellate cell (HSC) functional phenotypes to the tightly regulated physiological response of liver regeneration.

**Results:**

We develop a mathematical model describing contributions of HSC functional phenotype populations to liver regeneration and test model predictions through isolation and transcriptional characterization of single HSCs. We identify and characterize four HSC transcriptional states contributing to liver regeneration, two of which are described for the first time in this work. We show that HSC state populations change in vivo in response to acute challenges (in this case, 70% partial hepatectomy) and chronic challenges (chronic ethanol consumption). Our results indicate that HSCs influence the dynamics of liver regeneration through steady-state tissue preconditioning prior to an acute insult and through dynamic control of cell state balances. Furthermore, our modeling approach provides a framework to understand how balances among cell states influence tissue dynamics.

**Conclusions:**

Taken together, our combined modeling and experimental studies reveal novel HSC transcriptional states and indicate that baseline differences in HSC phenotypes as well as a dynamic balance of transitions between these phenotypes control liver regeneration responses.

**Electronic supplementary material:**

The online version of this article (10.1186/s12918-018-0605-7) contains supplementary material, which is available to authorized users.

## Background

Recent technological advances have enabled the study of transcriptional and proteomic profiles of single cells within a tissue at an unprecedented scale. Many cell types from diverse organs, developmental stages, and disease contexts have been profiled, revealing a high degree of variability in the expression of mRNA and proteins among single cells within a population (for examples, see [1–6]). Analysis of the variability in mRNA and protein expression at the single-cell scale (hereafter referred to as molecular variability) has revealed that the coordinated expression of genes within single cells allows cells to be organized into multiple sub-phenotypes, with different sub-phenotypes likely arising in response to different cellular inputs [7, 8], spatial location in the tissue [9, 10], developmental stage [11], and other intrinsic and extrinsic factors. The emerging view is that during development, and during homeostatic function of terminally differentiated tissues, cellular heterogeneity and the distribution of functional phenotypes are shaped by unique cellular inputs within an interacting network of cells constituting a tissue [12]. This cellular network reciprocally interacts with physiological features important to tissue function (e.g., blood flow, extracellular matrix stiffness, and oxygen content), as well as the molecular cues within the tissue microenvironment (such as cytokine, paracrine, calcium, or electrical signals), to shape tissue and cellular behavior. In other words, the behavior of each cell arises from its unique spatial and cell signaling “neighborhood”, and the interactions of heterogeneous cellular subtypes within these neighborhoods lead to an integrated tissue function. It has been proposed that the balance among heterogeneous cellular subtypes, i.e., the relative proportions of cells in each functional/phenotypic state, enables effective tissue-scale responses to perturbations in a manner that is not possible in tissues lacking heterogeneous cellular subtypes [13].

Despite significant research efforts, how the extensive variability intrinsic to molecular states of single cells translates to a tightly constrained tissue response to perturbations remains an open question. For instance, a prevailing view in neuroscience is that individual cellular variability is averaged out in cell populations, producing an electrical activity net rate code that governs circuit function [14]. Such a view of integrated cellular behavior is supported by studies of other cell types, which show, for instance, that while NF-κB levels oscillate over a wide range in single cells in response to stimulation, at the population level the response is more homogenous [15]. In contrast to this perspective, emerging results point to the need to consider explicitly the cellular subtypes to account for the overall tissue function [13, 16]. For instance, a recent study has shown that the variability observed in measurements of gene expression in single neurons is not entirely random, but arises from a distribution of cellular functional states ordered along a gene expression gradient between canonical molecular profiles corresponding to archetypal cell subtypes [7]. We address here the challenge of understanding the tissue scale physiological impact of such a single cell heterogeneity arising from multiple underlying cellular functional states. To this end, we collected and analyzed single cell gene expression data sets for distributions of functionally relevant phenotypes, and evaluated the impact of shifts in these distributions on tissue function, using a computational model of the cellular networks underlying overall tissue response. We use the process of liver regeneration to illustrate our approach.

The liver has a unique capacity to regenerate following up to 70–80% resection of total liver mass. This regenerative ability is crucial to functional recovery following surgical resection in treating liver disease (hepatocellular carcinoma, metastatic cancer, etc. [17, 18]), and after a live donor transplant [19]. While recovery from resection is due primarily to hepatocytes reentering the cell cycle to restore functional capacity of the liver, a coordinated response of a network of liver parenchymal and non-parenchymal cells is required to initiate, maintain and terminate the regeneration process effectively [20–22]. Such an organ-scale, well-controlled response to injury provides an excellent system for studying the problem of how the heterogeneous response of individual cells is integrated to yield a coordinated tissue-scale response after a physiological perturbation.

As a part of the liver cell network, non-parenchymal cells are crucial to controlling the dynamics of liver regeneration [23, 24]. Depleting Kupffer cells from the liver alters regeneration dynamics, with different studies reporting opposite effects of delaying regeneration [25] versus enhancing regeneration [26]. These opposite effects may reflect differences in the balance of functional states of Kupffer cells. Delayed regeneration occurred following depletion of both M1- and M2-polarized Kupffer cells, while targeted depletion of the M2-polarized Kupffer cells alone led to enhanced regeneration. Similar to Kupffer cells, endothelial cells have been shown to influence regeneration dynamics, although they do so through the production of hepatocyte growth factor (HGF) [27], Wnt2 [28], angiopoietin 2 [29], and likely other factors as well.

Our own work using mathematical modeling to understand progression of liver regeneration has identified hepatic stellate cells (HSCs) as important controllers of regeneration dynamics [23, 30]. In parallel, our experimental studies suggest that the dysregulation of HSC functional phenotypes is a mediator of the chronic ethanol-induced suppression of regenerative response to injury [31, 32]. While there is some recognition of HSCs as multifunctional cells contributing to liver homeostasis, repair, and disease etiology, the molecular and cellular dynamics of HSCs have been studied primarily in the context of liver fibrosis, emphasizing a canonical view of HSCs as either quiescent (storing retinol) or activated (producing extracellular matrix constituents) [33]. Recent work challenges this two-state view by defining a new HSC state, termed the “inactive state”, which is molecularly distinct from the quiescent state and whose response to activation signals is different from that of quiescent HSCs [34]. In the context of regeneration, several questions about HSC behavior remain: What are the identities of HSC functional states during liver regeneration? How do these HSC functional states and state transitions contribute to the dynamics of liver functional mass recovery? How is the balance of functional HSC states connected to defective overall tissue regenerative responses observed in disease or dysfunctional cases? One challenge in tackling these questions is in interpreting the “snapshot” single cell transcriptional data for insight into the dynamics of tissue function.

To address these and related questions, we employed an approach that integrates computational modeling and in vivo experiments. The results described below are organized as follows:

- First, we describe a newly developed multi-scale cellular and molecular network-based computational model of liver regeneration that incorporates multiple functional states of cells and dynamic interactions between hepatocytes and liver non-parenchymal cells.
- Next, we describe a set of model simulations that have led us to a prediction that balances of cellular functional states aid in the control of liver regeneration dynamics.
- Subsequent sections describe the results from experimental testing of these predictions using transcriptional profiling of single cells to uncover the identities and distributions of HSC functional states in response to liver resection, under alcoholic fatty liver disease and matched control conditions.
- Finally, we describe the use of our computational model-based simulations as a framework to interpret the single cell experimental results and evaluate the impact of shifts in HSC functional state imbalances on the dynamics of liver regeneration.

The present study significantly expands the previous computational models to include dynamics of state transitions in Kupffer cells and HSCs. In addition, the present manuscript describes novel results from high-throughput experiments on single cell gene expression in HSCs during liver regeneration, which identified HSC functional states that have not been previously described. The single cell scale transcriptional assays employed in this study enable identifying and characterizing HSC functional states based on tens to hundreds of simultaneous measurements in individual cells and pools of cells. Hence, the present manuscript comprises distinct and novel computational and experimental results that significantly extends the previous hepatocyte-focused computational model of liver regeneration towards a multicellular tissue context. Taken together, our approach provides a framework for integrating single cell data sets and information on cellular-scale functional state transitions with tissue-scale dynamics. This framework can be applied to multiple organs, physiological challenges, and diseases towards a more comprehensive understanding of the contributions of cell state balances to dynamic tissue function.

## Results

### A new multiscale cellular and molecular network model of liver regeneration

**Fig. 1:**
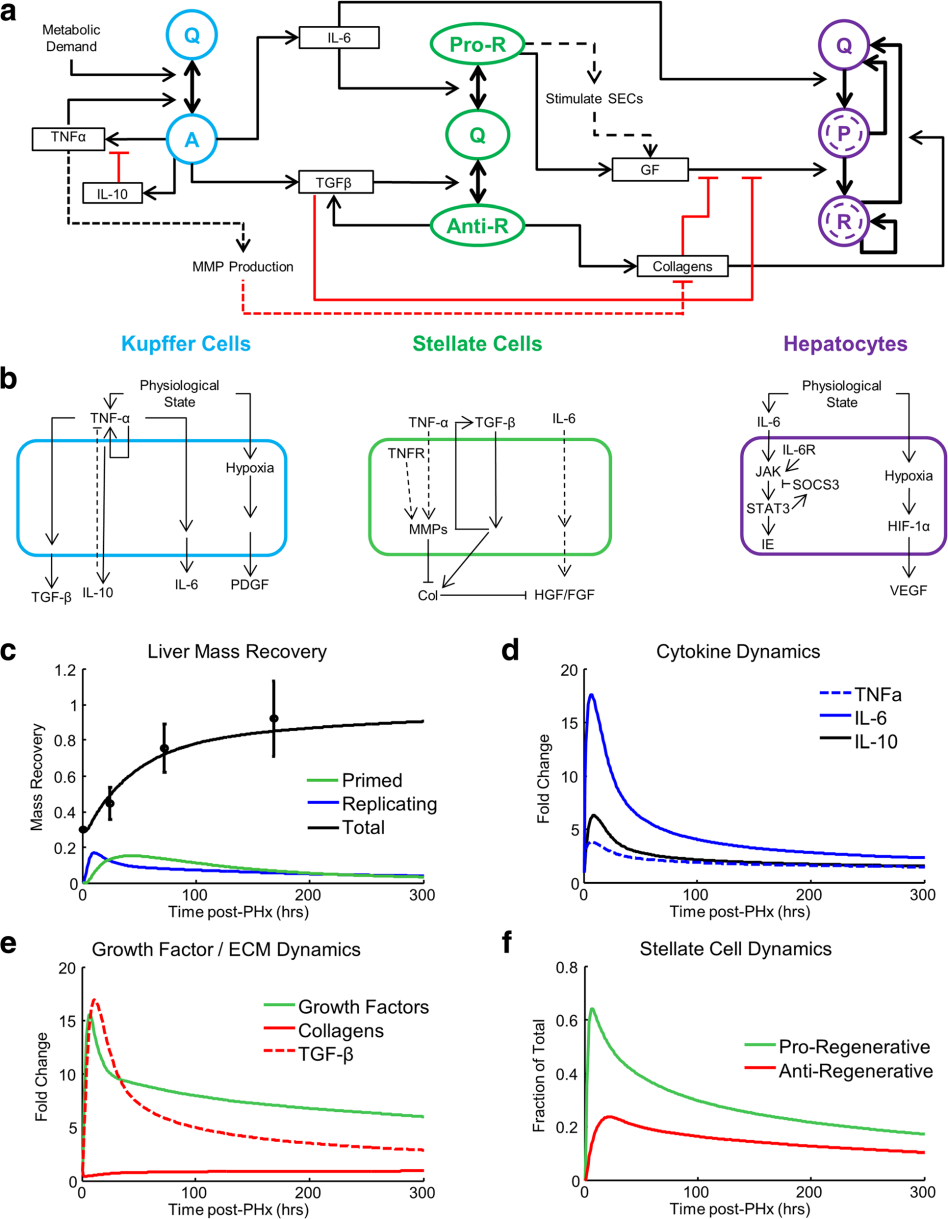
Cell network model of non-parenchymal cell activation contributing to liver regeneration. **a** Complete network diagram showing cell states, transitions among states, and molecules promoting or inhibiting cell transitions. Kupffer cell states are distributed between Quiescent (Q) and Active (**a**) states. Stellate cell states are distributed between Quiescent (Q), Pro-regenerative (Pro-R), and Anti-regenerative (Anti-R) states. Hepatocyte states are distributed between Quiescent (Q), Primed (P), and Replicating (R) states. **b** Cell signaling models showing a schematic representation of molecular interactions occurring within each cell type considered. Solid lines represent directed signaling; dashed lines represent indirect effects. **c** Simulated liver mass recovery profiles compared to the experimental data from [36]. **d** Simulated cytokine dynamics following resection. **e** Simulated dynamics of growth factors and collagens following resection. **f** Simulated profiles of HSC state dynamics following resection. Definition of additional terms: TNFα = tumor necrosis factor α, IL-10 = interleukin 10, IL-6 = interleukin 6, TGFβ = transforming growth factor β, MMP = matrix metalloproteases, GF = growth factors, PDGF = platelet derived growth factor, TNFR = TNF receptor, HGF = hepatocyte growth factor, FGF = fibroblast growth factor, IL-6R = IL-6 receptor, JAK = Janus kinase, STAT3 = signal transducer and activator of transcription 3, SOCS3 = suppressor of cytokine signaling 3, IE = immediate early genes, HIF-1α = hypoxia inducible factor 1α, VEGF = vascular endothelial growth factor, and SEC = sinusoidal endothelial cells

We developed a computational model of liver regeneration that includes both hepatocyte hypertrophy and hyperplasia (Fig. 1a and b). Briefly, each cell type (hepatocytes, Kupffer cells, and HSCs) is considered as distributed across discrete functional states, with cells in each state secreting distinct factors that lead to distinct cell functional state transitions. The cells also respond to physiological perturbations by transitioning among states, increasing mass, or undergoing cell death. The most notable feature of the model, which distinguishes the network scheme from previous models of liver regeneration, is the consideration that HSCs are distributed across three discrete states, termed ‘quiescent’, ‘pro-regenerative’, and ‘anti-regenerative’ for their putative impact on the tissue regeneration process. See the Materials and Methods section for a detailed rationale for model development and description of equations. Model parameter definitions and their nominal values are provided in Additional file 1: Table S1. Model parameters for hepatocytes are adapted from [23, 35]; model parameters for other cell types are chosen to have similar magnitudes as hepatocyte parameters and are optimized to fit experimental data (Fig. 1c). A concern is that large number of parameters and the relatively fewer number of experimental observations, however, makes model parameters largely unconstrained by experimental values. We utilize global sensitivity analysis to at least partly address this issue.

### Dynamic balance of hepatic stellate cell states during regeneration

Our computational model prediction of the dynamics of liver mass recovery following resection in rats match corresponding experimental observations (Fig. 1c). Primed hepatocyte levels peak early post-partial hepatectomy (PHx), within the first 12 h, and remain at lower levels as the regeneration progresses. This early priming peak is consistent with experimental data showing an approximately 16-h delay between resection and the onset of DNA replication in rats [37]. In model simulations, levels of replicating hepatocytes peak at ~ 26 h post-PHx. This result is consistent with the results of experimental studies showing that BrdU incorporation peaks at 24 h post-PHx [37]. Our simulation results are also consistent with experimental observations of the tissue microenvironment following PHx. Our simulations show TNF-α and IL-6 increase early post-PHx, peaking at ~ 6 h post-PHx (Fig. 1d), closely matching experimental data showing TNF-α and IL-6 levels peak in the serum at 12 h post-PHx in rats [38]. In addition, our simulation results show that IL-10 increasing early post-PHx but with peak levels delayed until ~ 9 h post-PHx, matching experimental observations that IL-10 increases during the priming phase after TNF-α and IL-6 increase, and that IL-10 has a lower peak level than IL-6 (Fig. 1d) [38]. Our simulation results match experimentally reported dynamic changes in growth factor levels (GF) (similar to rat hepatocyte growth factor or HGF dynamics) and TGF-β (similar to mouse TGF-β dynamics) levels, with both factors increasing early post-PHx and decaying thereafter (Fig. 1e) [39, 40]. The GF profile peaks prior to that of TGF-β and remains elevated for a longer duration, facilitating hepatocyte entry into the cell cycle. ECM degrades rapidly post-PHx, but returns to baseline levels by the termination of regeneration, in agreement with results from experimental studies of matrix dynamics during liver regeneration (Fig. 1e) [41]. While the model includes a single GF, the biological correlate of the model GF variable could include several growth factors, with potential slight differences in their dynamics. Note that our simulations indicate the existence of an early transition of quiescent HSCs into both pro-regenerative and anti-regenerative states (Fig. 1f). However, the proportion of pro-regenerative HSCs peaks before that of anti-regenerative HSCs, and the balance of cell states favors the pro-regenerative state during the early stages of regeneration (0–100 h post-PHx). After levels peak, the fraction of pro-regenerative HSCs decreases at a faster rate than that of the anti-regenerative HSCs. After ~ 100 h post-PHx, the ratio of pro-regenerative and anti-regenerative HSCs tends to equalize, which may contribute to the slowing and eventual termination of regeneration (Fig. 1c).

### Hepatocyte apoptosis rate bifurcates liver recovery and failure

**Fig. 2:**
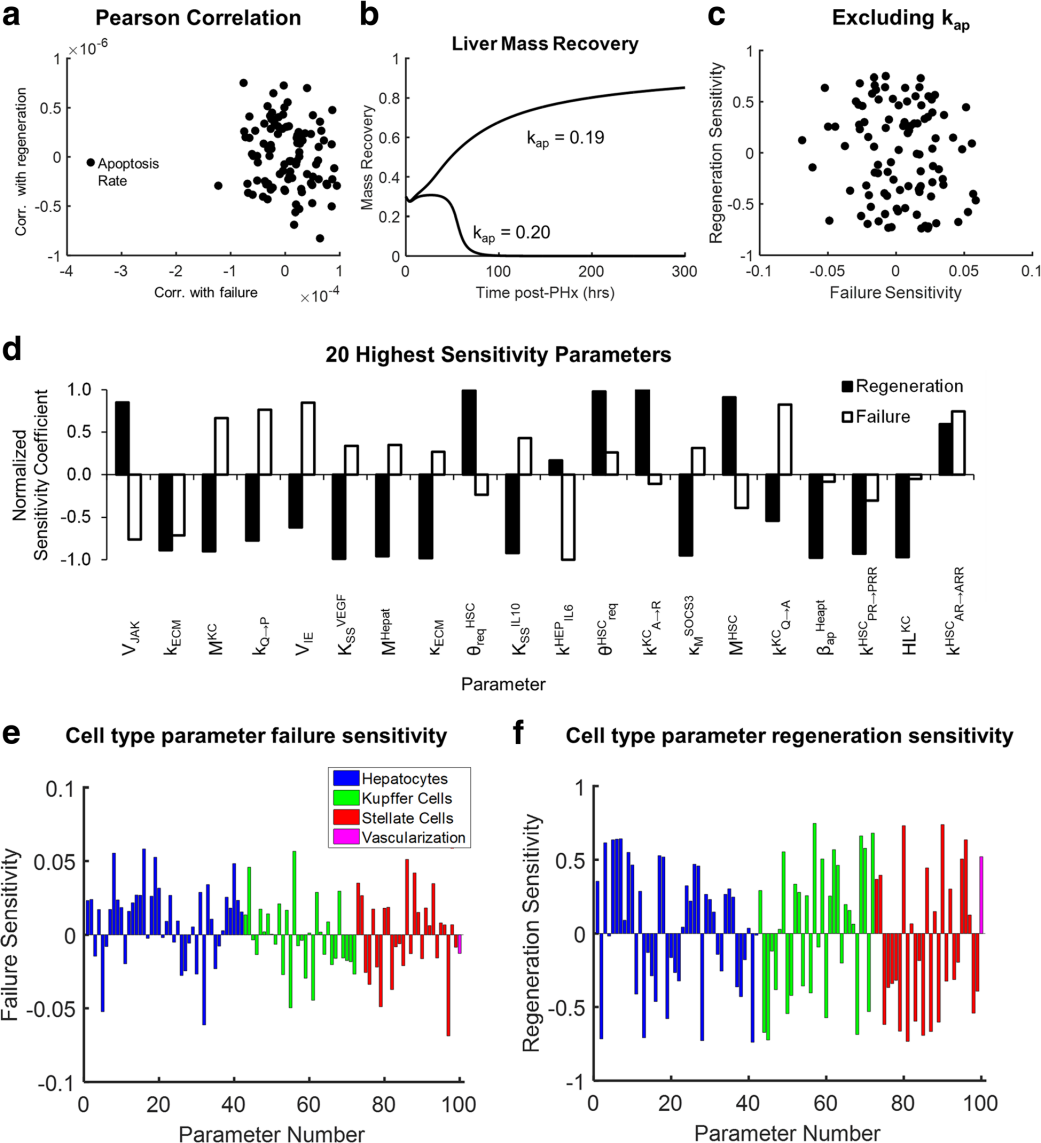
Parameters from all cell types contribute to regeneration dynamics and time to failure. **a** Changing hepatocyte apoptosis rate results in a bifurcation between liver recovery following resection and liver failure. **b** Removing hepatocyte apoptosis rate from the global sensitivity analysis shows that regeneration and time to liver failure are sensitive to changes in many parameters. **c** The 20 parameters with the highest combined sensitivity coefficients for regeneration amount and time to liver failure. Of the top 20 most sensitive parameters, 8 are HSC-related parameters, 7 are hepatocyte-related parameters, and 5 are KC-related parameters. **d** Time to failure sensitivity coefficients for parameter values associated with each cell type. 67% of hepatocyte parameters show positive failure sensitivity (compared to 48% for KCs and 50% for HSCs). **e** Regeneration sensitivity coefficients for parameter values associated with each cell type. 60% of HSC parameters show negative regeneration sensitivity (compared to 45% for hepatocytes and 43% for KCs)

We performed a global sensitivity analysis to identify which parameters are important regulators of liver regeneration dynamics. We selected values for each parameter from a uniform distribution with a 10-fold range around its nominal value (0.1× to 10×). In our initial simulations, we noticed that certain model simulations with parameter values far from nominal were taking a large amount of time, possibly due to model stiffness and numerical integration issues. We therefore performed two global sensitivity analyses. For the first global sensitivity analysis, we simulated the model following 70% PHx 150 times, using a new set of parameter values each time. We observe that liver mass profiles can be divided broadly into regenerating and non-regenerating responses, and that there are more cases of the latter than of the former. Regenerating responses result in a liver mass greater than the initial amount present post-PHx, and non-regenerating responses result in a liver mass that does not increase following resection (unresponsive) or that causes a complete loss of functional liver mass (liver failure). We find that a threshold value of the hepatocyte apoptosis rate (in this case, 2 times the nominal value) bifurcates the response between liver recovery and liver failure (Fig. 2a, Additional file 1: Figure S1). We tested all other model parameters for bifurcation potential and find that several other parameters that are able to bifurcate the response between recovery and failure. These include parameters related to hepatocyte apoptosis – the hepatocyte apoptosis shape and scale parameters (θ_ap_ and β_ap_) (Additional file 1: Figure S1) – as well as the parameters JAK degradation rate (κ_JAK_), SOCS3 activation rate (V_SOCS_), *K*_*m*_ for SOCS3, IE gene degradation rate (κ_IE_), and cell growth rate (k_growth_) and the Kupffer cell parameters TNFa degradation (κ_TNF_) and IL-6 degradation (κ_IL6_). The relative changes that must be made to these parameter values, however, are greater than need to be made to hepatocyte apoptosis rate to bifurcate recovery and failure response.

### Coordinated response of multiple cell types is required for effective regeneration

Next, we performed a second global sensitivity analysis on the influence of parameter values on liver mass recovery using the partial rank correlation coefficient (PRCC) method [42]. We simulated regeneration using 1500 parameter sets varying all parameters except the hepatocyte apoptosis rate, since we have found that varying apoptosis rate has a disproportionate effect on our analysis (and simulation run times). The global sensitivity analysis results enable us to identify key parametric contributors to regeneration or liver failure (Fig. 2b). We identified the top 20 parameters contributing to liver regeneration or failure by calculating a sum of squares distance metric for each parameter to understand which parameters affect regeneration dynamics most strongly (Fig. 2c). Parameters that contribute positively to regeneration, tend (mostly, but not always) to contribute negatively to liver failure. Although not in the top 20 highest sensitivity parameters, monomeric STAT-3 concentration shows a positive contribution to both regeneration and liver failure. This finding is consistent with previous experimental studies showing that acute inhibition of STAT-3 and long-term inflammation involving STAT-3 activation both suppress regeneration [43, 44].

We examined cell-type specific parameters to identify which cell types contribute the most to liver failure and regeneration. We find that the liver failure response tends to have the highest sensitivity coefficients associated with hepatocyte and HSC parameters, with 8 of the top 20 parameters associated with hepatocytes, and 8 of 20 associated with HSCs. One way to interpret parameter changes biologically is to view parameter changes as changes to cell behavior that occur as preconditioning prior to resection. Therefore, the finding that hepatocyte and HSC parameters affect regeneration dynamics strongly suggests that hepatocyte and HSC preconditioning prior to resection plays a significant role in determining whether a liver will regenerate or fail (Fig. 2d). Among the most sensitive parameters affecting time to liver failure are steady-state production of Tgfb by HSCs (K_SS_^Tgfb^), concentration of monomeric STAT3 ([STAT3]), IE gene activation rate (V_IE_), and quiescent-to-primed transition rate (k_Q → P_). In addition, we find that no cell type had parameters with disproportionally large sensitivities (i.e., no cell type was associated with parameters showing disproportionately large effects on regeneration dynamics), indicating that regeneration requires a coordinated regulation of all cell types in the liver (Fig. 2e). Among the most sensitive parameters affecting liver regeneration are the hepatocyte parameters K_m_^JAK^, K_m_^SOCS3^, and β_ap_; the Kupffer cell parameters k^KC^_Q-A_ and hypoxia load (HL^KC^); the HSC parameters θ^HSC^_req_, k_ECM_; κ_ECM_, and κ_deg_; and the physiological parameters representing metabolic demand (M) for all three cell types.

### Timing and balance of hepatic stellate cell functional states are critical controllers of liver regeneration dynamics

**Fig. 3:**
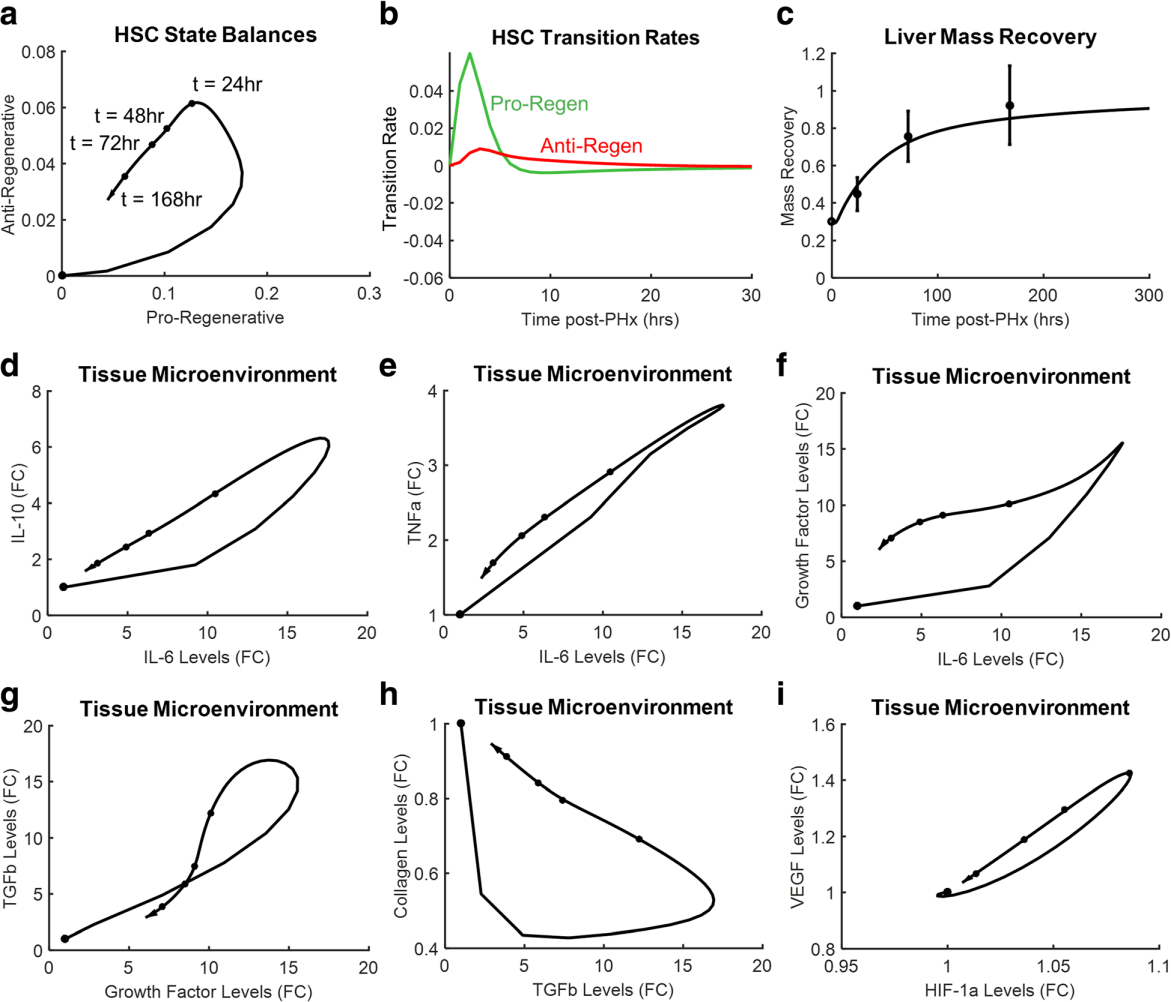
The dynamics of cell state balances and tissue microenvironment factors during successful liver regeneration visualized using a phase plane analysis. **a** Levels of pro-regenerative and anti-regenerative HSCs. **b** Transition rates of HSCs between quiescent and pro-regenerative (green) and between quiescent and anti-regenerative (red) states. **c** Simulated liver mass recovery compared to experimental data [36]. **d-i** Dynamic tissue microenvironment influences and is shaped by cell state balances. Dots on the phase planes represent time = 0 h, 24 h, 48 h, 72 h, and 168 h following resection

For the successful regeneration scenario, we investigated how the balance of HSC states changes in response to resection. We find that levels of pro-regenerative and anti-regenerative HSCs peak prior to or at 24 h post-PHx, and decrease thereafter (Fig. 3a). The phase-plane representation used in Fig. 3a allows for visualizing the coordinated behavior of the two cell states. The levels of cells in both states increase early post-PHx, with pro-regenerative levels peaking earlier than the peak of anti-regenerative levels. This coincides with an earlier and higher magnitude peak transition rate in pro-regenerative cells (Fig. 3b). The dynamic interplay between the HSC cell states contributes to a successful regeneration response that matches the experimentally observed mass recovery dynamics following PHx in rats (Fig. 3c).

### Dynamic tissue microenvironment cycles underlie regeneration dynamics

In the present computational model, the distribution of cell functional states affects the molecular state of the tissue microenvironment, which in turn regulates the transition between cell states. Figures 3d-i show the coordinated regulation pairs of associated factors representing the tissue microenvironment following resection. Each pair of factors shows a cyclic response to resection, with levels of these factors eventually returning to normal.

### Hepatic stellate cell isolation and identification reveals high transcriptional variability within a cell type

**Fig. 4:**
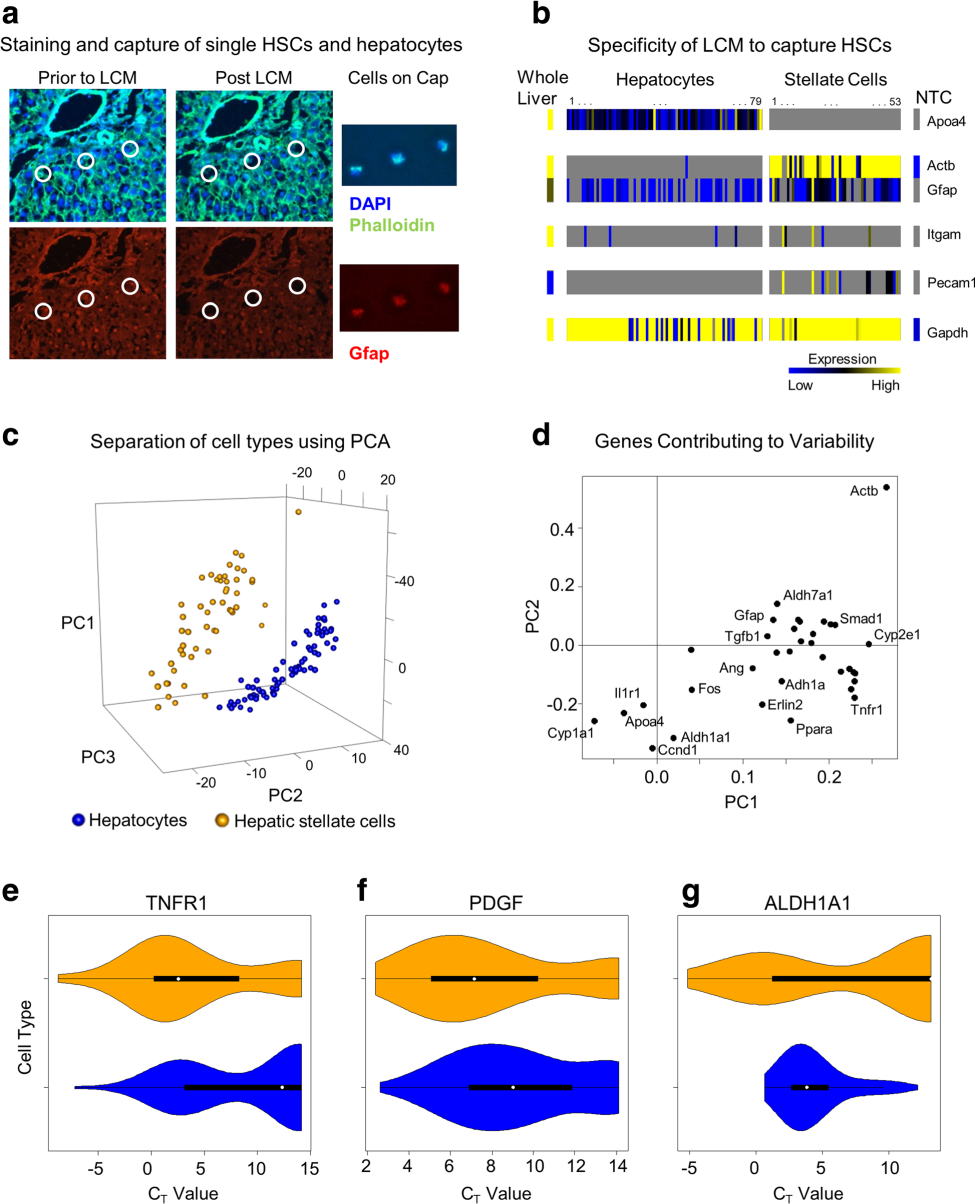
In situ isolation and high-throughput gene expression profiling of single/pooled hepatic stellate cells and hepatocytes. **a** Representative images showing LCM isolation of single hepatic stellate cells. DAPI and Phalloidin staining were used to identify nuclei and cell boundaries. Co-localization of DAPI and Gfap was used to identify HSCs. **b** Single HSCs and hepatocytes were collected from the same tissue and tested for cell type marker genes using high throughput qPCR. Cells express high levels of marker genes in a cell-type specific manner. **c** Using PCA on expression levels of 34 genes measure in both hepatocytes and HSCs separates cell types. The variability within a cell type appears to be higher than variability between cell types. **d** PCA scores shows the genes contributing to the PCA plot. **e** Different genes show unique patterns when comparing cell types. TNFR1 shows different mean CT values but similar variability. **f** PDGFA shows similar mean CT values and similar variability. **g** ALDH1A1 shows different mean CT values and different variability

Our simulations point to hepatic stellate cell state balances as important modulators of liver regeneration dynamics; however, the existence of multiple stellate cell states has never before been shown during liver regeneration. We therefore set out to identify signatures of HSC states through their transcriptional regulation by collecting and analyzing a high-dimensional dataset of gene expression from single HSCs. We obtained liver tissue before and after 70% PHx from chronic ethanol-fed and isocaloric carbohydrate-fed control rats. These experimental conditions allow us to examine the distribution of HSC states before and after PHx in the cases of normal regeneration (control group) and deficient regenerative response (ethanol group). We used laser capture microdissection (LCM) coupled with high-throughput qPCR (Biomark™) to isolate and transcriptionally characterize single HSCs (Fig. 4a). We chose the time point 24 h post-PHx, corresponding to the peak of hepatocyte replication in rats post-PHx [37]. To confirm cell-type specificity, we also isolated and transcriptionally characterized single hepatocytes from the same tissue. Using this high-throughput approach, we measured the expression levels of ~ 100 genes in each of ~ 140 single HSCs with high reproducibility (Additional file 1: Figure S2). LCM made possible the capture of single HSCs and hepatocytes with low levels of contamination (Fig. 4b) and a high degree of cell type specificity (Fig. 4c). Principal component analysis (PCA) was used to identify variability between cell types and to quantify major sources of variability within the gene expression data (Additional file 1: Figure S3). We find that, although there is clear separation between HSCs and hepatocytes, the variability within a cell type is of the same order of magnitude as the variability between cell types (Fig. 4c). This surprising finding suggests that gene expression varies within a cell type in a large range similar to the differences in expression levels between distinct cell types (hepatocytes and HSCs, in this case). Several of the major contributors to variability in gene expression within a cell type and between cell types are ethanol metabolism genes (Cyp1a1, Aldh1a1, Adh1a, Cyp2e1), genes associated with HSC activation (Actb, Smad1, Ppara), and growth factors (Ang, Pdgfa, TGFb1) (Fig. 4d). Moreover, the distribution of gene expression within a cell type is different between HSCs and hepatocytes. Some genes are expressed at different levels with similar distribution ranges (Fig. 4e), while other genes are expressed at similar values and ranges (Fig. 4f), or at different levels with different distribution ranges (Fig. 4g).

### Single-cell based transcriptional analysis reveals novel HSC populations

**Fig. 5:**
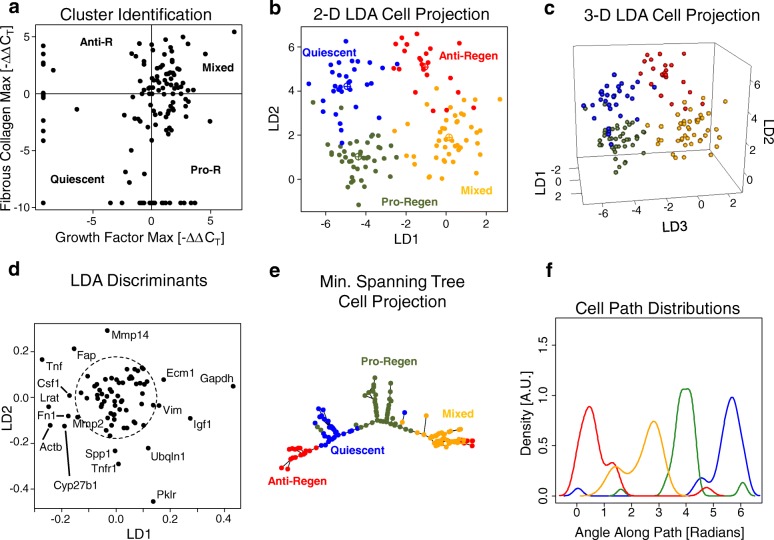
Gene expression in single hepatic stellate cells reveals functional transcriptional states. **a** Manual clustering of functional HSC states reveals four states: Quiescent (low GF/low collagen), Pro-regenerative (high GF/low collagen), Anti-regenerative (low GF/high collagen), and mixed (high GF/high collagen). **b** Linear discriminant analysis shows separation of the four HSC states in two dimensions. **c** LDA shows further separation of the four HSC states in three dimensions. **d** Genes contributing to discrimination among functional states. **e** Minimum spanning tree representation of single HSCs shows correlative relationships between individual cells and putative trajectories of transition across HSC states. **f** Cyclic representation of HSC functional states shows the potential distribution of individual HSCs as they progress through the cycle

We used a guided clustering technique to identify transcriptional states of HSCs across all conditions: ethanol-fed and control rats prior to and post-PHx. First, we categorized each cell based on the expression of fibrous collagen-related genes (Col3a1, Col14a1, and Ecm1) and growth factors (Hgf, Igf1, and Vegf) (Fig. 5a). This allowed us to organize cells into four categories: GF high, collagen low (pro-regenerative); GF low, collagen high (anti-regenerative); GF high, collagen high (mixed); and GF low, collagen low (quiescent). Next, we calculated Pearson correlations of the expression of all genes in each sample to the centroid of gene expression of each sample category and reclassified samples with sufficiently high correlations (*p*-value ≤ 0.05) into the category to which they most closely correlated. We then used linear discriminant analysis to visualize the gene expression state of each single cell and how the cells were organized into distinct states based on the expression level of all genes measured (Fig. 5b and c). In addition to the genes used for classification, we identified several discriminant genes that may be useful as biomarkers to differentiate among these states, including Actb, Mmp14, Mmp2, Igf1, Tnfr1 and Tgfbr2 (Fig. 5d, Additional file 1: Figure S4).

Next, to ensure that the separation we observed among HSC states was not an artifact of our data analysis technique, we randomized our data 1000 times, re-performing our analysis on the randomized data, and calculating a silhouette score for each randomization. The silhouette score is a measure of the “between-ness” of clusters normalized by a commensurate measure of the “within-ness” of clusters; therefore, a high silhouette score indicates separation among tightly packed clusters. The silhouette score of the clusters from the original single cell gene expression data fell outside the distribution calculated from randomized data at an empirical *p*-value less than 0.001 (Additional file 1: Figure S5). The silhouette score calculated from our experimental data and associated with this low *p*-value indicates that the cell states do not overlap significantly when projected onto the linear discriminant axes compared to randomized data, suggesting that the identified HSC states are governed by distinct transcriptional regulation. We further validated these results using gene expression profiles from thirty-six 10-cell pools of HSCs (Additional file 1: Figure S6). Our results from 10 cell pools are similar to our results from single cells but not identical (most notably in the minimum spanning trees in Fig. 5e and Additional file 1: Figure S6E). The differences between our single cell results and the pooled cell results likely arise because the pooled cell data includes a mixture from multiple cell states. Therefore, in this case, we use the single cell data to understand potential transitions across states. We sought to understand how an individual cell may transition across states by using a minimum spanning tree projection (Fig. 5e). The minimum spanning tree projection connects each cell with its nearest neighbors according to an algorithm that minimizes the total connectivity of the network and prevents connection cycles. This type of projection gives an idea of how individual cells within a population could transition between states to minimize the transcriptional changes required to move from one state to another but is not a formal method to identify dynamic progression from “snapshot” data [45]. The minimum spanning tree projection suggests a dynamic shift in HSC transcriptional regulation whereby HSCs transition from a quiescent phenotype to either a pro-regenerative phenotype or an anti-regenerative phenotype and that pro-regenerative cells can transition to a mixed state then to an anti-regenerative state. It is also possible that, in contrast to a linear progression through states, HSC transitions occur in a cycle analogous to the cell cycle. If such a cycle is the case, the balance of HSCs may be represented as a distribution of states within a cycle (Fig. 5f). Further experimental work, including cell tracing studies, is needed to distinguish between these putative models of HSC state transitions.

**Fig. 6:**
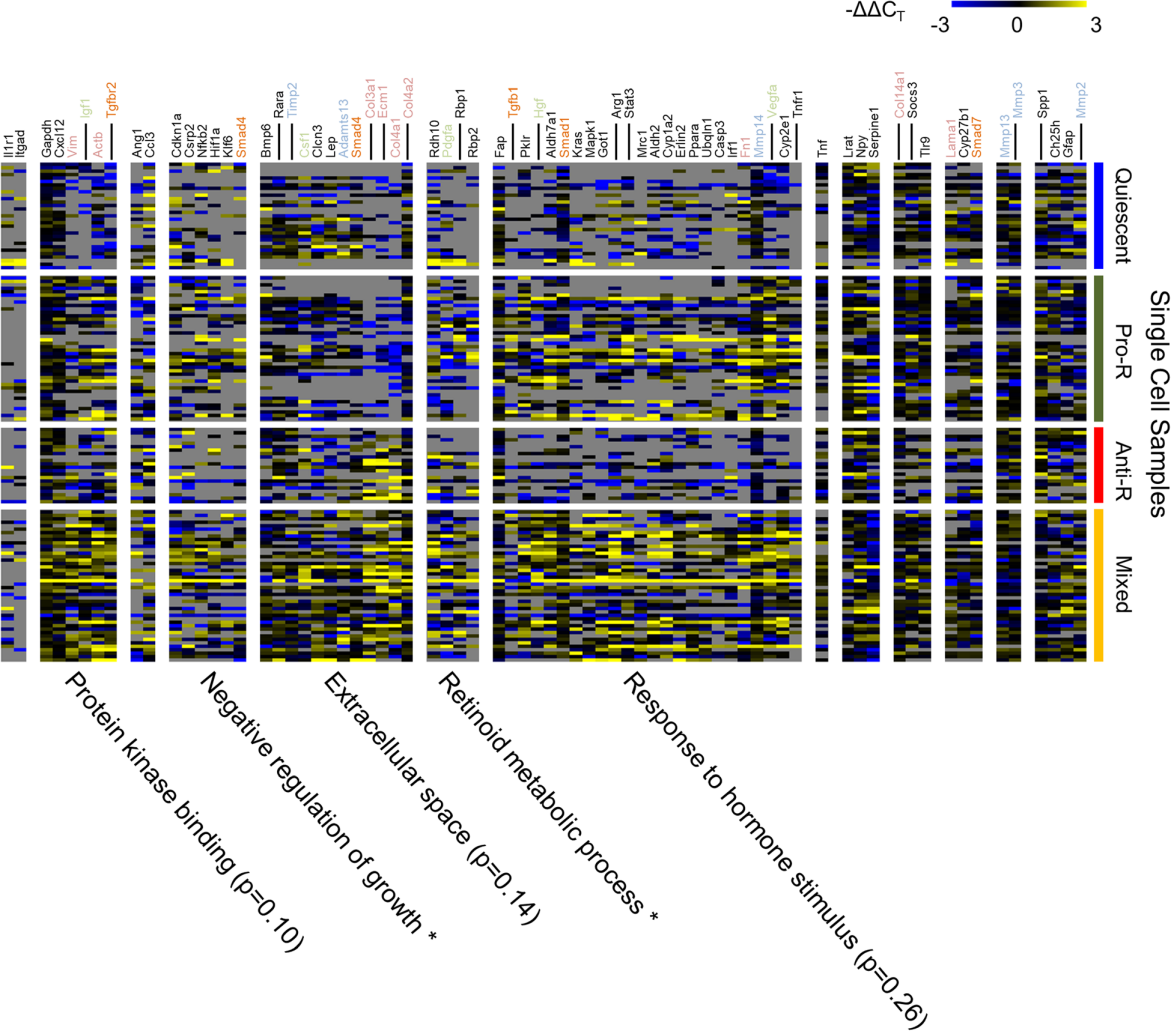
Heatmap representation of gene expression values for individual HSCs. Cells are grouped based on functional state. Genes are grouped by hierarchical clustering based on Pearson correlation. Gene annotations are colored based on functional annotations. Genes discussed in the text have their names offset for ease of identifying them. Green = growth factors, gray = collagen-related genes, orange = Tgfb signaling, blue = matrix-modulating genes. We also show overrepresented functions (GO Terms) for each gene group identified in our single cell data compared to the background of all genes measured. * Indicates *p*-value < 0.05

We next sought to identify the modules of gene expression that govern each cellular state (Fig. 6). We grouped the genes using hierarchical clustering based on Spearman rank correlation (Additional file 1: Figure S7). We then colored the genes according to their annotated and previously reported functions (Growth factors – light green, Tgfb signaling – light orange, collagens & matrix-related genes – light red, and matrix remodeling – light blue). In addition, we used the online DAVID software for pathway analysis to identify the functional annotations enriched within each group compared to all the genes we measured, as an unbiased approach to investigate the putative functions of the gene modules (Fig. 6) [46]. We then grouped cells within each state by hierarchical clustering using Spearman rank correlation. We find that distinct HSC states tend to express distinct functional modules of genes. Quiescent HSCs tend to express high levels of the basement membrane gene Col4a2 and the matrix metalloprotease responsible for regulating Collagen-4 (Mmp2) and its regulator (Timp2), which has been shown to work with Mmp2 to degrade collagens. This suggests that quiescent HSCs play a more dynamic role in maintaining basement membrane architecture than previously suspected in addition to their canonical role of storing vitamin A. Pro-regenerative HSCs express genes aiding in regeneration, including the growth factors Igf1, Arg1, Vegfa, and Hgf. Cells within these clusters also expressed other genes, including Stat3, Socs3, and Tnfr1, suggesting that interleukin signaling could be the primary driver of HSC transition from the quiescent state to the pro-regenerative state. Anti-regenerative HSCs express high levels of Col3a1, Col14a1, and Ecm1. Several of these cells also show high expression of Mmp2, and Mmp3, suggesting that cells in the anti-regenerative state remodel existing matrix and deposit fibrous matrix. The anti-regenerative HSCs also tend to express higher levels of Spp1, which has been shown to activate HSCs toward a pro-fibrotic state, suggesting a positive feedback from the anti-regenerative state to recruit more cells into this phenotype. Cells in the anti-regenerative state also tend to express higher levels of the TGF-β receptors Tgfbr2 (as did pro-regenerative and mixed stellate cells), but not the ligand Tgfb1, which is expressed at highest level in pro-regenerative and mixed state HSCs. This indicates that HSCs in the anti-regenerative state may be sensitive to TGF-β signaling, but may not be the key source of TGF-β in the tissue. The mixed phenotype expresses high levels of both the collagen deposition module and growth factor production modules. What separates this state from the anti-regenerative state is the high expression of genes related to TGF-β signaling, cytokine signaling, and retinol metabolism, including Rara and Rbp1. Several cells in the mixed and pro-regenerative states express Tgfb1; therefore, there may be a coordination of anti-regenerative HSCs and either mixed or pro-regenerative HSCs required for sustaining anti-regenerative HSC populations during chronic liver disease.

Understanding the molecular regulation underlying each state suggests additional annotations that may be appropriate to label the cell states. HSCs with high MMPs may be classified as quiescent or as matrix-modulating. HSCs with high GFs may be classified as pro-regenerative in the context of response to PHx or as cytokine-regulated HSCs or GF depositing HSCs in the context of normal tissue function. HSCs with high levels of collagens may be classified as anti-regenerative in the context of response to PHx, as TGF-β response primed HSCs in the context of normal tissue function, or as pre-fibrotic in the context of disease progression. HSCs that express high levels of GFs and collagens may be classified as a mixed phenotype in the context of regeneration or as a hyper-functional HSC or adaptive HSC in the context of normal liver function. These adaptive HSCs may express high levels of multiple functional gene modules to be able to respond to external stimuli efficiently without having to produce additional transcripts.

### Dynamic balance of HSC states post-PHx is altered between health and disease

We next investigated how the balance of HSC states progresses during liver mass recovery (Table 1). We examined the distributions of cells within the four identified cell states in four different ways. We first investigated whether it was likely that at least one of the states resulted in a distribution of cells different than the others. We then examined how cells are distributed in healthy livers prior to and post-PHx. Subsequently, we compared the distribution of cells among states between healthy livers and alcohol-adapted livers prior to PHx. Finally, we compared the distribution of cells among states between healthy liver and alcohol-adapted livers at 24 h post-PHx. Cells from three biological replicates were collected and the distributions of these cells among states are reported in Table 1.

**Table 1:** Distribution of hepatic stellate cell states in each condition

State	Control 0 h	Ethanol 0 h	Control 24 h	Ethanol 24 h
Quiescent	0.11	0.31	0.21	0.29
Pro-Regen	0.29	0.17	0.38	0.39
Anti-Regen	0.21	0.33	0.06	0.00
Mixed	0.39	0.19	0.35	0.32
Total Cell Number	38	36	34	31

We used Pearson’s Chi-squared test to evaluate whether the distribution of cells among states was independent of the condition (control, ethanol-adapted, prior to PHx, and 24 h post-PHx). We found that the cell state distribution is not independent of experimental condition (*p*-value = 0.002); although it should be noted that this test may give incorrect results for sample sizes as small as in our study. Nevertheless, this result indicates that at least one condition likely results in a distribution different from the others.

We examined how the distribution of cells changes prior to and following PHx. We expected a high proportion of HSCs in the livers of control animals prior to resection to be in the quiescent state; however, we find that HSCs are distributed among all the possible states with a small fraction of cells in the quiescent state (11% of total cells). Control livers at baseline have a high proportion of cells in the mixed state (39%) and in the pro-regenerative state (29%), and a slightly lower proportion of cells in the anti-regenerative state (21%). We used Fisher’s exact test for count data to compare the distribution of cells in healthy rat livers prior to and post-PHx. We found no statistical evidence to support a difference in distribution due to PHx (Benjamini & Hochberg corrected *p*-value = 0.37). The following differences we observe may therefore be an artifact of our small sample size. Following resection, control livers show a strong response of pro-regenerative cells (38%) and maintain a large population of mixed cells (35%). Relatively few cells are found in the anti-regenerative state (6%). The balance of cells in the pro-regenerative and mixed states appears to shift at 24 h post-PHx compared to baseline, i.e., the relative proportion of pro-regenerative cells to mixed cells is higher at 24 h post-PHx compared to the baseline ratio. Following resection, it is possible that cells in the mixed state shift to the pro-regenerative state. A larger sample size of single cells, however, is necessary to robustly determine a shift in the cell state distribution. Such a shift may correspond to lifting a “brake” on growth factor effectiveness, allowing the hepatocytes to progress through the cell cycle.

#### HSC state dynamics during ethanol-preconditioning and impaired regenerative response to partial hepatectomy

Following adaptation to chronic ethanol consumption, the regenerative ability of the liver is greatly reduced, leading to suppression of hepatocyte replicative capacity that is apparent through reduced tissue mass by 24 h post-PHx and exaggerated by 48 h post-PHx [31, 47]. We investigated how chronic ethanol feeding alters the balance of HSC states at baseline and at 24 h post-PHx (Table 1). We found that largest difference in HSC state balances in ethanol-fed rat livers prior to resection. In contrast to control livers, ethanol-fed rat livers have a high proportion of cells in the anti-regenerative state (33%) prior to resection (baseline). This condition also shows a reduced fraction of cells in the pro-regenerative (17%) and mixed states (19%). Fisher’s exact test for count data indicates that cell distributions are dependent on ethanol adaptation when comparing healthy livers to ethanol adapted livers prior to PHx (Benjamini & Hochberg corrected *p*-value = 0.051). This result suggests that the main effect of chronic ethanol intake on HSCs is to enhance HSC transition to the anti-regenerative state and attenuates other HSC states prior to resection. Such a cell state population balance after adaptation to chronic ethanol intake may result in a preconditioning of the extracellular matrix in such a way as to impair regeneration following resection.

Our model predictions, however, are not entirely consistent with our experimental results (shown in Table 1) on the distribution of HSC states post-PHx in ethanol group – specifically, analysis of single cell data shows that the proportion of anti-regenerative HSC state is not increased at 24 h post-PHx in ethanol-fed rats. Instead, ethanol-fed rats show a similar HSC response to resection as control rats (Benjamini & Hochberg corrected *p*-value = 0.70 for Fisher’s exact test on count data), albeit with more quiescence (29% quiescent in ethanol-fed rats compared to 21% quiescent in control rats). Following resection, ethanol-fed rats also have high proportions of cells in the pro-regenerative state (39%) and mixed state (32%) and no cells in the anti-regenerative state (0%). These results indicate that HSCs may have a dynamic transition insufficiency following resection, resulting in greater numbers of quiescent cells. At 24 h post-PHx, the numbers of cells sampled show this dynamic insufficiency only subtly but analysis of other measurements, such as tissue-scale gene expression, may be able to shed further light on this behavior. The dynamic HSC insufficiency could be due to an altered transition propensity of HSCs from their baseline states or a dynamic insufficiency in intercellular signaling that contributes to suppressed transitions. The larger magnitude differences between ethanol and control HSC population balances at baseline compared to 24 h post-PHx indicates that chronic HSC imbalances may have more of an effect impairing regeneration than any dynamic insufficiencies.

#### Hepatic stellate cell state balance modulates liver regeneration dynamics

**Fig. 7:**
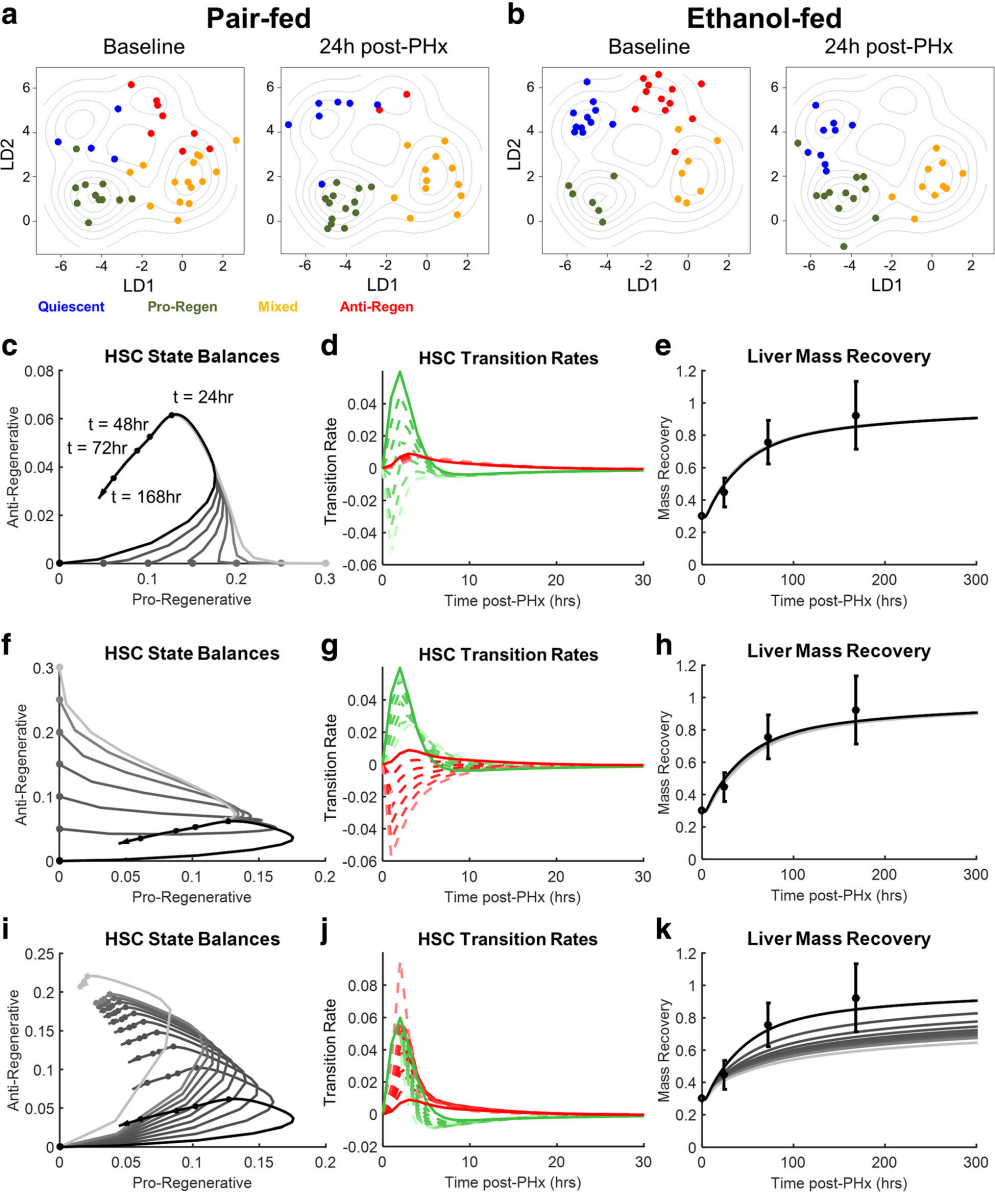
Model analysis shows the impact of imbalances among multiple hepatic stellate cell transcriptional states on liver regeneration dynamics (**a-b**) Topographic map representation of HSC states shows the clustering of HSCs in each condition measured. **c** HSC behavior is constrained prior to 24 h post-PHx even after increasing the baseline fraction of pro-regenerative HSCs. This behavior assumes the absence of other changes associated with changing baseline amounts. **d** Similar to the phase plane behavior, transition dynamics converge prior to 24 h post-PHx. **e** Increasing the baseline amount of pro-regenerative HSCs has little effect on the dynamic regeneration profile. **f** HSC behavior is constrained prior to 24 h post-PHx even after increasing the baseline fraction of anti-regenerative HSCs. This behavior assumes the absence of other changes associated with changing baseline amounts. **g** Similar to the phase plane behavior, transition dynamics converge prior to 24 h post-PHx. **h** Increasing the baseline amount of anti-regenerative HSCs has little effect on the dynamic regeneration profile. **i** Increasing the quiescent to anti-regenerative transition rate leads to a dynamic change in HSC transcriptional state balances. **j** HSC transition dynamics change based on the balance of transition rates. **k** Increasing the quiescent to anti-regenerative transition rate causes a suppressed regeneration profile. This type of profile may be representative of liver function in diseased states, such as liver fibrosis

In addition to investigating the altered balance of HSC states caused by chronic ethanol intake followed by resection, we also investigated how cell-to-cell variability of the transcriptional state of HSCs may be distributed in different conditions (Fig. 7a and b). Both ethanol-fed and control groups have similar within-state cellular variability prior to resection. The exact structure of this variability may not be the same following ethanol treatment, however. For example, the transcriptional space occupied by quiescent cells in control rats appears different from the transcriptional space occupied by quiescent cells in ethanol-fed rats at baseline. Following resection, there appears to be similar variability in both treatments as observed prior to resection.

We sought to interpret our experimental results using our model simulations as a framework to predict the effects of hepatic stellate cell state imbalances on regeneration dynamics. Specifically, we used our model to investigate how altering HSC dynamics could contribute to impaired liver regeneration. We sought to simulate enhanced liver regeneration through altering the baseline balance of cells in the pro-regenerative and anti-regenerative HSC states. We increased the baseline fraction of pro-regenerative HSCs while maintaining the baseline anti-regenerative fraction at zero. Based on model simulations, we predict that increasing the pro-regenerative fraction of HSCs at baseline, in the absence of other changes, results in a rapid evolution of HSC state distributions to match the nominal case (Fig. 7c), which is accomplished through dynamic changes in the pro-regenerative transition rates (Fig. 7d), without yielding a noticeable effect on the mass recovery profile (Fig. 7e). We then sought to simulate impaired regeneration through increasing the baseline amount of cells in the anti-regenerative phenotype, consistent with our experimental results in ethanol-fed rats. Similar to the previous case, changing the baseline level of anti-regenerative HSCs in the absence of other changes results in a constrained evolution of HSC state distribution prior to 24 h post-PHx to eventually follow the nominal trajectory, due to changes in both pro-regenerative and anti-regenerative transition rates (Fig. 7g). As before, this baseline imbalance results in no discernible change the mass recovery profile. The constrained HSC behavior in both cases is likely the result of similar Kupffer cell regulation. Following PHx, simulated Kupffer cells respond identically regardless of initial HSC conditions. Therefore, similar Kupffer cell signaling post-PHx constrains HSC balances towards the nominal case, leading to an effective regeneration response regardless of initial HSC conditions. Neither increasing the initial fraction of pro-regenerative HSCs nor increasing the initial fraction of anti-regenerative HSCs results in a significant change in liver mass recovery dynamics (Additional file 1: Figures S9 and S10). It should be noted that this result should hold true only in the absence of other changes to the liver microenvironment caused by changing the initial balances of cells in each of these cell states.

In contrast, changing the balance of pro-regenerative and anti-regenerative transition propensities (*k*_*Q → PR*_^*HSC*^ and *k*_*Q → AR*_^*HSC*^) results in a shift in the behavior of HSC dynamics post-PHx (Fig. 7i, Additional file 1: Figure S11). By increasing the anti-regenerative transition propensity of HSCs, fewer HSCs transition to the pro-regenerative state and more cells transition to the anti-regenerative state. Furthermore, because of the positive feedback created by the production of TGF-β, HSCs tend to remain in the anti-regenerative state rather than return to quiescence. This leads to a slight decrease in the apparent pro-regenerative transition rate, likely caused by the reduced number of quiescent HSCs due to anti-regenerative transition rate increases (Fig. 7j). This increase in anti-regenerative HSCs leads to a suppressed liver mass recovery post-PHx (Fig. 7k).

### Matrix preconditioning and dynamic hepatic stellate cell transition insufficiency contribute to suppressed liver regeneration

**Fig. 8:**
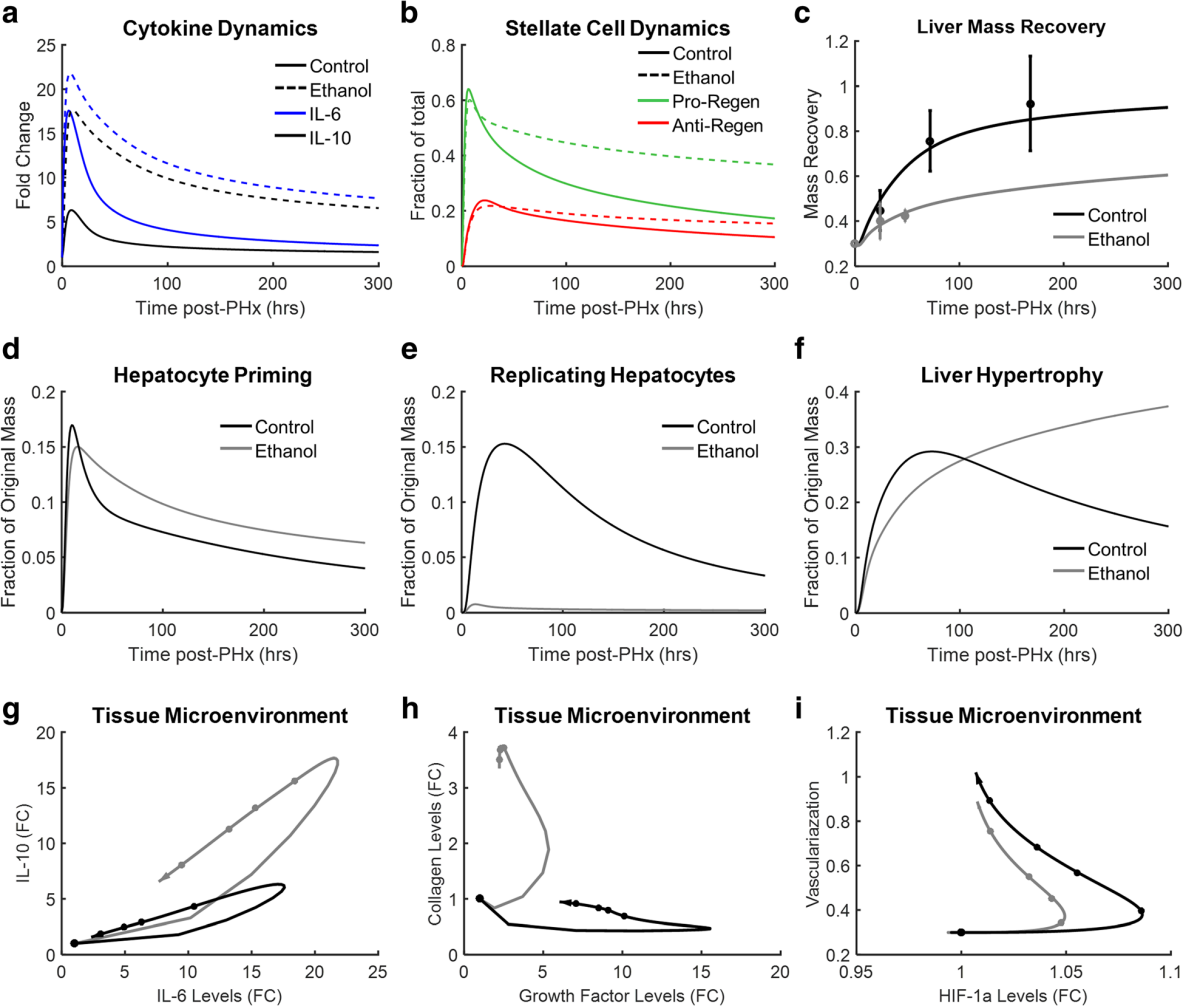
Model prediction of the implications of HSC activation in chronic ethanol-fed rats. **a** Chronic ethanol use leads to increased levels of pro-inflammatory and anti-inflammatory cytokines following PHx. **b** Chronic ethanol appears to lead to a deficient pro-regenerative HSC response following PHx. **c** The effects of increased cytokine production, imbalanced HSC functional states, and changes to the tissue microenvironment combine to suppress regeneration following ethanol adaptation. The simulated regeneration profile of ethanol adapted rats is consistent with results from [38]. **d-f** Hepatocyte response to ethanol feeding and PHx. (G-I) Selected tissue microenvironment responses to ethanol feeding and PHx

Although our single cell gene expression analysis uncovered imbalances in HSC states at baseline as characterizing disease versus control conditions, our simulations suggest that these differences are not sufficient to alter the response to resection. This indicates that the regeneration deficit in the ethanol group may be due to multiple hits that alter HSC behavior: the first hit to precondition the ECM by increasing the fraction of HSCs in the anti-regenerative state at the baseline chronic ethanol-adapted state, and the second hit to decrease dynamically HSC functional state transitions post-resection. If the altered matrix composition in the ethanol-adapted state results in a stiffer or denser matrix, it is possible that growth factors and other matrix-bound factors are less able to intercalate into the matrix to be available to promote cell growth post-resection. Furthermore, such a dense matrix may slow degradation due to metalloproteases. Using our computational model, we investigated whether this matrix preconditioning and dynamic transition insufficiency are sufficient to account for ethanol-induced suppression of liver regeneration by changing parameters consistent with our hypotheses (changes to parameter values are shown in Table 2). We maintained parameter values of parameters related to Kupffer cell activation constant from our previous simulations to maintain ethanol-induced increases in Kupffer cell activation post-PHx, resulting in high levels of IL-6 and IL-10 (Fig. 8a). We then altered parameter values of parameters related to matrix deposition, matrix metalloprotease function, and HSC transition propensity in accordance with our experimental results (Table 2, Additional file 1: Figure S12). We also reduced the value of the parameter governing JAK activation rate in hepatocytes in response to IL-6 because studies have shown that ethanol-adapted hepatocytes have a deficient STAT3-pathway response to cytokine signaling [48, 49]. Tuning these parameter values allows us to match our experimental observations that HSC population levels are dynamically insufficient post-PHx, (Fig. 8b). Based on the simulation results, we postulate that the dynamic insufficiency of HSCs may be due to increased HSC apoptosis in ethanol-adapted rats (Additional file 1: Figure S13). Following the alterations in the parameter values discussed above, simulation results match the experimentally observed mass recovery data following 70% PHx in ethanol-adapted rats (Fig. 8c) [47]. Our simulations capture experimental observations that chronic ethanol intake suppresses hepatocyte priming (experiments show a reduced hepatocyte induction of STAT3 following stimulation by IL-6 following ethanol adaptation) (Fig. 8d), impairs hepatocyte replication post-PHx (experiments show that ethanol-adapted rats had reduced liver mass recovery at 24 and 48 h post-PHx) (Fig. 8e), and results in hepatomegaly (experiments show an alcohol-adaptation dependent increase in hepatocyte volume and protein content, but not cellular DNA content) (Fig. 8f) [47, 48, 50]. Model simulations suggest, however, that hepatocyte size increases early in control rats in response to PHx but that the hepatomegaly in the ethanol group is a delayed response to PHx. Altered cell network behavior in ethanol-adapted animal simulations leads to dramatic differences in tissue microenvironment (Fig. 8g, h & Additional file 1: Figure S12) but little difference in final amount of tissue vascularization (Fig. 8i), suggesting that chronic ethanol consumption does not impair revascularization of the liver. These simulation results, however, are the result of a particular choice of parameter values based on our hypothesis of stellate cell preconditioning of the extracellular matrix in ethanol-adapted rat livers. These results should be considered as a model-based hypothesis for follow-on experimental testing.

**Table 2:** Matrix-associated features predicted in chronic ethanol use and corresponding parameter values

Feature	Control	Ethanol	Parameter	Value in Ethanol (% of nominal)
ECM Density	Sparse	Dense	k_ECM_	400%
ECM Composition	Not enriched in fibrous collagens	Increased fibrous collagens	κ_deg_	10%
Tissue Stiffness	Relatively low	Areas of high stiffness	k_Q → PR_ k_Q → AR_	65% 25%
Availability of Matrix-bound factors	High	Low	K_up_	70%
Growth factor intercalation	High	Low	k_up_	70%

## Discussion

In this work, we present a new framework for studying the control of liver regeneration by the re-distribution of different cell types across multiple functional states and the interaction of cells in a dynamic network. Our modeling analysis indicates that, within this framework, HSCs play an important role governing the dynamics of liver regeneration. We therefore focus our experimental work on exploring HSC behavior during effective and impaired regeneration. Conventionally, HSCs have been assumed to exist in one of two distinct states: quiescent and activated. Quiescent HSCs assist in storage and transport of retinoids as well as in modulating the innate immune response [51–53]. Activated HSCs change their morphology and alter their gene and protein expression profiles to deposit fibrous collagens, causing scarring and worsening fibrosis and cirrhosis [54]. In contrast to this view, our results show a much broader range of transcriptional profiles of HSCs isolated from ethanol-adapted and control livers. Simulations using our computational model showed that the balance of these HSC transcriptional states can affect overall dynamics of liver regeneration. This is in agreement with experimental studies suggesting that HSCs can enhance hepatocyte and HepG2 cell replication, but that livers adapted to chronic diseases characterized by active HSCs (fibrosis and cirrhosis) exhibit impaired regeneration [24, 55–58].

**Fig. 9:**
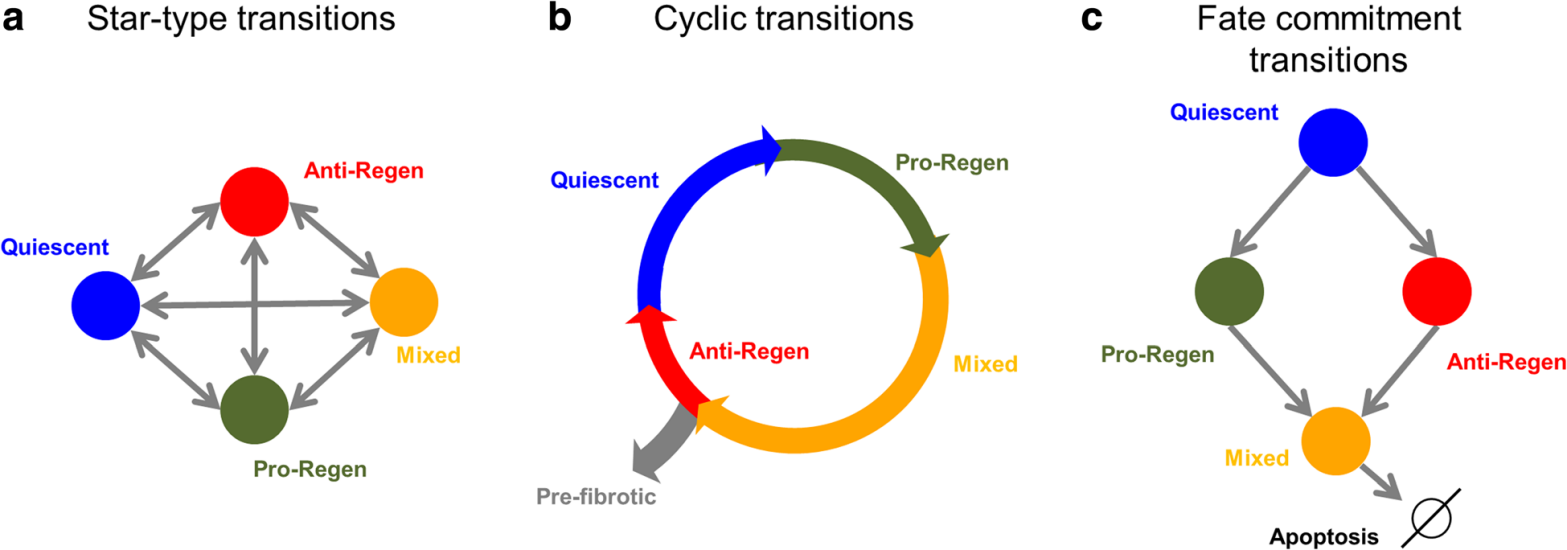
Potential hepatic stellate cell transition patterns. **a** Star-type transitions could allow any functional state to shift its transcriptional profile into any other functional state. **b** A cyclic transition pattern, like the cell cycle, could allow for distributions of cells aiding hepatocyte regeneration or homeostatic renewal. Getting “stuck” in one phase of the cycle could lead to cell exit into apoptosis or pre-fibrotic phenotypes. **c** A cell fate commitment pattern would allow cells to transition only one way. In such a pattern, quiescent cells would have to be continuously replenished. Perhaps hepatic stem cells play a role in this replenishment

Our study shows that HSCs exhibit gene expression profiles that correspond to distinct cellular functional states. What is less clear is the path of progression of HSCs through these functional states. One possibility is that HSCs could exist in a “transcriptional continuum” where cells can transition between any two states in response to internal and external stimuli (Fig. 9a), with the nature of the stimulus governing the type of transition possible. Alternatively, HSCs could progress through a series of states beginning at quiescent state and moving towards an anti-regenerative state (Fig. 9b). In such a scheme, progression to the anti-regenerative state requires transcriptional regulation into the pro-regenerative and mixed states before reaching the anti-regenerative state. Such a progression may correspond to the physiological stages of regeneration, which may mean that such an ordered progression does not necessarily hold true for disease contexts that yield diminished regeneration. It is also possible that HSCs could progress through committed fate transitions from a quiescent phenotype to a mixed phenotype (Fig. 9c). In this scheme, the only way to rebalance the distribution of HSC states is through cell death of terminally differentiated HSCs. Further investigations are needed to identify which, if any, of these hypotheses are correct.

No matter how the progression among HSC states occurs, we postulate that the dynamic transition of HSCs among these states may correspond to the distinct phases of liver regeneration described in the literature. In this scheme, during the priming phase of regeneration (0–12 h post-PHx), HSCs would be predominantly in the pro-regenerative and quiescent states, making GFs available for hepatocyte entry into the cell cycle and remodeling basement matrix to allow for effective regeneration. During the replication phase (12–72 h post-PHx), HSCs would be predominantly in the mixed state, continuing to produce GFs for hepatocyte replication but also actively producing additional scaffolding to aid newly generated hepatocytes. During the termination of regeneration (following ~ 96 h post-PHx), HSCs would be predominantly in the anti-regenerative state, no longer producing GFs but continue to remodel and produce extracellular matrix to allow for hepatocyte migration and mass increase, as well as to allow for revascularization of the tissue. The hypothesis that HSCs exist primarily in the anti-regenerative state during the termination phase is consistent with studies showing that TGF-β and increase in extracellular matrix can both inhibit hepatocyte proliferation. Whether TGF-β signaling (within hepatocytes or in the non-parenchymal cells) or matrix deposition contribute to the termination of regeneration alone, or in combination with other factors upregulated in the anti-regenerative HSC state, remains a topic of active research [59, 60].

Our results show high variability in transcript expression across single cells. This type of cell-scale transcript expression variability is typical and is understood as arising from underlying biological heterogeneity and stochasticity. It has been shown repeatedly that technical variability of sample processing is orders of magnitude lower than biological variability across a wide range of assay techniques, including single-cell, high-throughput qPCR [7], single-cell RNA-seq [6], and single-cell protein hybridization and quantification [61]. A key challenge to exploring single-cell-scale behavior is to isolate an appropriate number of cells that enables a meaningful analysis of underlying cell states. Some cell types can be isolated more easily than others and with fewer processing steps that influence RNA measures. One of the earliest single cell studies used ~ 150–200 single cells derived from the earliest stages of embryo development for analysis of cells states using high-throughput, real-time PCR of a select set of genes in each cell [62]. Our experimental efforts utilized a similar number of cells and evaluated the expression of several genes relevant to HSC function. Recent studies have started to investigate thousands of single cells at a time [63–67], with some studies using fewer cells [68, 69], while a few studies using many more cells [11, 70]. We employed laser capture microdissection to isolate HSCs from flash-frozen tissue. This approach limits the sample throughput in order to preserve in vivo cellular state as compared to isolation techniques that require tissue perfusion [7, 71]. It should be noted that several of the studies investigating single cells utilize tissues with multiple cell types, requiring hundreds of cells to identify cell states within each cell type. In our study, the use of laser capture microdissection allowed us to target one specific cell type and diminished the need for capturing hundreds of single cells to conduct analysis of cells states. Notably, previous studies have shown that data from even a low number of single cells can be used to infer the distribution of cell states underlying the overall tissue response [62].

Several opportunities exist to extend our current study. Follow-on experimental efforts could build on the initial evidence on the shifting distributions of hepatic stellate cell states based on select genes by comprehensively characterizing the transcriptome using RNA-seq and related global assays. It should be noted, however, that even when considering transcriptomic data, only a fraction of the transcripts (a few tens to hundreds, depending on the context) contribute to separation of functional states, as evidenced by typical factorization analysis of these data sets using strategies such as Principal Component Analysis, Multi-dimensional Scaling, and Stochastic Embedding [1, 2, 4, 10]. The identity of functional states depends on the subset of the transcriptome that is considered as relevant to a specific context under study. For example, fractionation of cell states based on metabolic pathways may yield a different hierarchy of cell states than fractionation based on transcription factors, cell surface receptors, signaling pathway components, or a combination thereof. Our analysis of cellular functional states based on select set of approximately 100 transcripts could be considered as parallel to single cell cytometry studies of select (~ 20–100) proteins using the CyTOF approach that have uncovered new insights into the cellular hierarchy of multiple cell types, including immune cells and stem cells [72–74].

Another opportunity is to extend the computational model to incorporate our novel experimental data describing single HSC gene expression. The model includes only three HSC states, excluding the mixed HSC state identified as a major contributor to HSC population balances. Accounting for this additional state, however, may require a more thorough understanding of how HSCs transition across these states. To this end, future investigations need to develop temporally informative data, for example through cell lineage tracing techniques, to identify the dynamics governing progression of individual HSCs through different states. Additionally, we considered hepatocyte replication following resection as a uniform property of all hepatocytes, although recent studies have begun to appreciate the contributions of liver “stem cell-like” cells contributing to regeneration [75, 76]. Our combined single-cell based transcriptional analysis and computational modeling approach could be a powerful tool to investigate the contributions of these “stem cell-like” hepatocytes as well as additional hepatocyte transcriptional states to liver regeneration and dynamic liver function. Further extensions of our approach could be used to study the relationship between spatial heterogeneity in the liver and regeneration. Using our approach, we collected single cells from portal and central regions of multiple liver lobes and record exact spatial information about each cell. Coupling such data with a spatially resolved cell network model would allow for in-depth investigation into how spatial heterogeneity affects regeneration and vice versa.

## Conclusions

Our study has several implications for studying the mechanisms driving chronic liver diseases such as fibrosis, cancer, and others. Our results demonstrate that the dynamic liver function is governed by multiple levels of physiological controls: molecular, intracellular, and inter-cellular networks. Molecular control of liver function has been widely studied, but has yet to progress to promising therapies for severe liver disease [77, 78]. Previous studies have focused on the effects of canonical cell types interacting or the effects of other organs, such as adipose tissue, on liver function [57, 79]. Our work takes a different approach, using a data-driven understanding of cell functional states to gain insights into how the dynamic distributions of cells in various functional states contribute to overall tissue function. In summary, our study presents an integrated experimental and computational modeling approach towards understanding the quantitative and dynamic contribution of constituent cell states and their interactions to overall tissue function, with broad application to study the impact of chronic conditions and disease on response to acute physiological perturbations.

## Methods

### Animal use

All animal studies were approved by the Institutional Animal Care and Use Committee (IACUC) at Thomas Jefferson University. Jefferson’s IACUC is accredited by the Association for Assessment and Accreditation of Laboratory Animal Care and experiments were designed using the Guide for the Care and Use of Laboratory Animals.

Adult (8–10 week old) Sprague-Dawley rats were subjected to a standard Lieber-DeCarli pair feeding protocol with 36% of calories provided by ethanol or carbohydrates (maltodextrin). Following 5–7 weeks of ethanol feeding, rats were anesthetized and subjected to 70% PHx by surgical removal of medial and left lateral lobes as per standard procedure [80, 81]. The medial and left lateral lobes were quickly frozen in OCT blocks (TissueTek, QIAGEN, Valencia, CA) over a dry ice and methanol bath to serve as within-animal, 0 h controls. At 24 h post-PHx, rats were again anesthetized and the remnant liver tissue was excised and frozen as before. Following excision of the remaining liver mass, rats were sacrificed by cervical dislocation. Tissue was stored at − 80 °C until further use.

### Immunohistochemical staining

Frozen liver tissue in OCT blocks was sectioned 10 μm thick using a cryostat set at − 20 °C and thaw mounted on glass slides. Sliced tissue was stored at − 80 °C for up to 2 weeks. Immunohistochemical staining was performed using a rapid staining protocol taking approximately 30 min to complete to preserve RNA integrity. Slides were first fixed in cold acetone and hydogen peroxide (Sigma-aldrich, St. Louis, MO, 50 ml: 50 μl) for 30 s, then blocked and permeabilized with PBS containing 2% BSA (Sigma–Aldrich, St. Louis, MO) for 1 min. Afterwards, liver sections were incubated with the primary antibody anti-glial fibrillary acidic protein (Gfap) (ab4674, Abcam, Cambridge, MA), an HSC marker, for 4 min at room temperature. Then the slides were washed, and were incubated for 4 min at room temperature in the dark with the secondary antibody Cy3 anti-chicken 1/200 with DAPI 1/10,000, phalloidin 2.5/100, and PBS containing 2% BSA. Then slides were rinsed with PBS and dehydrated in graduated ethanol concentrations (70–100%) and in xylene for 5 min.

### Laser capture microdissection (LCM)

The LCM process was performed using a PixCell system and CapSure Macro LCM caps (Arcturus Engineering, Mountain View, CA). Single cells or 10 cell pools of cells with positive staining (GFAP+) were lifted individually on caps. HSCs from the first 7 layers of hepatocytes around the portal or central vein were lifted and their position was annotated for future analyses. The annulus for the Laser was adjusted to the size of HSCs (approximately 10 μm, HSCs were lifted and screened for quality as a whole cell on the cap after capture and only accepted if the cell target was fully lifted. During single cell sampling both the tissue and the corresponding cap were inspected for the removed cell body to ensure that the fluoresced HSC of interest is collected. Lysis buffer was added onto the single cell on the cap (5.5 μl; Life Technologies, Grand Island, NY) and cooled on ice before storage at − 80 °C. Hepatocytes, which stained negative for Gfap (Gfap-) and were discernable by size and morphology, were collected according to the same procedure.

### High-throughput quantitative PCR

Our sample preparation calls for processing the single cells directly in a reverse transcriptase reaction rather than extracting RNA. Following reverse transcriptase reactions, cells were subjected to realtime PCR for targeted amplification and detection using primers designed to target specific genes using PrimerBlast [82]. Official gene symbols (from the Nucleotide database of NCBI) were used to denote target genes throughout the manuscript. Refseq IDs are available in Additional file 1: Table S2. Where possible, primers were designed with intron-spanning PCR primers (Primer sequences can be found in Additional file 1: Table S2). The standard BioMark™ protocol was used to pre-amplify cDNA samples for 22 cycles using TaqMan® PreAmp Master Mix per the manufacturer’s protocol (Applied Biosystems, Foster City, CA). qPCR reactions were performed using 96.96 BioMark™ Dynamic Arrays (Fluidigm®, South San Francisco, CA) enabling quantitative measurement of multiple mRNAs and samples under identical reaction conditions (Spurgeon et al. 2008). Each run consisted of 40 amplification cycles (15 s at 95 °C, 5 s at 70 °C, 60s at 60 °C). CT values were calculated by the Real-Time PCR Analysis Software (Fluidigm). Four 96.96 BioMark™ Arrays were used to measure gene expression across the ~ 300 single cell samples included. The same serial dilution sample set was included to verify reproducibility and test for technical variability.

### Data availability

Raw and processed data, code to process the data, and the mathematical model used to interpret the data are available as additional files (Additional files 2, 3 4 and 5).

### Data normalization

Individual qRT-qPCR reactions were examined to ensure the quality of each qRT-PCR reaction. Each reaction was manually passed or failed based on the qualitative nature of the reaction curves obtained from the PCR. Any reaction below the limit of detection based on the CT-value of DNA suspension buffer undergoing qPCR procedure (no template control) was manually failed. Following this pass/fail analysis, samples having greater than 25% failed reactions and gene assays having greater than 80% failed reactions were excluded from the present analysis. This exclusion step further increases the quality and confidence in the data used for analysis. A total of 139 single cell samples (36 Ethanol 0 h, 31 Ethanol 24 h, 38 Control 0 h, and 34 Control 24 h) and 72 different gene assays were included in the present analysis.

Data was normalized using a modified -ΔΔC_T_ method [83]. The median of the highest quality genes (pass in greater than 45% of samples) was used as a pseudo-housekeeping gene to account for differences in cell size, incomplete cell lifting, and BioMark™ assay loading variation. We normalized the raw C_T_ values by subtracting them from the pseudo-housekeeping gene value on a per sample basis. We then removed any effects of relative expression levels across genes by median centering the expression value of each gene to give our normalized -ΔΔC_T_ value for each reaction.

### Cell type specificity analysis

Markers for four cell types were included in our qRT-PCR assays: Apoa4 for hepatocytes, Itgam for Kupffer cells, Pecam1 for endothelial cells, and Actb and Gfap for HSCs. Each individual hepatocyte or HSC captured expressed high levels of its marker gene(s) and low levels of marker genes for other cell types, often below the limit of detection (Fig. 4b). We measured 42 gene assays in both hepatocytes and HSCs using two BioMark™ Arrays, one for each cell type. Batch effects were removed by normalizing the dilution curves for the overlapping gene assays to each other. This strategy could be used because the same dilution series was used for both arrays. We then imputed missing data as the minimum value for each assay minus 1 CT (to approximate the limit of detection). This strategy of imputing missing values as equal to the limit of detection was used because visual inspection of our data suggests that failed reactions were often caused by transcripts being expressed below the limit of detection rather than other causes of reaction failure. We then performed principal component analysis (PCA) on the collated raw CT values from these cells to identify using an unsupervised method if cell type was a major contributor to the variability in the data (Fig. 4c). This analysis also allowed for us to identify genes that contribute significantly to the variability in the data (Fig. 4d). Similar results were found when analyzing 10-cell pools of HSCs and individual hepatocytes from the same BioMark™ array.

### Linear discriminant analysis

High fidelity samples and assays were identified using a threshold of > 30% of assays working per sample, and > 10% of cells expressing each gene. Gene expression identified as below the limit of detection was called as “NA” in the original data, but we imputed values below the limit of detection by assuming each non-expressed gene had a CT value of the maximum (for that gene) plus one, indicating that the gene is at least 2-fold below the limit of detection (see above).

Linear discriminant analysis was then performed on these data using the ‘MASS’ package in the computing language R [84–86].

### Silhouette score analysis

We calculated the silhouette score of the clusters identified in our linear discriminant analysis to quantify the separation of the identified clusters. We used the ‘MASS’ package in R for this calculation [84]. We then randomized our data 1000 times and re-performed our linear discriminant analysis and silhouette score calculation. We calculated an empirical *p*-value for our silhouette score by finding the number of random scores equal to or greater than that calculated for our data.

### Minimum spanning tree analysis

We calculated minimum spanning trees to identify a possible progression of activation of single HSCs using Euclidean distance in the *spantree* function from the package ‘*vegan*’ in R [87]. Minimum spanning trees are a graphical approach that connects all nodes (single cells) in a data set that maintains a minimum weight between edges, where weight is a measure of unfavorable connections. Highly correlated nodes are connected by edges, making this technique appropriate to hypothesize progression of a single cell through highly correlated nodes.

### Topological maps

Topological maps were produced from 2-dimensional kernel densities of all HSCs in the linear discriminant space using the R package MASS [84].

### Model development rationale

#### Metabolic demand

Our model uses a lumped parameter, the metabolic demand (M), to represent the cellular effects of the physiological state of the animal. The metabolic demand can be viewed as the cellular response to the normal stress put on a healthy liver to maintain physiological functions. It is likely related to a combination of external factors such as portal blood flow, portal pressure, nutrient availability, toxin flux (such as lipopolysaccharide) and intrinsic factors including hepatocyte metabolic capacity, functional history, and transcriptional state. Each cell type could have a different value for M, as each cell type could integrate the effects of these physiological effects differently. In a model describing individual cells, each cell could have a different value for M, based on its history, state, and cell type. In our model, each cell type has its own value for M. The metabolic load (defined as metabolic demand per cell, or M/N, where N is the functional mass of the liver) increases following partial hepatectomy and is considered to be the driving force for liver regeneration.

#### Hepatocytes

The simulated hepatocyte state transition network in our model follows the framework presented by [23], in which hepatocytes can exist as quiescent, primed, or replicating (Fig. 1a). IL-6 acting through the JAK-STAT signaling cascade initiates immediate early (IE) gene signals in hepatocytes that catalyze their transition from the quiescent state to the primed state (Fig. 1b, Hepatocytes). Primed hepatocytes can either return to quiescence, which occurs at a constitutive rate, or primed hepatocytes can enter the replicating state in response to sufficient levels of growth factors (GF) produced by non-parenchymal cells and liberated from the ECM. However, hepatocyte replication is inhibited by increased levels of TGF-β [88]. Replicating hepatocytes double their number approximately every 30 h, and return to quiescence at a constitutive rate, which can be increased by ECM buildup. Hepatocytes responding to PHx (i.e. hepatocytes in the primed and replicating states) are also able to increase mass to respond to an increased metabolic demand when the latter drives liver regeneration. Such a framework allows a system representation where hepatocytes can respond to metabolic challenges through replication and through increasing functional capacity (mass) of each cell. This framework is also applicable in disease contexts, where replication may be impaired, providing a capability for hepatocytes to ameliorate damage to tissue in the absence of a regeneration response. Other models have been developed that include cell hyperplasia by representing primed and replicating hepatocytes with discrete size increases instead of allowing for a continuum of hyperplasia [89]. However, these models have not yet been shown to be able to capture disease-specific dynamics of liver response to resection.

In addition, within our framework, hepatocytes are able to sense and respond to hypoxia [90]. During liver regeneration, hepatocytes may begin hypoxic signaling when they have expanded into areas that are not yet vascularized fully [91]. In our model, hypoxic signaling is considered to occur as follows: hepatocytes respond to a low vascularization by inducing Hypoxia Inducible Factor 1α (HIF-1α), which in turn induces hepatocytes production of VEGF and therefore non-parenchymal cell replication to replace lost tissue architecture [92].

#### Kupffer cells

Kupffer cell activation during liver regeneration and in response to chronic and acute stresses has been studied extensively [47, 93–96]. Our model accounts for two functional states of Kupffer cells: quiescent and active (Fig. 1a). In our simulations, active Kupffer cells can transition along a gradient of M1 to M2 activation in response to the autocrine feedback of the cytokines they produce. Activated Kupffer cells return to quiescence at a constitutive rate (Fig. 1b). In our model, Kupffer cells are activated in response to an increased metabolic load; and once activated, they begin to produce cytokines associated with an M1 activation phenotype, high production of IL-6 and Tumor Necrosis Factor α (TNF-α), and low production of Interleukin 10 (IL-10) and TGF-β [95]. TNF-α interacts with several receptors to mediate a variety of physiological responses in Kupffer cells. TNF-α further increases numbers of activated Kupffer cells within a population by binding to TNF receptors (TNFRI and TNFRII) leading to NF-κB activation, transcription of NF-κB target genes (including IL-6, IL-10), immediate early (IE) gene expression, and Kupffer cell activation [97, 98]. TNF-α also induces the production of AP-1 and its downstream targets, including osteopontin (OPN or SPP1), IL-10, and GM-CSF, which induce production of TGF-β in Kupffer cells [99–101]. We model the effects of these pathways by simulating activated Kupffer cells as constitutively expressing TNF-α, IL-6, IL-10, and TGF-β. Kupffer cell activation is also modulated by negative feedback, most prominently by IL-10 impeding TNF-α production [102]. Our model includes a description of IL-10 antagonism of TNF-α production. However, biologically, not even the highest possible levels of IL-10 can inhibit TNF-α production completely; therefore, our mathematical model allows for a nominal amount of TNF-α production even at high levels of IL-10 [103].

Kupffer cells respond to other external factors in addition to activation signals. In response to poor vascularization, HIF-1α is activated in Kupffer cells, leading to production and secretion of PDGF [92]. Activated Kupffer cells also respond to external VEGF, produced by hepatocytes. VEGF induces Kupffer cells to enter a replicating state, which results in a cell doubling in ~ 30 h [104]. Replicating Kupffer cells return to the activated state at a constitutive rate, which is increased by ECM buildup.

#### Hepatic stellate cells

Much work has focused on characterizing HSC activation in the context of fibrotic or pro-fibrotic states, several studies have also found that HSCs contribute to liver regeneration through the production of growth factors that enhance regeneration, such as HGF, EGF, and FGF [105, 106]. These disparate HSC functions led us to postulate the existence of two mutually exclusive HSC functional states: a pro-regenerative state, producing growth factors, and an anti-regenerative state, producing collagens and TGF-β (Fig. 1a).

In our model, HSCs are considered as transitioning into distinct states in response to distinct external stimuli. IL-6 signals through the JAK-STAT signaling cascade to induce production of growth factors, such as HGF and FGF, and immediate early (IE) genes, which transition HSCs to a pro-regenerative phenotype. These interactions are represented in our model as a physiological transition between states (Fig. 1b). Studies investigating contributions of non-parenchymal cells to liver regeneration [24] have hinted at IL-6 induction of this pro-regenerative state. HSC transition to an anti-regenerative state, in contrast, occurs through TGF-β signaling via the SMAD2/3 pathway, considered in our model as a state transition (Fig. 1b). The functional effects of this anti-regenerative state on the liver regeneration dynamics were matched to the known effects of pro-fibrotic HSC activation on hepatocyte proliferation and liver regeneration, which have been investigated rigorously in vitro and in vivo [107, 108].

In our modeling framework, HSCs can enter a replicating phenotype from either the pro-regenerative or the anti-regenerative states in response to increased PDGF levels. Replicating HSCs transition from their replicating states back to their respective functional states (pro-regenerative or anti-regenerative) at a constitutive rate and a matrix-dependent rate. These matrix-dependent rates are increased by ECM buildup, leading to a reduction in levels of HSCs replicating.

#### Tissue-scale response

Maintenance of ECM and ECM-bound factors involves the coordination of several cell types to control the composition and properties of the ECM. Many cell types within the liver contribute to matrix degradation both through constitutive degradation and through active degradation using matrix metalloproteases (MMPs) [24, 109–112]. Consequently, we modeled matrix degradation as a cell-independent process. Intrinsic matrix degradation was modeled as a constitutive rate of ECM removal, and extrinsic matrix degradation as TNF-α inducing MMP production, which increases ECM removal. The matrix itself is an active contributor to liver regeneration by sequestering and releasing growth factors during tissue homeostasis and during the regeneration response. Our model includes a matrix contribution to growth factor production by implicitly representing GF produced by HSCs as coming directly from these cells and from freed matrix-bound factors. Matrix sequestration of growth factors is included explicitly via a term that allows GF to be bound and sequestered by ECM.

Vascularization was modeled at the physiological scale by introducing a parameter representing the extent of vascularization within the tissue (i.e., the total volume of blood vessels and their distribution within the tissue). The extent of vascularization increases in response to the amount of VEGF produced by hepatocytes themselves responding to hypoxia. As vascularization increases, hypoxia decreases, causing VEGF levels to return to baseline. This feedback mechanism acts to maintain vascularization levels in the regenerating liver.

### Computational model description

Our computational model was extended from [23]. The previous model included only a linear response of non-parenchymal cells to resection. Our extended model explores non-parenchymal cell behavior more fully. We maintained the original model architecture and hepatocyte equations. Compared to the previous model [23], our new model introduces 15 new differential equations describing 2 new cell types and tissue vascularization and 67 new parameters describing non-paranchymal cell activation and signaling. All simulations were performed in Matlab (Mathworks, Natick, MA) using ODE15s as our ODE solver. We found that ODE23s gave similar results. All the simulation results included in the manuscript were generated using ODE15s.

#### Hepatocyte equations

Our extended model maintains the framework of the previously published initial model by allowing hepatocytes to exist in one of three states: Quiescent (Q), Primed (P), or Replicating (R). Factors produced by non-parenchymal cells in response to liver metabolic load (metabolic demand per cell or M/N) shift hepatocytes between states, according to the following equations.

1 $$ \frac{d}{dt}Q=-{k}_{QP}\left(\left[I\mathrm{E}\right]-\left[I{E}_0\right]\right)Q+{k}_{RQ}\left[ ECM\right]R+{k}_{req}{\sigma}_{req}P-{k}_{ap}{\sigma}_{ap}Q $$

2 $$ \frac{d}{dt}P={k}_{QP}\left(\left[ IE\right]-\left[I{E}_0\right]\right)Q-{k}_{PR}\left(\left[ GF\right]-\left[G{F}_0\right]\right)P-{k}_{req}{\sigma}_{req}P-{k}_{ap}{\sigma}_{ap}P $$

3 $$ \frac{d}{dt}R={k}_{PR}\left(\left[ GF\right]-\left[G{F}_0\right]\right)P-{k}_{RQ}\left[ ECM\right]R+{k}_{prol}R-{k}_{ap}{\sigma}_{ap}R $$

Where [IE] represents the concentration of immediate early genes expressed in response to STAT-3 transcriptional regulation and [ECM] represents the amount of extracellular matrix. σ_ap_ and σ_req_ are sigmoidal functions defined as:

4 $$ {\sigma}_{ap}=0.5\ast \left(1+\tanh \left(\frac{\left({\theta}_{ap}-\raisebox{1ex}{$N$}\!\left/ \!\raisebox{-1ex}{$M$}\right.\right)}{\upbeta_{\mathrm{ap}}}\right)\right) $$

5 $$ {\sigma}_{req}=0.5\ast \left(1+\tanh \left(\frac{\left({\theta}_{req}-\left[ GF\right]\right)}{\upbeta_{\mathrm{req}}}\right)\right) $$

The parameters β and θ in each of these equations are tuned so that when metabolic load is high, σ_ap_ is high; conversely, when [GF] is high, σ_req_ is low. Therefore, when cells are highly stressed (high metabolic load), apoptosis occurs at a high rate; when GFs are available, cells remain in the “Replicating” state.

The JAK-STAT signaling pathway, GF production, and ECM production are modeled as a combination of first order and Michaelis-Menten kinetics, as shown in the following equations.

6 $$ \frac{d}{dt}\left[ JAK\right]=\frac{V_{JAK}\left[ IL6\right]}{\left[ IL6\right]+{k}_M^{JAK}}-{\kappa}_{JAK}\left[ JAK\right]+{k}_2 $$

$$ \frac{d}{dt}\left[ STAT3\right]=\frac{V_{ST3}\left[ JAK\right]{\left[ proSTAT3\right]}^2}{{\left[ proSTAT3\right]}^2+{k}_M^{ST3}\left(1+\left[ SOCS3\right]/{k}_I^{SOCS3}\right)} $$

7 $$ -\frac{V_{IE}\left[ STAT3\right]}{\left[ STAT3\right]+{k}_M^{IE}}-\frac{V_{SO\mathrm{C}S3}\left[ STAT3\right]}{\left[ STAT3\right]+{k}_M^{SO CS3}}-{\kappa}_{ST3}\left[ STAT3\right]+{k}_3 $$

8 $$ \frac{d}{dt}\left[ SOCS3\right]=\frac{V_{SOCS3}\left[ STAT3\right]}{\left[ STAT3\right]+{k}_M^{SOCS3}}-{\kappa}_{SOCS3}\left[ SOCS3\right]+{k}_4 $$

9 $$ \frac{d}{dt}\left[ IE\right]=\frac{V_{IE}\left[ STAT3\right]}{\left[ STAT3\right]+{k}_M^{IE}}-{\kappa}_{IE}\left[ IE\right]+{k}_5 $$

10 $$ \frac{d}{dt}\left[ GF\right]={k}_{GF}\frac{M^{hepatocyte}}{N}-{k}_{up}\left[ GF\right]\left[ ECM\right]-{\kappa}_{GF}\left[ GF\right]+{k}_7 $$

11 $$ \frac{d}{dt}\left[ ECM\right]=-{k}_{deg}\left[ IL6\right]\left[ ECM\right]-{\kappa}_{ECM}\left[ ECM\right]+{k}_6 $$

Where [proSTAT3] represents the concentration of monomeric STAT-3 available to dimerize following IL-6 signaling.

Hypoxia inducible factor (HIF-1α or HIF) is modeled as increasing when the liver mass increases faster than vascularization. HIF-1α then stimulates VEGF production within hepatocytes.

12 $$ \frac{d}{dt}\left[ HIF\right]=\frac{\left(N-{N}_0\right)}{HL}{k}_{HIF}\left(1-\tanh \left(3\ast Vascularization\right)\right)-\frac{V_{VEGF}\left[ HIF\right]}{K_M^{VEGF}+\left[ HIF\right]}-{\kappa}_{HIF}\left[ HIF\right]+{K}_{SS}^{HIF} $$

Where HL is the tissue hypoxia load or tissue oxygen demand, k_HIF_ is the maximum HIF production rate, the 3 in the tanh function is a scaling term to ensure HIF production stops when the vascularization is appropriate for the organ mass, V_VEGF_ and K_M_^VEGF^ are the Michaelis-Menten parameters for VEGF production from HIF, κ_HIF_ is the degredation rate of HIF, and K_SS_^HIF^ is the steady state HIF production of the organ. VEGF production from HIF in hepatocytes is modeled according to Michaelis-Menten kinetics.

13 $$ \frac{d}{dt}\left[ VEGF\right]=\frac{V_{VEGF}\left[ HIF\right]}{K_M^{VEGF}+\left[ HIF\right]}-{\kappa}_{VEGF}\left[V\mathrm{E} GF\right]+{K}_{SS}^{VEGF} $$

Where κ_VEGF_ is the degradation rate of VEGF and K_SS_^VEGF^ is the steady-state VEGF production.

One of the unique characteristics of regenerating hepatocytes is that they maintain (at least to a large extent) their capacity for healthy liver metabolism and tissue function while undergoing a proliferation response to resection. As a result of this, the overall cellular response to signaling is coupled to total number of hepatocytes. In our model, the molecular signals governed by eqs. 6–13 depend on the total amount of liver mass, N (eq. 14). The liver mass, N, is considered as primarily a function of hepatocytes in different states (eqs. 1–3), along with a growth variable accounting for hypertrophy (eq. 15). Hence, the cellular and molecular state variables are interlinked in both directions.

#### Tissue equations

The overall hepatocyte mass, N, includes hypertrophy and cell growth (hyperplasia) of primed and replicating cells in response to metabolic load as follows:

14 $$ N=Q+G\left(P+R\right) $$

Where G represents the relative cell mass, which is initially set to 1 and changes according to the following equation:

15 $$ \frac{dG}{dt}={k}_{growth}\left(\frac{M^{hepatocyte}}{N}-\frac{M^{hepatocyte}}{N_0}\right) $$

Where N_0_ is the initial fraction prior to resection (set to a value of 1 in our simulations) and k_growth_ is a species-specific cell growth rate representing how quickly a single cell can increase mass.

Vascularization is promoted by increased levels of VEGF and proceeds through a phenotypic rate (k_vas_).

16 $$ \frac{d}{dt}\left[ Vascularization\right]={k}_{vas}\left(\left[ VEG F\right]-\left[ VEG{F}_0\right]\right) $$

IL6 and TGFβ can be produced by multiple cell types, thus the composite equations for these species is shown below, where the definitions of the terms contained in the equations are shown in the following respective cell type sections.

17 $$ \frac{d}{dt}\left[ IL6\right]={k}_{IL6}^{hepatocyte}\frac{M^{hepatocyte}}{N}-\frac{V_{JAK}\left[ IL6\right]}{\left[ IL6\right]+{k}_M^{JAK}}+{K}_{SS- hepatocyte}^{IL6}+{k}_{IL6}^{KC}{A}_{KC}-{\kappa}_{IL6}\left[ IL6\right]+{K}_{SS- KC}^{IL6} $$

18 $$ \frac{d}{dt}\left[ TGF\beta \right]={k}_{TGF}^{KC}{A}_{KC}+{K}_{SS- KC}^{TGF}+{k}_{TGF}^{HCS}A{R}_A-{\kappa}_{TGF}\left[ TGF\beta \right]+{K}_{SS- HSC}^{TGF} $$

#### Kupffer cell equations

Kupffer cells were modeled to exist in one of three phenotypic states: Quiescent, Active, and Replicating. Kupffer cells become active initially due to physiological cues and early signaling events post-PHx, which we modeled using a lumped parameter (similar to the hepatocyte equations above). In addition to physiological signals, Kupffer cell states are governed by molecular parameters.

19 $$ \frac{d}{dt}{Q}_{KC}=-{k}_{QA}\left[\left(\left[ TN F\right]-\left[ TN{F}_0\right]\right)+\left(\frac{M^{KC}}{N}-M\right)\right]{Q}_{KC}+{k}_{req}{\sigma}_{req}{A}_{KC}-{k}_{ap}{\sigma}_{ap}{Q}_{KC} $$

20 $$ \frac{d}{dt}{A}_{KC}={k}_{QA}\left[\left(\left[ TN F\right]-\left[ TN{F}_0\right]\right)+\left(\frac{M^{KC}}{N}-{M}^{KC}\right)\right]{Q}_{KC}+{k}_{RA}\left[ ECM\right]{R}_{KC}-{k}_{req}{\sigma}_{req}{\mathrm{A}}_{KC}-{k}_{AR}\left(\left[ VEG F\right]-\left[ VEG{F}_0\right]\right){A}_{KC}-{k}_{ap}{\sigma}_{ap}{A}_{KC} $$

21 $$ \frac{d}{dt}{R}_{KC}={k}_{AR}\left(\left[ VEG F\right]-\left[ VEG{F}_0\right]\right){A}_{KC}-{k}_{RA}\left[ ECM\right]{R}_{KC}+{k}_{rep}{R}_{KC}-{k}_{ap}{\sigma}_{ap}{R}_{KC} $$

Where Q_KC_, A_KC_, and R_KC_ represent quiescent, activated, and replicating Kupffer cells, respectively. Requiescence, apoptosis, and replication are governed similar to hepatocytes with [VEGF] replacing [GF] in the sigmoidal requiescence function, see eq. 5. Once activated, Kupffer cells secrete multiple molecules at different rates.

22 $$ \frac{d}{dt}\left[ TNF\right]={k}_{TNF}{A}_{KC}\left(\frac{k_{TNF}^{Nom}+\left[ IL10\right]}{\left[ IL10\right]}\right)-{\kappa}_{TNF}\left[ TNF\right]+{K}_{SS}^{TNF} $$

23 $$ \frac{d}{dt}\left[ IL10\right]={k}_{IL10}{A}_{KC}-{\kappa}_{IL10}\left[ IL10\right]+{K}_{SS}^{IL10} $$

Each protein is produced at a phenotypic rate *k*_*xx*_, where xx is the species of interest. Each protein is degraded according for first order kinetics at a rate κ_xx_ and produced or degraded at a steady-state rate of K_ss_^xx^. In addition, the production of [TNF] is slowed by [IL-10] such that when [IL-10] is close to its initial value of 1, [TNF] is produced at a nominal rate according to *k*_*TNF*_^*Nom*^.

#### Hepatic stellate cell equations

HSCs were simulated as existing in a quiescent state, two active states (pro-regenerative and anti-regenerative), and two replicating states (one from each activation state). Shifts between these states are catalyzed by molecular abundances, as shown in the equations below.

24 $$ \frac{d}{dt}{Q}_{HSC}=-{k}_{Q\to PR}\left(\left[ IL6\right]-\left[ IL{6}_0\right]\right){Q}_{HSC}+{k}_{PR\to Q}{\sigma}_{req}P{R}_A-{k}_{Q\to AR}\left(\left[ TGF\beta \right]-\left[ TGF{\beta}_0\right]\right){Q}_{HSC}+{k}_{AR\to Q}{\sigma}_{req}A{R}_A-{k}_{ap}{\sigma}_{ap}{Q}_{HSC} $$

25 $$ \frac{d}{dt}P{R}_A={k}_{Q\to PR}\left(\left[ IL6\right]-\left[ IL{6}_0\right]\right){Q}_{HSC}-{k}_{PR\to Q}{\sigma}_{req}P{R}_A+{k}_{PR R\to PR}\left[ ECM\right]P{R}_R-{k}_{PR\to PR R}\left(\left[ PDG F\right]-\left[ PDG{F}_0\right]\right)P{R}_A-{k}_{ap}{\sigma}_{ap}P{R}_A $$

26 $$ \frac{d}{dt}P{R}_R={k}_{PR\to PR R}\left(\left[ PDG F\right]-\left[ PDG{F}_0\right]\right)P{R}_A-{k}_{PR R\to PR}\left[ ECM\right]P{R}_R+{k}_{prol}P{R}_R-{k}_{ap}{\sigma}_{ap}P{R}_R $$

27 $$ \frac{d}{dt}A{R}_A={k}_{Q\to AR}\left(\left[ TGF\beta \right]-\left[ TGF{\beta}_0\right]\right){Q}_{HSC}-{k}_{AR\to Q}{\sigma}_{req}A{R}_A+{k}_{AR R\to AR}\left[ ECM\right]A{R}_R-{k}_{AR\to AR R}\left(\left[ PDG F\right]-\left[ PDG{F}_0\right]\right)A{R}_A-{k}_{ap}{\sigma}_{ap}A{R}_{\mathrm{A}} $$

28 $$ \frac{d}{dt}A{R}_R={k}_{AR\to AR R}\left(\left[ PDG F\right]-\left[ PDG{F}_0\right]\right)A{R}_A-{k}_{AR R\to AR}\left[ ECM\right]A{R}_R+{k}_{prol}A{R}_R-{k}_{ap}{\sigma}_{ap}A{R}_R $$

Where *k*_*X* → *XX*_ is the transition propensity from cell states X to cell state XX. Q is the quiescent state, AR_A_ is the anti-regenerative activation state, PR_A_ is the pro-regenerative activation state, and AR_R_ and PR_R_ are the anti-regenerative replicating state and the pro-regenerative replicating state, respectively.

Once activated, HSCs produce pro-regenerative or anti-regenerative molecules depending on the activation state.

29 $$ \frac{d}{dt}\left[ HGF\right]={k}_{HGF}\left(\frac{k_{HGF}^{nom}+\left[ TGF\beta \right]}{\left[ TGF\beta \right]}\right)P{R}_A-{k}_{up}\left[ HGF\right]\left[ ECM\right]-{\kappa}_{HGF}\left[ HGF\right]+{K}_{SS}^{HGF} $$

30 $$ \frac{d}{dt}\left[ ECM\right]={k}_{ECM}A{R}_A-{\kappa}_{deg}\left[ TNF\right]\left[ ECM\right]-{\kappa}_{ECM}(ECM)+{K}_{SS}^{ECM} $$

Each protein is produced at a phenotypic rate *k*_*xx*_, where xx is the species of interest. Each protein is degraded according for first order kinetics at a rate κ_xx_ and produced or degraded at a steady-state rate of K_ss_^xx^. In addition, the production of [HGF] is slowed by [TGFβ] such that when [TGFβ] is close to its initial value of 1, [HGF] is produced at a nominal rate according to *k*_*TNF*_^*Nom*^. Furthermore, [HGF] can be sequestered in the ECM with an uptake rate of k_up_. This model does not allow explicitly for sequestered HGF to be re-released during regeneration.

### Parameter estimation

Where possible, parameters related to molecules or cells found in the original model by Cook, Ogunnaike, and Vadigepalli [23] were tuned using a gain-matching technique. Parameters were selected so that the production of molecules included in the original model occurred with approximately the same dynamics during the first 10 h post-PHx in both models and followed previously-studied dynamics (Additional file 1: Figure S14).

All other parameters were estimated using order of magnitude estimates so that the parameters were of the same magnitude as the parameters above. These remaining parameters were tuned manually to match experimentally observed molecular profiles or physiological observations during liver regeneration qualitatively. A complete table of model parameter values used and physiological interpretations can be found in Additional file 1: Tables S1-S3. While the dimensionality of the single-cell HSC data generated in this work is large, the number of parameters that are constrained by these new data are relatively fewer, as the experimental results are informative primarily of the balance of HSC functional states. In contrast to HSC-related parameter values, other parameter values were largely unconstrained due to the relatively large number of parameters compared to the limited experimental data. Put more clearly, the amount of data available for parameter fitting is relatively low and is not sufficient for estimating the large number of parameters included in our model precisely. We sought to quantify how these imprecise parameter estimates influence the model outcome beyond what is typically included in sensitivity analyses by including in Additional file 1: Table S1-S3 two columns that indicate the model outcome (recovery fraction) for parameter values 1/2× to 2× of nominal value, and for parameter values 1/10× to 10× nominal value. Although many parameters are unconstrained by experiments, parameters with large effects on the regeneration outcome are constrained by the model structure and quantitative relationship to other parameters, etc., while parameters with small effects on regeneration outcome are more traditionally unconstrained (but also likely less important to identify experimentally). The parameter values presented in this work, therefore, should be interpreted in terms of their relationship to other parameters and relative influence on model behavior.

### Sensitivity analysis

Global sensitivity coefficients were estimated by sampling the model’s parameter space within 10% to 1000% of each parameter’s nominal value using a Latin hypercube sampling method to sample each parameter uniformly over three orders of magnitude. We then simulated liver regeneration following 70% PHx using 150 parameter sets for the sensitivity analyses including k_ap_ and 1500 parameter sets for the sensitivity analyses excluding k_ap_. We calculated the overall mass recovery (*N*_*i*_) for each case of liver regeneration and the time to liver failure (*t*_*i*_) for each case of failed regeneration. We then calculated regeneration sensitivity coefficients and failure sensitivity coefficients for each parameter according to the partial rank correlation coefficient (PRCC) formulation using the “partialcorr” function in Matlab.

## Additional files

Additional file 1: Supplemental figures S1-S13 and Supplemental Tables S1-S4. (DOCX 2680 kb)

Additional file 2: Collated single cell mRNA expression data used in this study. (TXT 111 kb)

Additional file 3: R Code used to analyze the single cell data used in this study. (R 20 kb)

Additional file 4: Matlab code containing the mathematical model used in this study. (M 19 kb)

Additional file 5: Zip file containing the raw mRNA expression data generated from single cells used in this manuscript. Data were manually inspected for reactions with CT values above those for water (below the limit of detection). These reactions were manually failed resulting in their removal from the data sets. Processed and unprocessed data are included. (ZIP 2160 kb)
