# Supplementary material for: Cellular network modeling and single cell gene expression analysis reveals novel hepatic stellate cell phenotypes controlling liver regeneration dynamics
Source: BMC Syst Biol. 2018 Oct 3;12:86. doi: 10.1186/s12918-018-0605-7 (PMC6171157; doi:10.1186/s12918-018-0605-7)
Supplement: Supplementary file 1 — Supplemental figures S1-S13 and Supplemental Tables S1-S4. (DOCX 2680 kb) [file 12918_2018_605_MOESM1_ESM.docx]

**SUPPLEMENTAL FIGURE LEGENDS AND SUPPLEMENTAL TABLES**


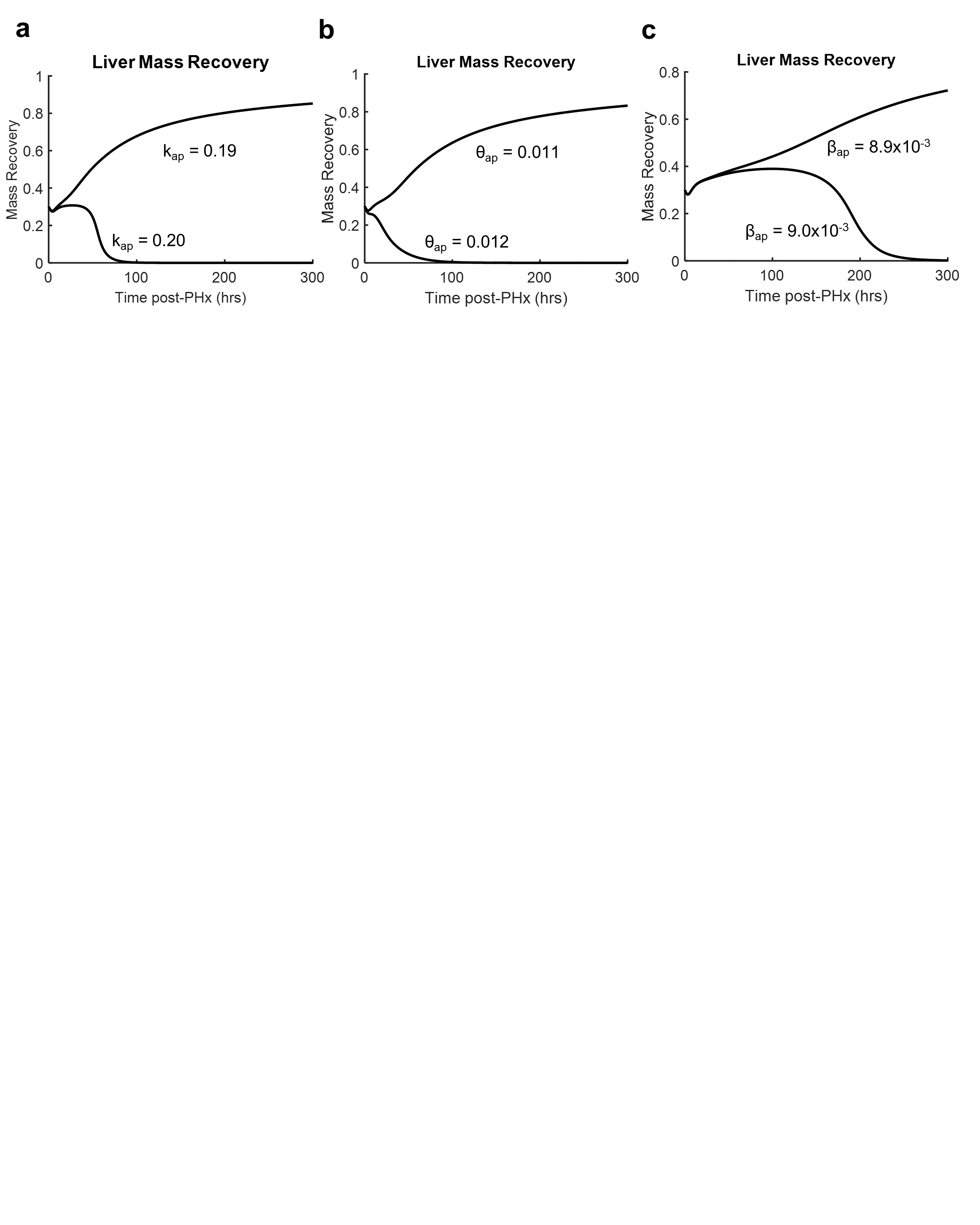
**Figure S1** Several parameters related to hepatocyte apoptosis cause a separatrix-like behavior between liver regeneration and liver failure. (a) Apoptosis rate, (b) shape parameter, (c) scale parameter.


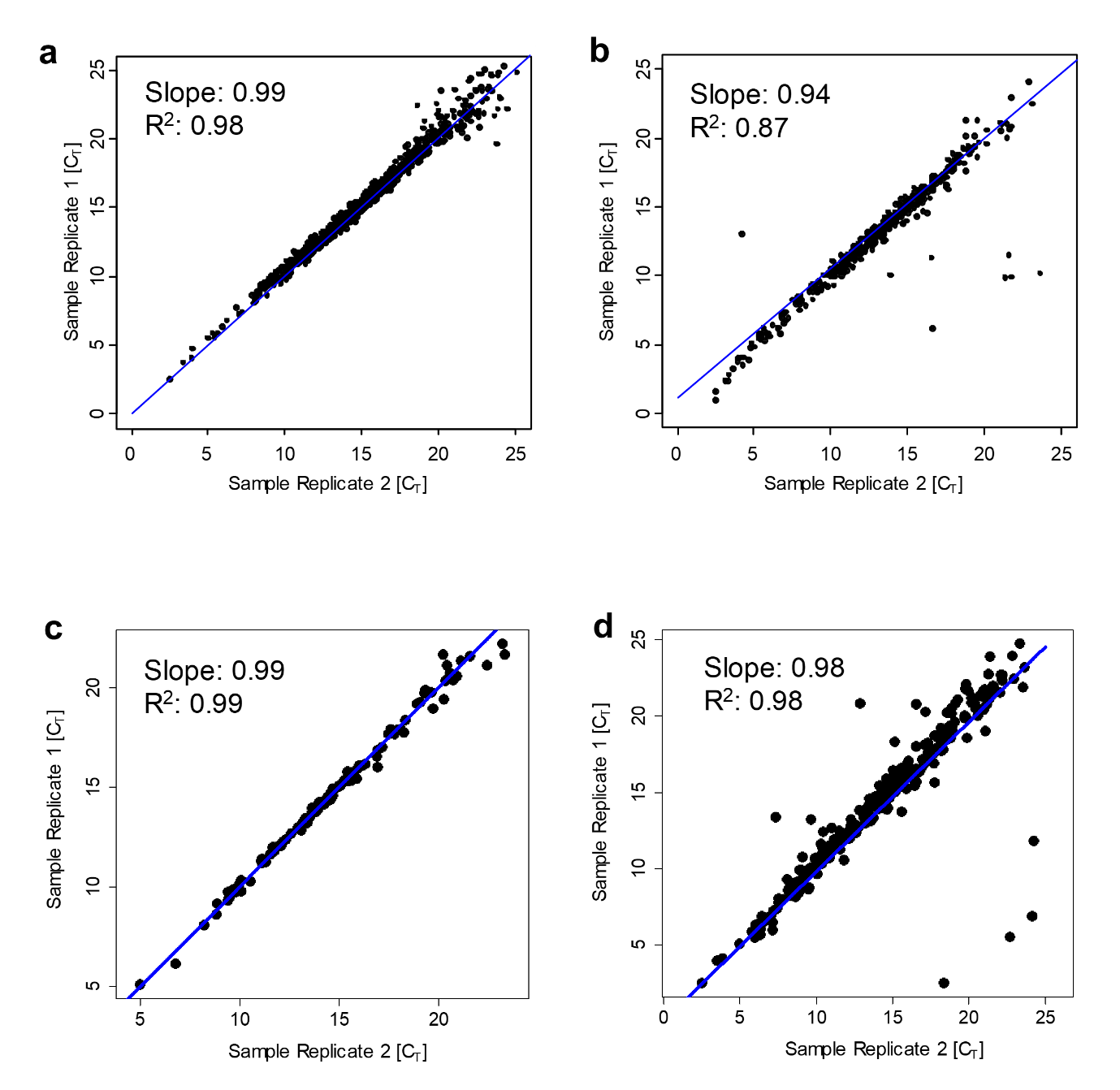


**Figure S2** Technical reproducibility of the experimental strategy within a Biomark array and across Biomark arrays. (a) One sample run twice on the same array. (b) One sample run twice on the same array. (c) One sample run on two different arrays. (d) One sample run on two different arrays.


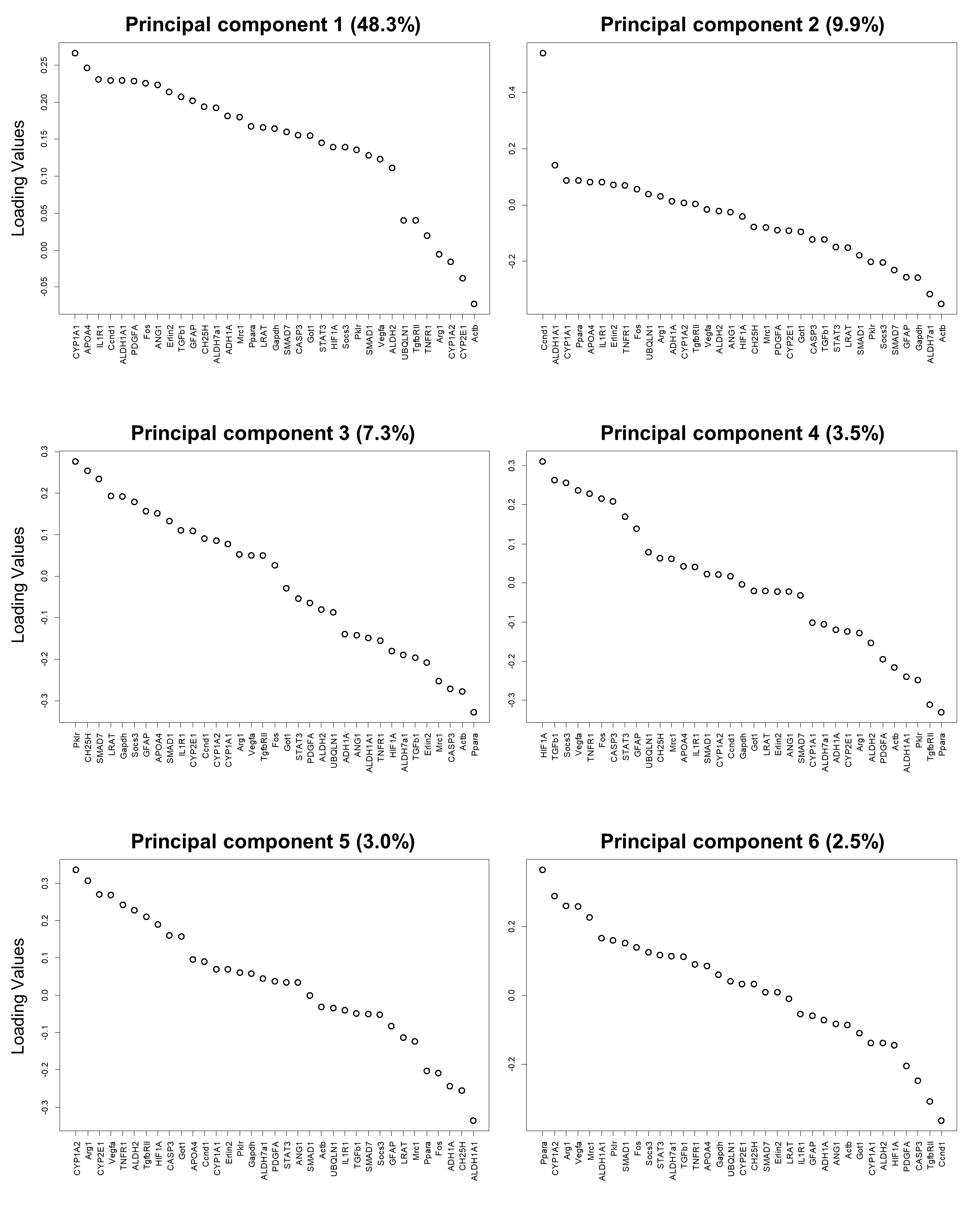


**Figure S3** Principal Component scores for PCs 1-6, used to discriminate between HSCs and hepatocytes.


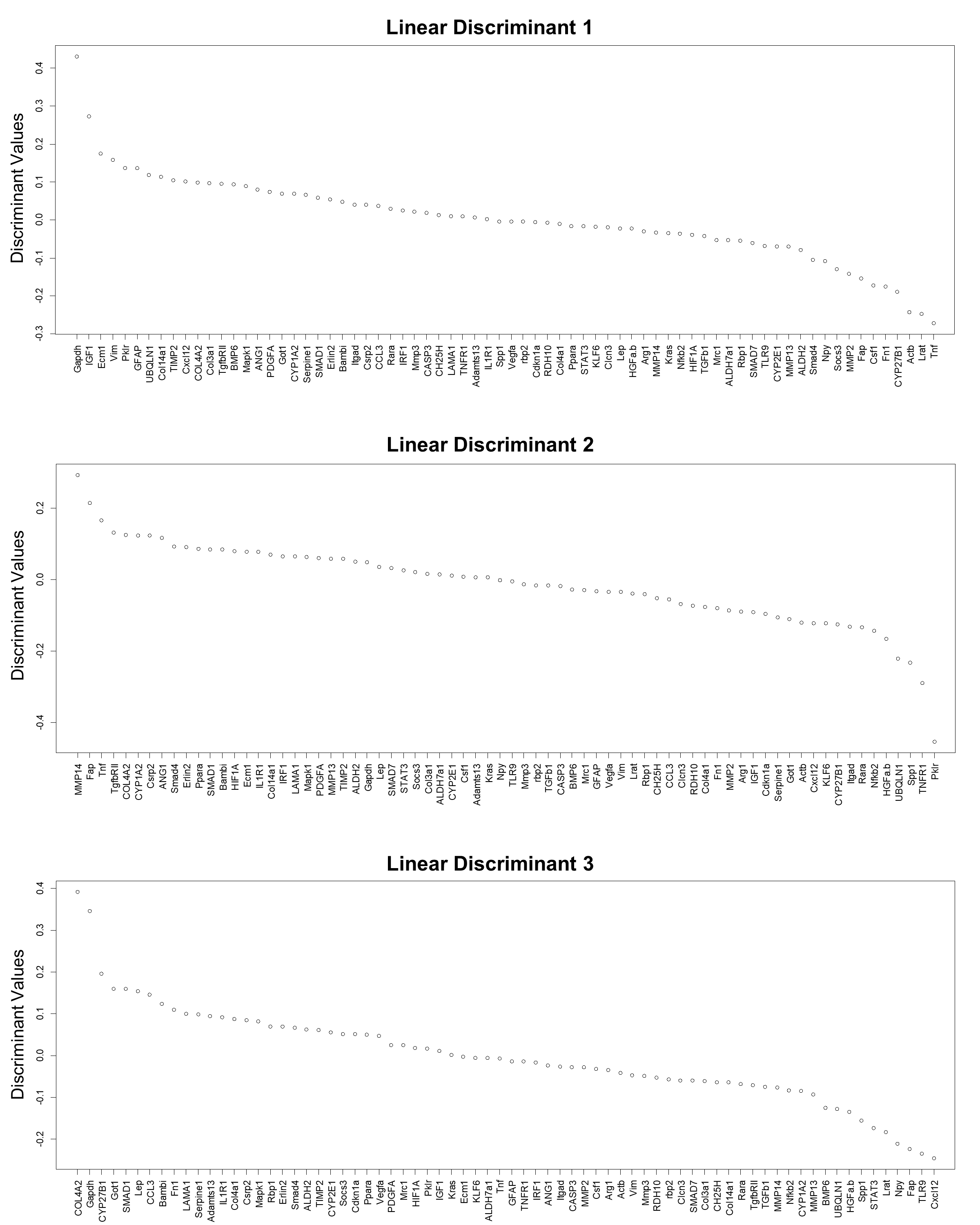


**Figure S4** Linear discriminants for LD1, LD2, LD3, used to discriminate among HSC functional states


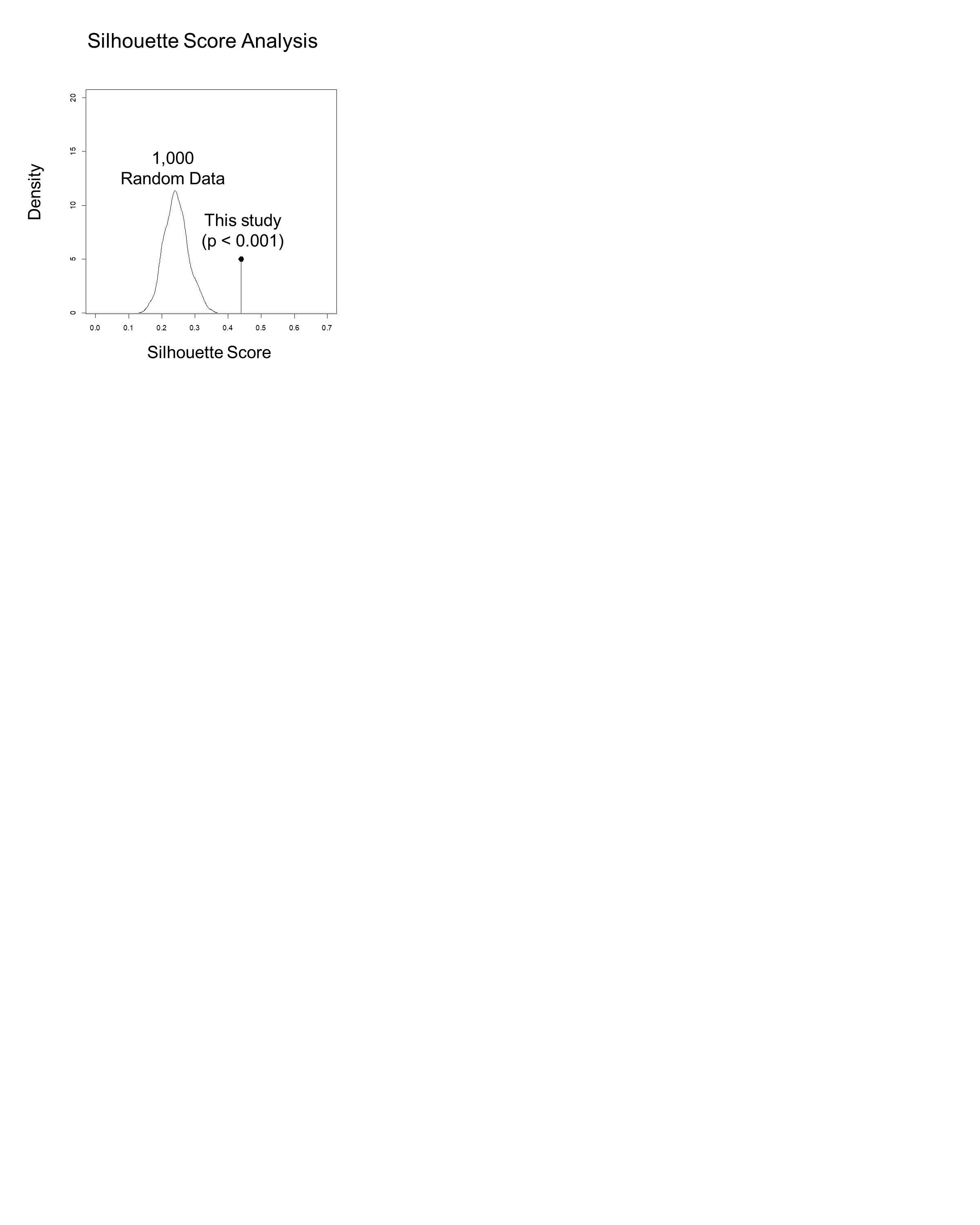


**Figure S5** Significance of clustering results assessed by silhouette score. Our silhouette score falls well outside the range generated from randomizing our data 1,000 times. This gives an empirical p-value of less than 0.001.


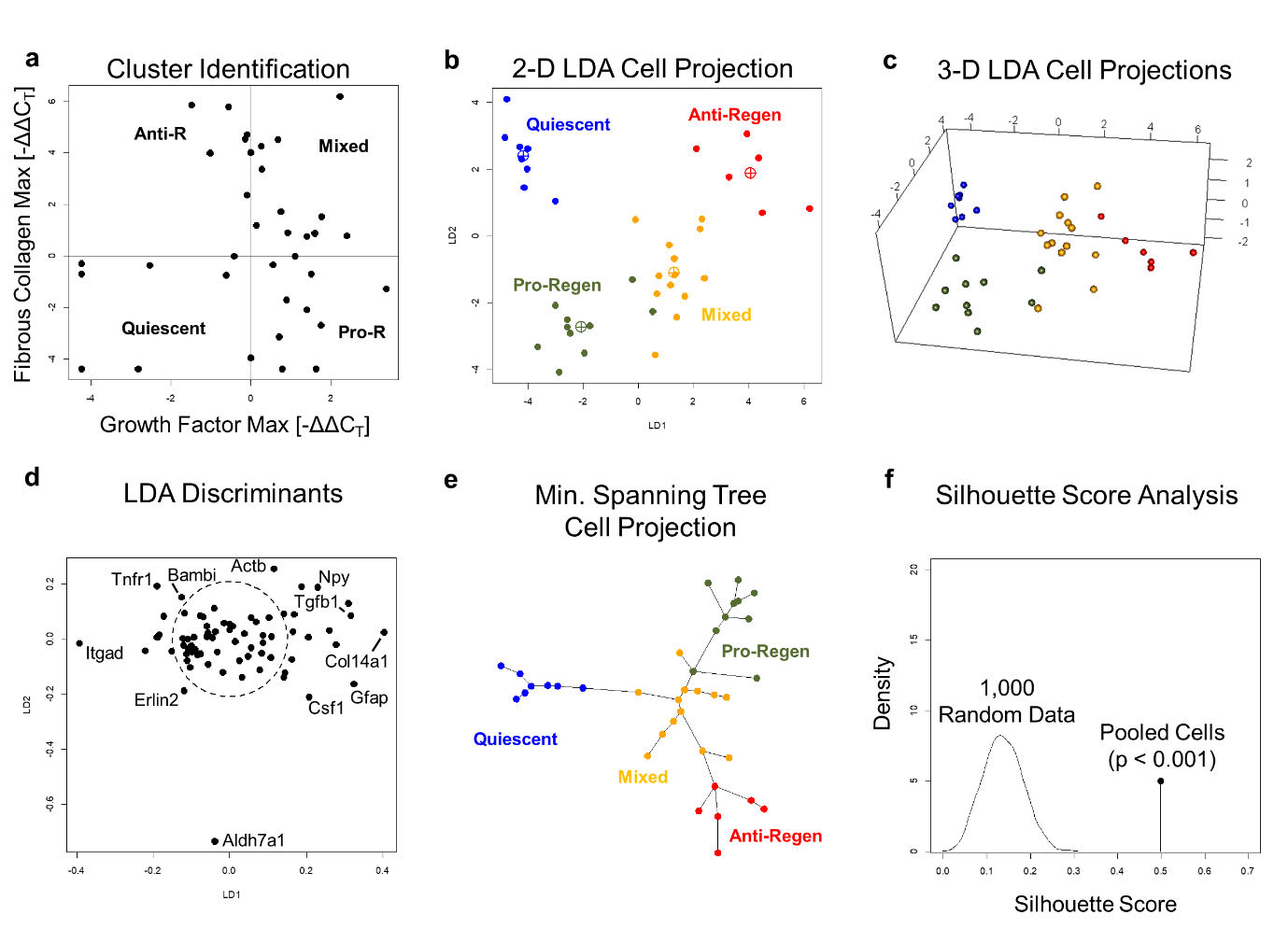


**Figure S6** Validation of single cell results using 10 cell pools of HSCs collected from the same animals. Pools of HSCs showed a stronger gene expression signal, but a higher variability than single cells. (a) Manual clustering of functional HSC states into four states: Quiescent (low GF/low collagen), Pro-regenerative (high GF/low collagen), Anti-regenerative (low GF/high collagen), and mixed (high GF/high collagen). (b) Linear discriminant analysis shows separation of the four HSC states in two dimensions. (c) LDA shows further separation of the four HSC states in three dimensions. (d) Genes contributing to discrimination among functional states. (e) Minimum spanning tree representation of single HSCs shows relationships between individual cells based on gene expression. (f) Significance of clustering results assessed by silhouette score.


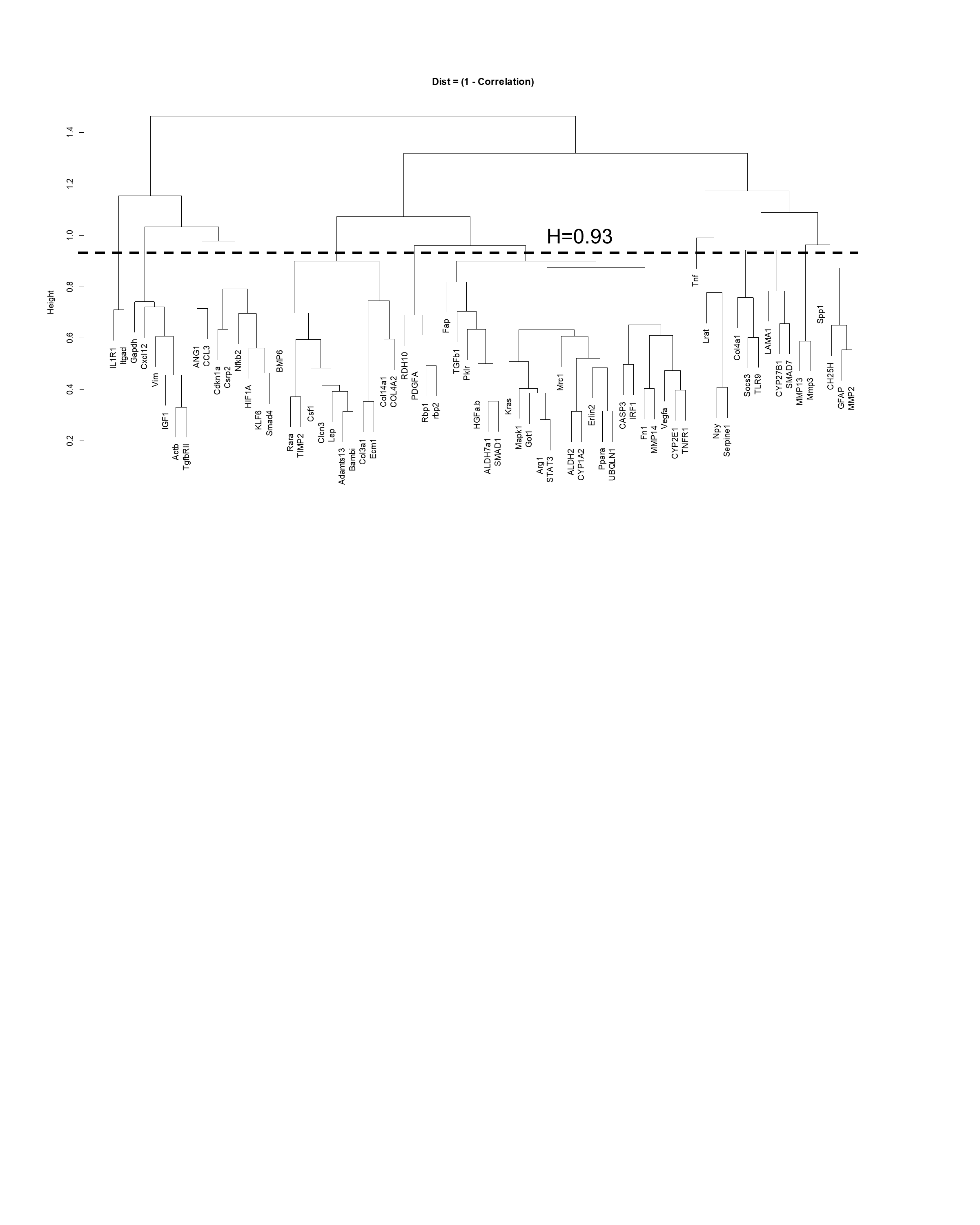


**Figure S7** Dendogram showing the definition of gene clusters using a height cutoff value of < 0.93.


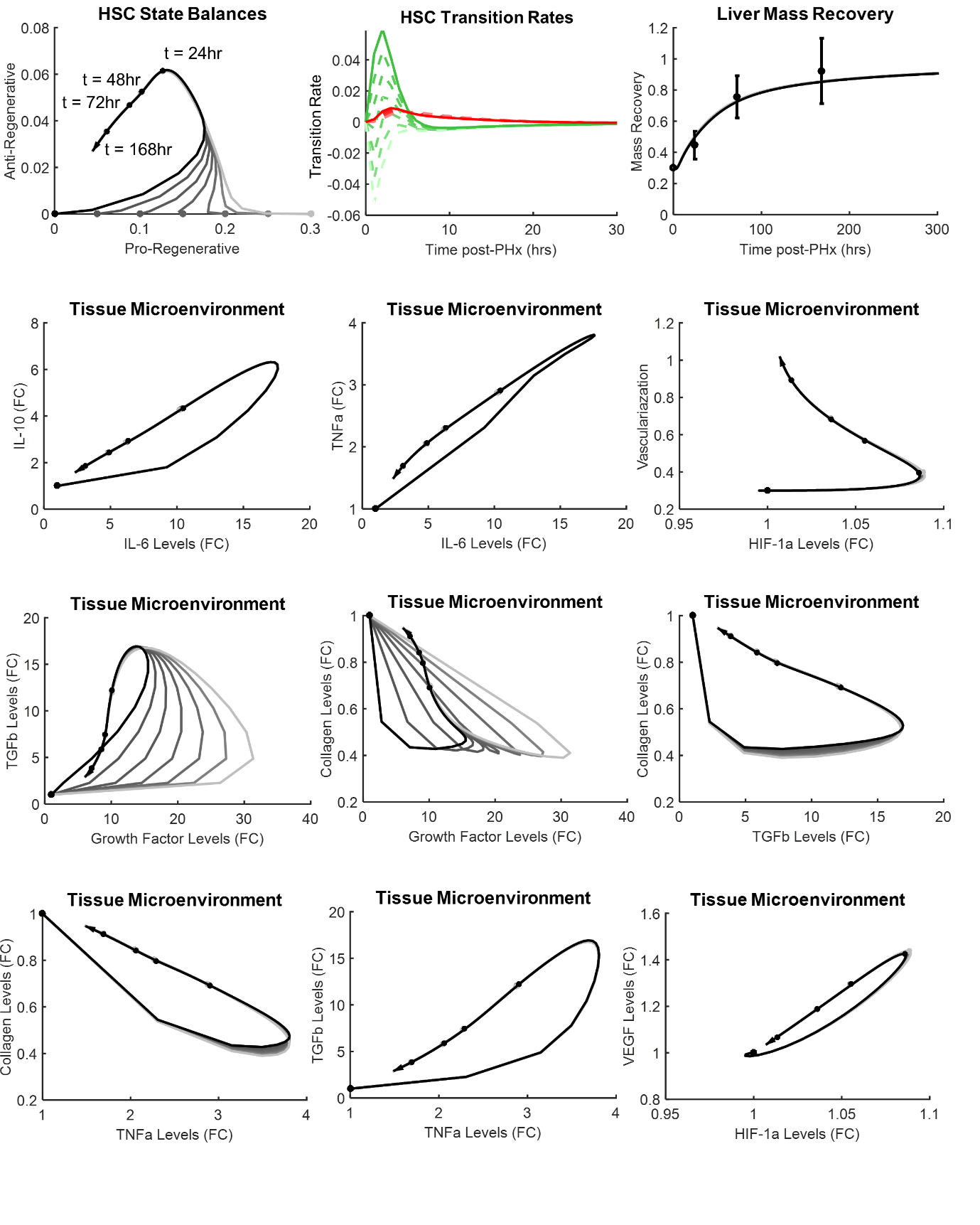


**Figure S8** Effects of increasing initial fraction of pro-regen HSC on dynamic tissue microenvironment


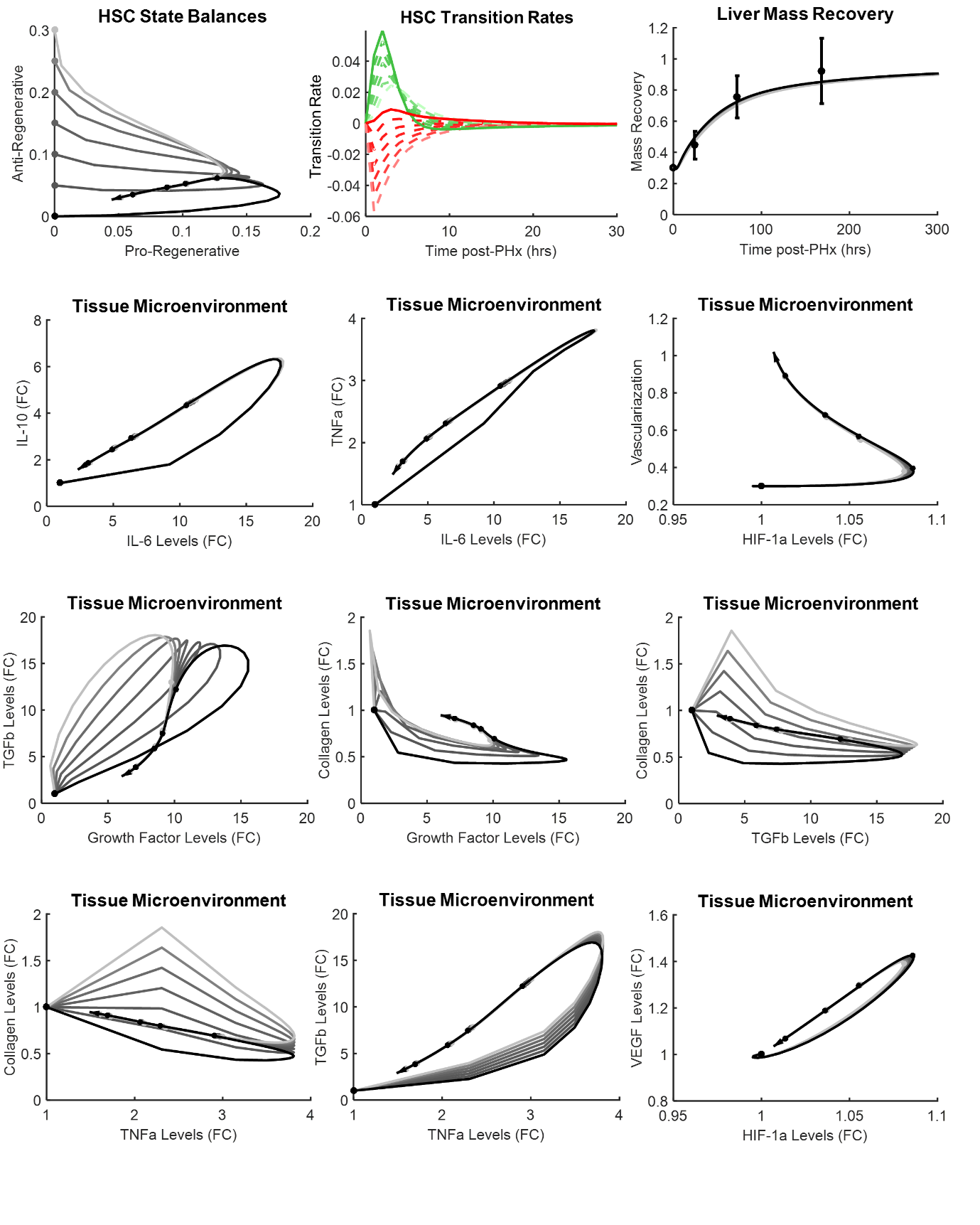


**Figure S9** Effects of increasing initial fraction of anti-regen HSC on dynamic tissue microenvironment


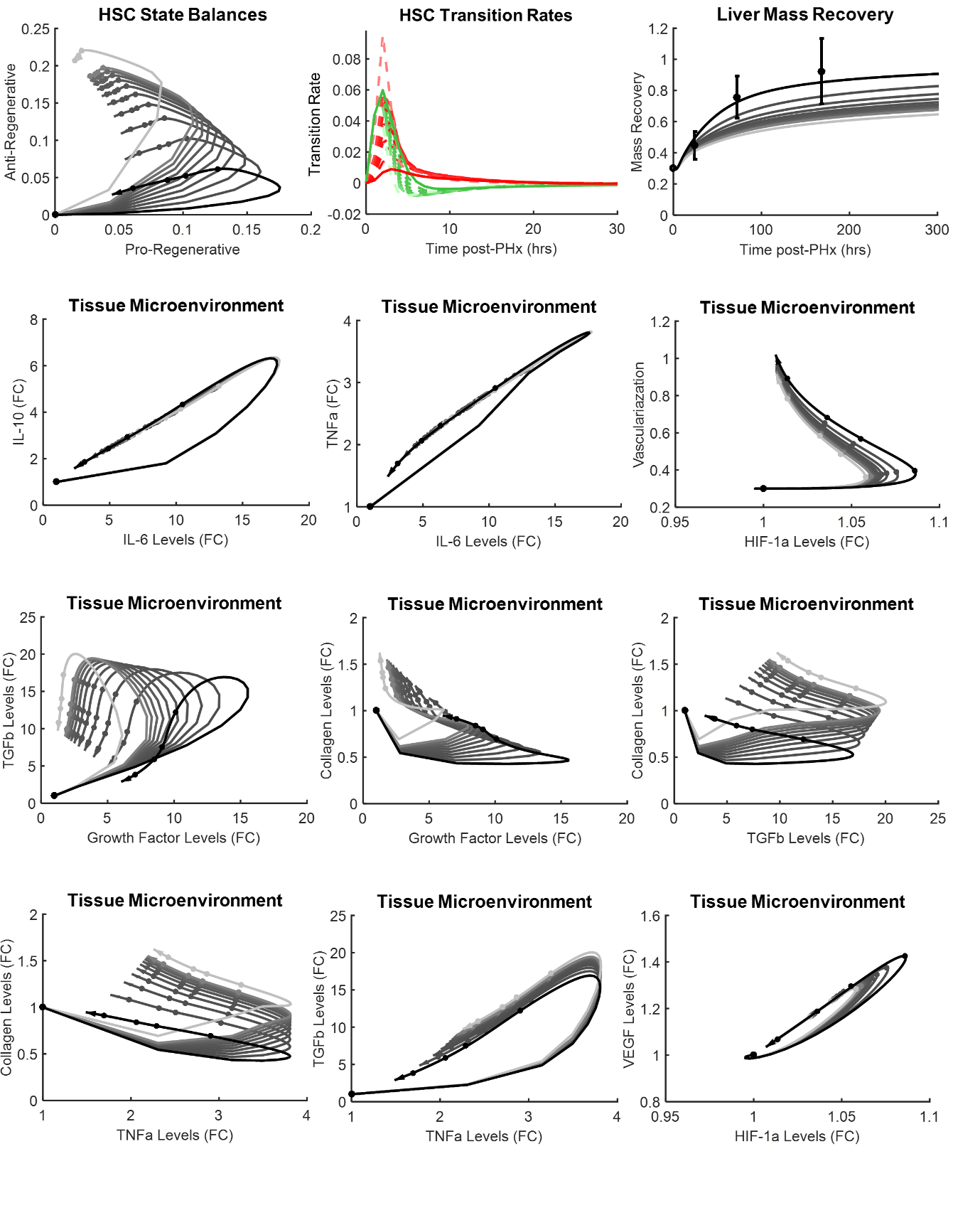


**Figure S10** Effects of increasing the anti-regen HSC transition rate on tissue microenvironment


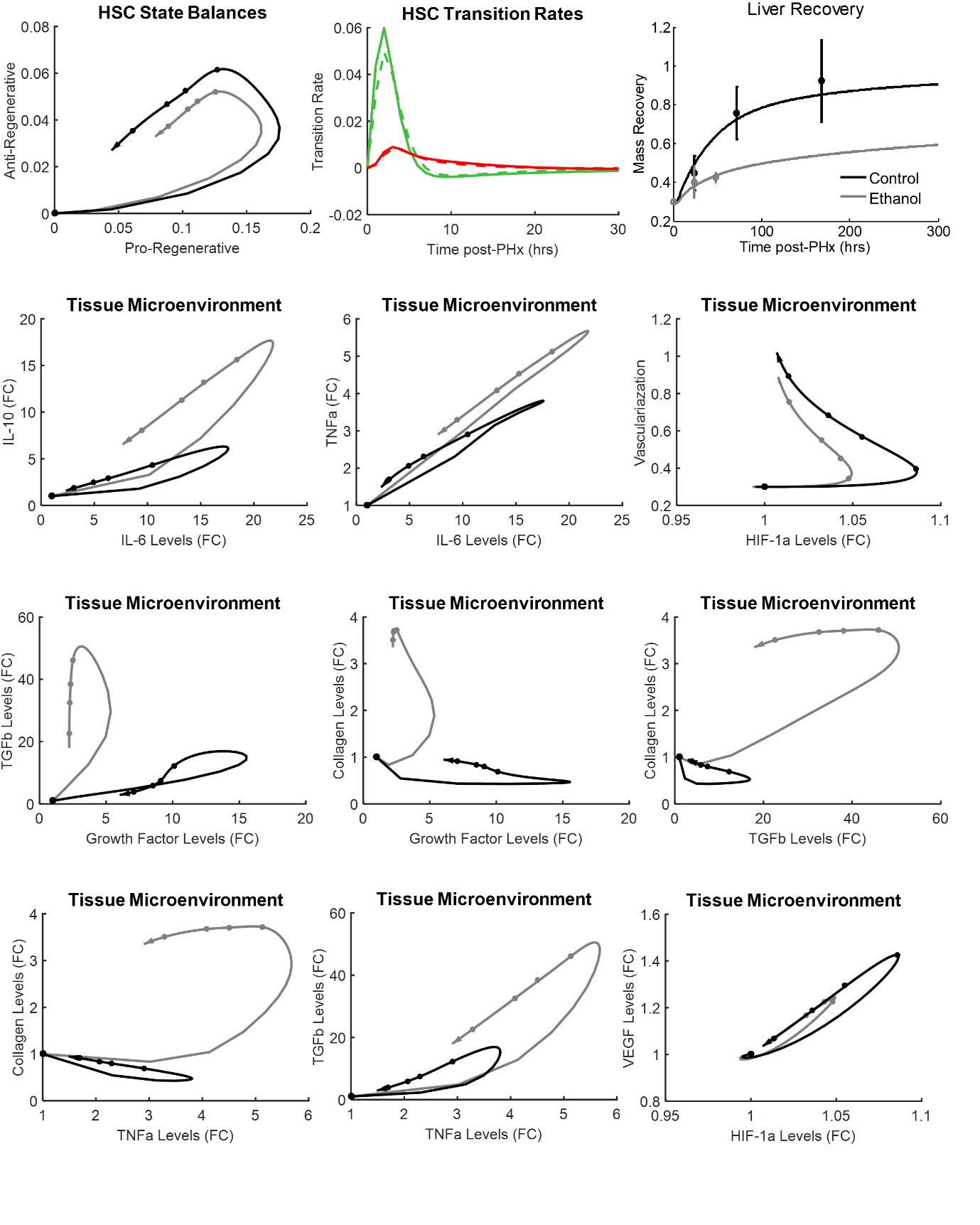


**Figure S11** Effects of chronic ethanol consumption on liver regeneration control and tissue microenvironment


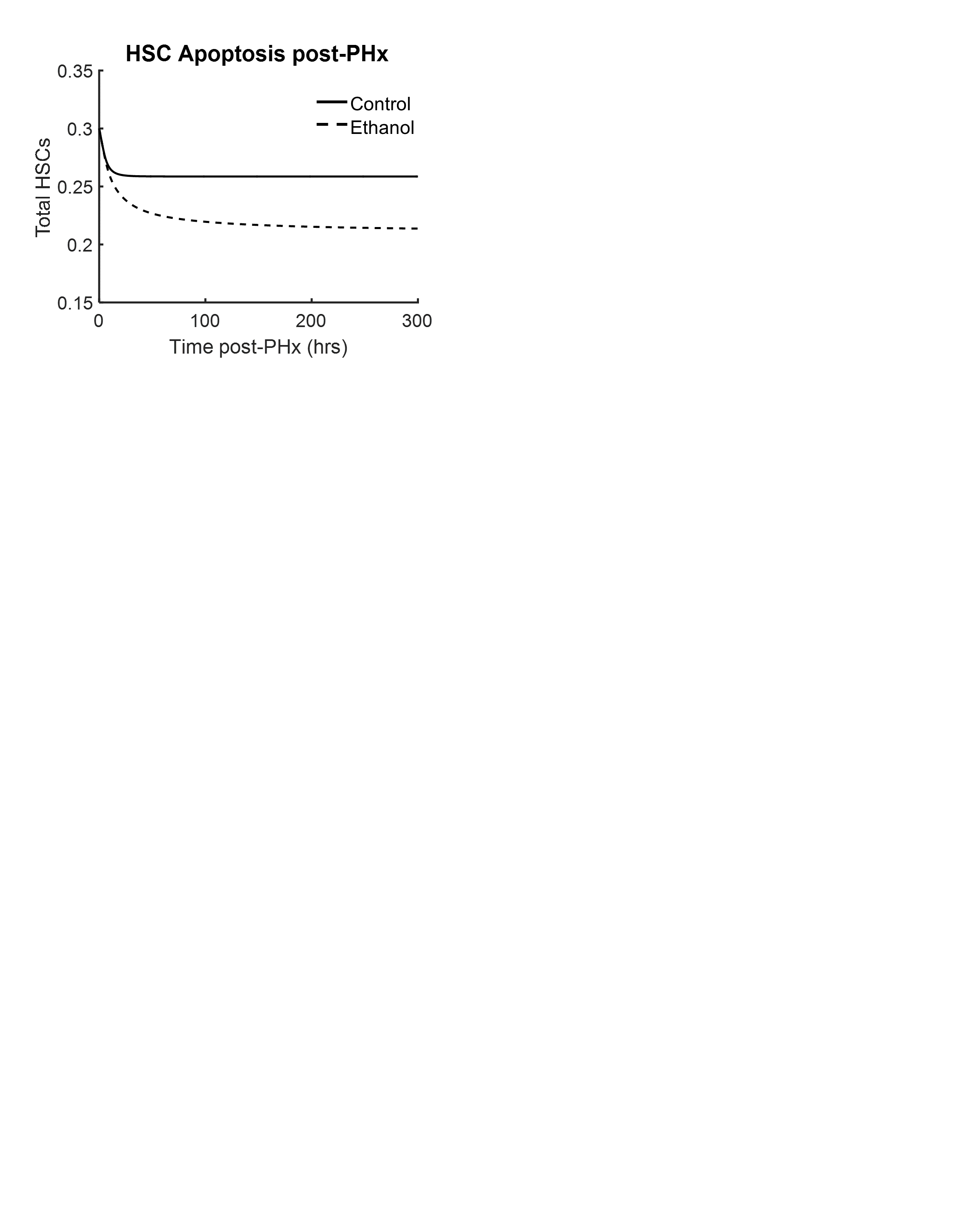


**Figure S12** Simulations predict that there is more apoptosis of HSCs in the ethanol-fed rats than controls


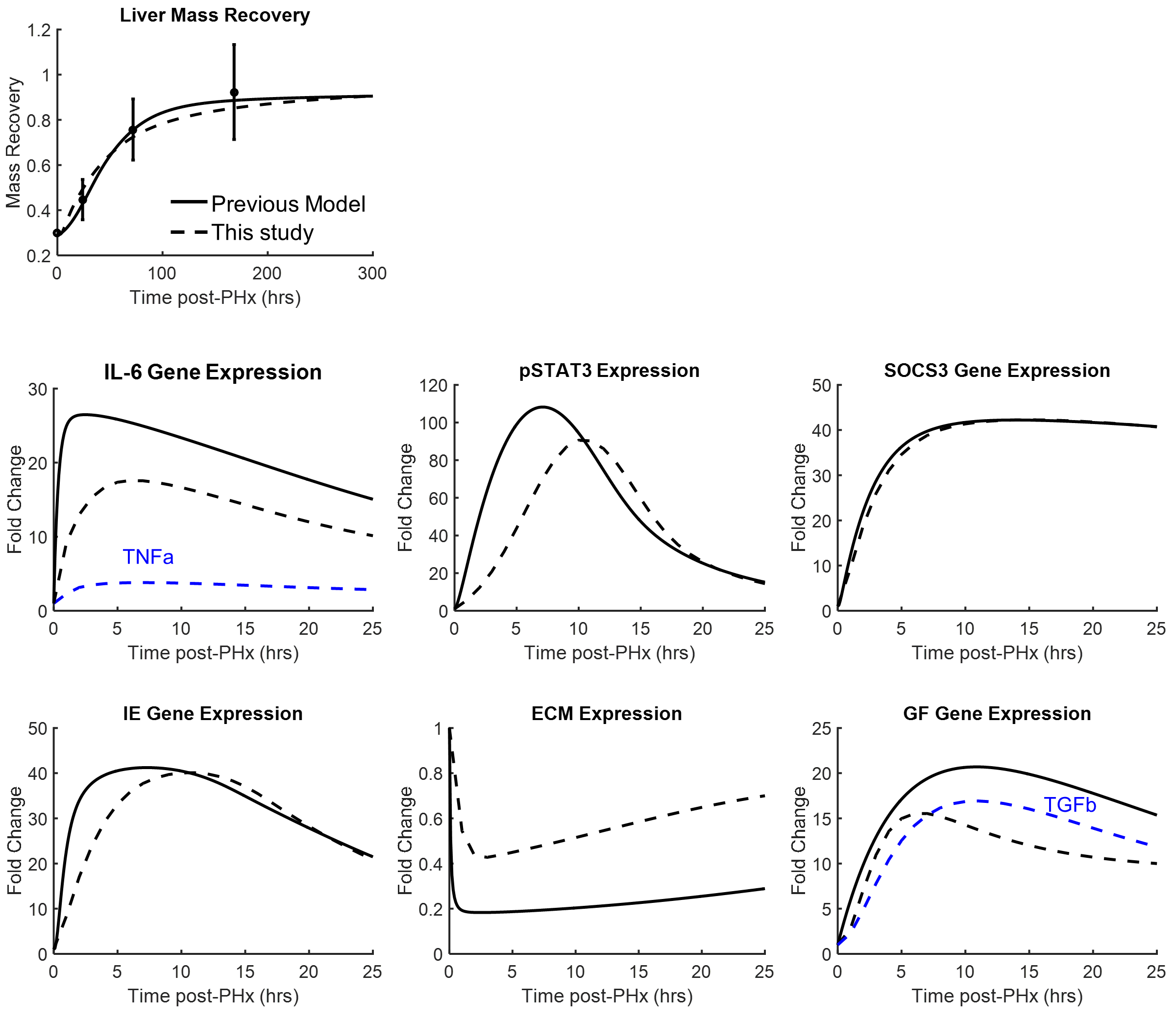


**Figure S13** Comparison of model dynamics between this model and our previous model during the first 25 hours post-resection [23, 36] (References refer to main text).

**Table S1:** Model parameter values for hepatocytes and physiological interpretations

| **Hepatocyte Parameters** | | |  |  |
| --- | --- | --- | --- | --- |
| **Parameter** | **Value** | **Physiological Interpretation** | **Effect of Parameter change on regeneration fraction (1/2 – 2x nominal)** | **Effect of Parameter change on regeneration fraction (1/10 – 10x nominal)** |
| M^hepatocyte^ | Species-specific 23.21 in rats | Metabolic demand | 0.89-0.0 | 0.9-0.0 |
| k_growth_ | Species-specific 8.29 x 10^-4^ in rats | Hepatocyte growth rate | 0.9-0.89 | 0-0.9 |
| k_IL6_^Hepatocyte^ | 0.3 | IL-6 production rate by hepatocytes | 0.87-0.92 | 0.84-0.98 |
| V_JAK_ | 6x10^4^ | JAK activation rate | 0.94-0.83 | 0.94-0.69 |
| K_m_^JAK^ | 10^4^ | JAK Michaelis constant | 0.83-0.94 | 0.69-0.94 |
| κ_JAK_ | 0.4 | JAK degradation rate | 0.94-0.82 | 0.98-0.0 |
| [STAT] | 2 | Concentration of monomeric STAT3 | 0.85-0.92 | 0.0-0.99 |
| V_STAT_ | 7.5x10^2^ | STAT3 activation rate | 0.88-0.9 | 0.8-0.91 |
| K_m_^STAT^ | 0.4 | STAT3 Michaelis constant | 0.91-0.87 | 0.95-0.79 |
| κ_STAT_ | 0.1 | STAT3 degradation rate | 0.89-0.89 | 0.89-0.89 |
| V_SOCS_ | 2.4x10^4^ | SOCS3 activation rate | 0.94-0.82 | 0.98-0.0 |
| K_m_^SOCS^ | 7x10^-4^ | SOCS3 Michaelis constant | 0.94-0.82 | 0.98-0.0 |
| κ_SOCS_ | 0.4 | SOCS3 degradation rate | 0.83-0.94 | 0.6-0.98 |
| K_I_^SOCS^ | 1.5x10^-2^ | SOCS3 inhibition of STAT3 signaling | 0.87-0.91 | 0.79-0.95 |
| V_IE_ | 2.5x10^2^ | Immediate early (IE) gene expression rate | 0.85-0.93 | 0.72-0.97 |
| K_m_^IE^ | 18 | IE gene Michaelis constant | 0.92-0.86 | 0.95-0.77 |
| κ_IE_ | 5 | IE gene degradation | 0.93-0.84 | 0.98-0.67 |
| k_Q→P_ | 7x10^-3^ | Hepatocyte priming rate | 0.84-0.93 | 0.67-0.98 |
| k_P→R_ | 4.4x10^-3^ | Hepatocyte replication transition rate | 0.85-0.93 | 0.75-0.98 |
| k_R→Q_ | 5.4x10^-2^ | Hepatocyte requiescence of replicating cells | 1.2-0.81 | 4.03-0.69 |
| k_prol_ | 2x10^-2^ | Hepatocyte proliferation rate | 0.8-1.24 | 0.7 – (>2.5) |
| k_req_ | 0.1 | Hepatocyte requiescence of primed cells | 0.92-0.86 | 0.96-0.77 |
| θ_req_ | 8 | Requiescence shape parameter | 0.93-0.86 | 0.96-0.86 |
| β_req_ | 3 | Requiescence scale parameter | 0.89-0.9 | 0.88-0.9 |
| k_ap_ | 0.1 | Hepatocyte apoptosis rate | 0.9-0.87 | 0.9-0.0 |
| θ_ap_ | 9x10^-3^ | Apoptosis shape parameter | 0.9-0.0 | 0.9-0.0 |
| β_ap_ | 4.5x10^-3^ | Apoptosis scale parameter | 0.92-0.0 | 0.92-0.0 |
| k_vas_ | 2x10^-2^ | Vascularization rate | 0.91-0.9 | 0.93-0.9 |

**Table S2:** Model parameter values for Kupffer cells and physiological interpretations

| **Kupffer cell parameters** | | |  |  |
| --- | --- | --- | --- | --- |
| **Parameter** | **Value** | **Physiological Interpretation** | **Effect of Parameter change on regeneration fraction (1/2–2x nominal)** | **Effect of Parameter change on regeneration fraction (1/10–10x nominal)** |
| M^KC^ | Species-specific 23.21 in rats | Metabolic demand | 0.83-0.87 | 0.72-0.7 |
| HL | 2 | Hypoxia load | 0.89-0.89 | 0.89-0.89 |
| k_TNF_ | 16 | TNFa production rate | 0.83-0.97 | 0.74-1.02 |
| κ_TNF_ | 2 | TNFa degradation rate | 0.97-0.83 | 1.02-0.74 |
| k_IL6_^KC^ | 180 | IL-6 production rate by Kupffer cells | 0.77-0.97 | 0.54-1.0 |
| κ_IL6_ | 0.9 | IL-6 degradation rate | 0.91-0.87 | 0.92-0.67 |
| k_IL10_ | 20 | IL-10 production rate | 0.91-0.87 | 0.93-0.83 |
| κ_IL10_ | 0.9 | IL-10 degradation rate | 0.87-0.91 | 0.83-0.94 |
| K_I_^IL10^ | 3 | IL-10 inhibition of TNFa production | 0.94-0.79 | 0.97-0.62 |
| k_TGF_^KC^ | 30 | TGF-β production rate by Kupffer cells | 0.89-0.89 | 0.89-0.89 |
| k_PDGF_ | 15 | PDGF production rate | 0.89-0.89 | 0.89-0.89 |
| κ_PDGF_ | 0.9 | PDGF degradation rate | 0.89-0.89 | 0.89-0.89 |
| k_Q→A_^KC^ | 7x10^-3^ | Kupffer cell activation rate | 0.82-0.96 | 0.71-1.0 |
| k_A→R_^KC^ | 2.2x10^-2^ | Kupffer cell replication transition rate | 0.89-0.9 | 0.88-0.95 |
| k_R→Q_^KC^ | 5.4x10^-2^ | Kupffer cell requiescence of replicating cells | 0.92-0.89 | 1.04-0.88 |
| k_prol_^KC^ | 2x10^-2^ | Kupffer cell proliferation rate | 0.89-0.93 | 0.88-1.04 |
| k_req_^KC^ | 0.3 | Kupffer cell requiescence of primed cells | 0.96-0.82 | 1.01-0.72 |
| θ_req_^KC^ | 8 | Kupffer cell requiescence shape parameter | 0.95-0.88 | 1.12-0.88 |
| β_req_^KC^ | 3 | Kupffer cell requiescence scale parameter | 0.89-0.91 | 0.89-0.94 |
| k_ap_^KC^ | 0.1 | Kupffer cell apoptosis rate | 0.89-0.89 | 0.89-0.87 |
| θ_ap_^KC^ | 9x10^-3^ | Kupffer cell apoptosis shape parameter | 0.89-0.82 | 0.9-0.69 |
| β_ap_^KC^ | 4.5x10^-3^ | Kupffer cell apoptosis scale parameter | 0.9-0.87 | 0.9-0.7 |

**Table S3:** Model parameter values for hepatic stellate cells and physiological interpretations

| **Hepatic stellate cell parameters** | | |  |  |
| --- | --- | --- | --- | --- |
| **Parameter** | **Value** | **Physiological Interpretation** | **Effect of Parameter change on regeneration fraction (1/2–2x nominal)** | **Effect of Parameter change on regeneration fraction (1/10–10x nominal)** |
| M^HSC^ | Species-specific 23.21 in rats | Metabolic demand | 0.89-0.87 | 0.89-0.79 |
| k_HGF_ | 25 | HGF production rate | 0.81-0.95 | 0.69-0.99 |
| κ_HGF_ | 0.23 | HGF degradation rate | 0.91-0.86 | 0.92-0.75 |
| k_up_ | 0.6 | HGF uptake by ECM | 0.94-0.83 | 0.97-0.71 |
| k_TGF_^HSC^ | 8 | TGF-β production rate by hepatic stellate cells | 0.91-0.85 | 0.92-0.68 |
| κ_TGF_ | 0.9 | TGF-β degradation rate | 0.76-0.95 | 0.63-0.97 |
| K_I_^TGF^ | 5 | TGF-β inhibition of HGF production | 0.93-0.82 | 0.95-0.61 |
| k_ECM_ | 100 | ECM production rate | 0.82-0.93 | 0.67-0.96 |
| k_degrad_ | 7 | ECM degradation rate by MMPs | 0.89-0.89 | 0.9-0.88 |
| κ_ECM_ | 3 | Constitutive ECM degradation rate | 0.81-0.95 | 0.66-0.99 |
| k_Q→PR_ | 7x10^-2^ | State transition rate: Quiescent to Pro-regenerative | 0.94-0.79 | 0.96-0.62 |
| k_Q→AR_ | 3x10^-2^ | State transition rate: Quiescent to Anti-regenerative | 0.89-0.89 | 0.89-0.89 |
| k_PR→PRR_ | 4.4x10^-3^ | State transition rate: Pro-regenerative to Replicating | 0.89-0.89 | 0.89-0.89 |
| k_AR→ARR_ | 4.4x10^-3^ | State transition rate: Anti-regenerative to Replicating | 0.89-0.89 | 0.89-0.89 |
| k_PRR→PR_ | 5.4x10^-2^ | State transition rate: Replicating to Pro-regenerative | 0.89-0.89 | 0.89-0.89 |
| k_ARR→AR_ | 5.4x10^-2^ | State transition rate: Replicating to Anti-regenerative | 0.89-0.89 | 0.89-0.9 |
| k_prol_ | 8.5x10^-3^ | Hepatic stellate cell proliferation rate | 0.89-0.88 | 0.88-0.84 |
| k_req_ | 0.2 | Requiescence rate of pro- and anti-regenerative cells | 0.89-0.89 | 0.89-0.89 |
| θ_req_^HSC^ | 8 | Hepatic stellate cell requiescence shape parameter | 0.89-0.89 | 0.89-0.89 |
| β_req_^HSC^ | 3 | Hepatic stellate cell requiescence scale parameter | 0.89-0.89 | 0.89-0.89 |
| k_ap_^HSC^ | 0.1 | Hepatic stellate cell apoptosis rate | 0.89-0.88 | 0.89-0.78 |
| θ_ap_^HSC^ | 9x10^-3^ | Hepatic stellate cell apoptosis shape parameter | 0.89-0.89 | 0.89-0.82 |
| β_ap_^HSC^ | 4.5x10^-3^ | Hepatic stellate cell apoptosis scale parameter | 0.85-0.94 | 0.81-0.99 |

**Table S4:** Primer sequences used in the single-cell gene expression experiments

| **Gene** | **RefSeq ID** | **Forward Sequence** |  | **Reverse Sequence** |
| --- | --- | --- | --- | --- |
| Actb | NM_031144 | AAGGCCAACCGTGAAAAGAT |  | ACCAGAGGCATACAGGGACA |
| Adamts1 | NM_024400 | GGACAGGTGCAAGCTTACCT |  | CACAGCCAGCTTTCACACAC |
| Adamts13 | XM_006233879 | ACCCTCTCAGGAGGCTAAAT |  | GTGACTGGGATTCTGGTTAGTG |
| Adh1a | NM_019286 | GATGCCGACTTGGACATTGC |  | TGGCTCGCTCAACACTCTTT |
| Alb | NM_134326 | TGGCACAATGAAGTGGGTAA |  | GGGCGATCTCACTCTTGTGT |
| Aldh1a1 | NM_022407 | GCCATCACTGTGTCTTCTGC |  | CATCTTGAATCCACCGAAGG |
| Aldh2 | NM_032416 | TTACCTGTCCCAAGCTCTGC |  | GCACGCCACTTTACGAGTTC |
| Aldh7a1 | NM_001271105 | GGAATCATCACTGCCTTCAAC |  | AGTTGTTGGTGCTCCTTTCC |
| Ang1 | NM_001006992 | CGTCCTCTGTTGTCGGTTTT |  | CGTGTACCTGGGGTCGTC |
| Apoa4 | NM_012737 | AGCCCCTGGGGGATAAGT |  | CCAGCTGCTGCCTGAACT |
| Arg1 | NM_017134 | GGTAGAGAAAGGTCCCGCAG |  | CAGACCGTGGGTTCTTCACA |
| Bambi | NM_139082 | AGCAGAAACCTCATCACTAAGG |  | GCTGTAGTGCAAACGAGAGA |
| Bmp6 | NM_013107 | CAGCAACAATCGCAACAGAC |  | GGGAGTTGTAGAGATCCAGCA |
| Casp3 | NM_012922 | TCTACCGCACCCGGTTACTA |  | TGACTGGATGAACCATGACC |
| Ccl3 | NM_013025 | CGCCATATGGAGCTGACAC |  | GTGGAATTTGCCGTCCATAG |
| Ccnd1 | NM_171992 | TGTGATATGTACCAGCCACAGG |  | CGAACAGACGACGGCATACT |
| Cdkn1a | NM_080782 | GCGCCCTCCGTTTCTTACTT |  | TCGCAGACCTCTAGCATCCA |
| Ch25h | NM_001025415 | CTGCACTGGAACAGGGCTAA |  | ACTGCCCAGCAGGAACAAAT |
| Clcn3 | NM_053363 | GACTGTCTCTCTGGTGGTTATTG |  | GCCAGGGTTGTATGAGTGAA |
| Col14a1 | NM_001130548 | CAGGCCAGAGGGGATTTC |  | AATGCCAATTGGTCCAGGT |
| Col3a1 | NM_032085 | ATGTGGGACCTGGTTTCTTC |  | CAGTCTAGTGGCTCATCATCAC |
| Col4A1 | NM_001135009 | CCAGCGGTGGTTATGACTTC |  | GGCCACCATCTTGAGACTTC |
| Col4A2 | XM_001076134 | CTGTCAGCAAATGGGCACT |  | TTAGGAGGTGGGTGTTAGCAG |
| Csf1 | NM_023981 | CTGACTCTGGTAGGGAAGGATA |  | GAGACCAAGGAGCAAGTAAGAG |
| Csf2 | NM_053852 | CTAATGAGTTCTCCATCCAGAGG |  | CCCGTAGACCCTGCTTGTAT |
| Csf3 | NM_017104 | GGTTTTCCTGACCCCGTAGG |  | TAGGCCAGCAAGCGCTAAAA |
| Csrp2 | NM_177425 | ACATGGACCGTGGTGAGAG |  | GTAGGCCTGTGAGGTTGAGC |
| Cxcl1 | NM_030845 | ACTCAAGAATGGTCGCGAGG |  | ACGCCATCGGTGCAATCTAT |
| Cxcl12 | NM_001033882 | GGCCTCTGGGCACAGTTA |  | TGGTGGAAGGTTGCTACTCC |
| Cyp1a1 | NM_012540 | CTCCCTGGGGTCCTAGAGAACA |  | CTCTGTGGCTGATGTGAAGGC |
| Cyp1a2 | NM_012541 | GGAACACTATCAAGACTTCAACAAGA |  | AATCCAGCTCCAAAGATGTCA |
| Cyp27b1 | NM_053763 | GGCTCCTATGCCCACCTC |  | CACAGCCTTTAGCAGGGGTA |
| Cyp2b1 | NM_001134844 | CGGACCTTTTCCCTCCTAAG |  | GGAACCCAGAGAAGAACTCAAA |
| Cyp2e1 | NM_031543 | CTGACTGTCTCCTCATAGAGATGG |  | TCACAGAAACATTTTCCATTGTGT |
| Dcn | NM_024129 | CGGTGGCAAATACCCGGATTA |  | TCTGCTCAAATGGTCCAGCC |
| Ecm1 | NM_053882 | TGACCCGTGACCAGTTCTTAC |  | GGTGCTGCATAGCCTACTTC |
| Erlin2 | NM_001106088 | CCCAGAAACAGAAGGTGGTG |  | GCAACCTGTGCCACTTTTTC |
| Fap | NM_138850 | GAAGAGGAAATGCTTGCTACAAA |  | TGGTATGTCCGAATCATTAAATTC |
| Fn1 | NM_019143 | CAGCCCCTGATTGGAGTC |  | TGGGTGACACCTGAGTGAAC |
| Fos | NM_022197 | GGGACAGCCTTTCCTACTACC |  | GATCTGCGCAAAAGTCCTGT |
| Gapdh | NM_017008 | TGGCCTCCAAGGAGTAAGAA |  | GGCCTCTCTCTTGCTCTCAG |
| Gfap | NM_017009 | AAGATCCCGAGGCAAAGAAT |  | TCGTCAGGGTTCTTCCAGAT |
| Got1 | NM_012571 | ACGAATCACCTGGTCCAATC |  | GCCATTGTCTTCACGTTTCC |
| Hgf | NM_017017 | TGATCCAAACATCCGAGTTG |  | CCATTGCCACGATAACAATCT |
| Hif1a | NM_024359 | CATGATGGCTCCCTTTTTCA |  | CATAGTAGGGGCACGGTCAC |
| Igf1 | NM_001082477 | CACACTGACATGCCCAAGAC |  | TCTCCTTTGCAGCTTCCTTT |
| Il10 | NM_012854 | CAGATTCCTTACTGCAGGACTTTA |  | CAAATGCTCCTTGATTTCTGG |
| Il1a | NM_017019 | AAATACTCAGCTCTTTGTGAGTGC |  | TGTGATGAGTTTTGGTGTTTCC |
| Il1r1 | NM_013123 | ATAGACAGACATAGAGGCTTTGGGG |  | CAGTGTAGCTTGGGATTTCACC |
| Il6 | NM_012589 | CACTTCACAAGTCGGAGGCT |  | TCTGACAGTGCATCATCGCT |
| Irf1 | NM_012591 | GAGCTGGGCCATTCACAC |  | CGATGTCTGGTAGGGAGTTCA |
| Itgad | NM_031691 | CCGGTGGAGTTGTGATCCTC |  | CGATGGGTTCCTCCACATCC |
| Itgam | NM_012711 | ATTGGGGCCCCTCATCACTA |  | CCACCGTGCTCTCCCCCTA |
| Klf6 | NM_031642 | TTGAAAGCACATCAGCGCAC |  | AGGTGGTCAGACCTGGAGAA |
| Kras | NM_031515 | GGAGGGCTTTCTTTGTGTATTTG |  | CCCATAACTCCTTGCTAACTCC |
| Lama1 | NM_001108237 | AGATTGGCTAAGACCGCACA |  | AGCTGCTTCAGCATTAGGGG |
| Lep | NM_013076 | TGTCTTCAACGGAGGAGAAAG |  | GTCCCGAGCACTTTGGATAA |
| Lrat | NM_022280 | TCCTGATAGTCAATTTGCTAGGC |  | CAACCAATCCAAACTTCCTTACA |
| Mapk1 | NM_053842 | GGCATGGTTTGTTCTGCTTATG |  | GTCTCCATGAGGTCCTGTACTA |
| Mmp13 | NM_133530 | TTGAGTTGGACTCACTGTTGGT |  | CTTCCTCAGACAAGTCATCATCA |
| Mmp14 | NM_031056 | ACAAAGATGCCCCCTCAAC |  | CCATAGGTGGGGTTTCTGG |
| Mmp2 | NM_031054 | CTGGTTGGAGGAGAACCAAG |  | TCCCATGGGGAACTGTTAAA |
| Mmp3 | NM_133523 | TGTGTTTCAGCTGACCCTGAT |  | TGCTAGAGTAAGGAAACCACTTCA |
| Mrc1 | NM_001106123 | CCCTGCTCCTGGCTTTTATCT |  | CTGAACGGAGATGGCGCTTA |
| Nfkb2 | NM_001008349 | CCACTGCATCTAGCCACAGA |  | ATTCACATTAGCATGGAGCTTG |
| Npy | NM_012614 | TGTGAAACCAGTCTGCCTGT |  | GAAATGGGTCGGAATCCAG |
| Pdgfa | NM_012801 | GGACAGGACGCGTAGAACAA |  | CGGGTTGCTCGAGGTCTTAG |
| Pdgfc | NM_031317 | GCAAGTTGCAGCTCTCCAG |  | TGGATGCTCCCATTACCAG |
| Pecam1 | NM_031591 | GCCTCACCAAGAGAACGGAA |  | ATTGGATGGCTTGGCCTGAA |
| Pklr | NM_012624 | GATACGAACCGGAGTCTTGC |  | ACCTGTGAGCCCTTCACAAT |
| Ppara | NM_013196 | CTGTCCCCAAAATGCCTGTG |  | ATTTTTCGCAAGGCCACGTT |
| Ptn | NM_017066 | GAAGCAGTTTGGAGCTGAGTG |  | GCTTGGGCTTGGTGAGTTTG |
| Rara | NM_031528 | CGGCTGAGTGACGAGAGC |  | GACTTCTACACTTTCGTACATCTTGC |
| Rbp1 | NM_012733 | CACGCTGAGCACTTTTCG |  | CCCAGCTCACTGTGGTCA |
| Rbp2 | NM_012640 | AGTGGGTCGAGGGAGACAA |  | TTGAACACTTGTCGACACACC |
| Rdh10 | NM_181478 | TTCAGAGGCTGCCGAATCAG |  | GTACATGAGACGAGGGGTGC |
| Serpine1 | NM_012620 | CACCCTTTGAAAAAGATGTGC |  | ATGAGCTCAGCGTCCAAAAT |
| Serpinh1 | NM_017173 | TTTTTGAGTTTTTCAAGGAATGG |  | TGTTTTGAAAGCAATAAAGGCTTC |
| Smad1 | NM_013130 | AGAAAGGGGCCATGGAAG |  | AGCGAGGAATGGTGACACA |
| Smad4 | NM_019275 | TCACAATGAGCTTGCATTCC |  | TCAAAGTAAGCAATGGAACACC |
| Smad7 | NM_030858 | CCCTGCTGTTGTTGCTGTC |  | ATGACCTCCGCACACCAT |
| Sosc3 | NM_053565 | AATCCAGCCCCAATGGTC |  | GGCCTGAGGAAGAAGCCTAT |
| Spp1 | NM_012881 | ATCGACAGTCAGGCGAGTTC |  | GCTGTGAAACTCGTGGCTCT |
| Stat3 | NM_012747 | GGGCCATCCTAAGCACAAA |  | AGACTGGATCTGGGTCTTGC |
| Tbp | NM_001004198 | CCCACCAGCAGTTCAGTAGC |  | CAATTCTGGGTTTGATCATTCTG |
| Tgfb1 | NM_021578 | GTCAACTGTGGAGCAACACG |  | GACAGCCACTCAGGCGTATC |
| Tgfb2 | NM_031131 | CCATACAGTCCCAGGTGCTC |  | GCAAGCGAAAGACCCTGAAC |
| Tgfbr2 | NM_031132 | AGAAGCCGCAGGAAGTCTG |  | GGCAAACGGTCTCCAGAGTA |
| Timp2 | NM_021989 | GTAGTGATCAGGGCCAAAGC |  | GATGGGGTTGCCATAGATGT |
| Tlr9 | NM_198131 | ACCTGTCTCGGAACAACCTG |  | AGATGGGAGAGGTTGACGAA |
| Tnf | NM_012675 | CCCTGGTACTAACTCCCAGAAA |  | TGTATGAGAGGGACGGAACC |
| Tnfr1 | NM_013091 | AATGAGTGCACCCCTTGC |  | CCTGGGGGTTTGTGACATT |
| Ubqln1 | NM_053747 | GAAGGAAGAGTTCGCTGTGC |  | TGAAACGTTTTGAGATTTCCTCT |
| Vcl | NM_001107248 | TACCAAGCGGGCACTTATTC |  | CCTTCACTGTGGACAGGATTT |
| Vegfa | NM_001110333 | AAAAACGAAAGCGCAAGAAA |  | TTTCTCCGCTCTGAACAAGG |
| Vim | NM_031140 | CGAGAAAAATTGCAGGAGGA |  | GAATGACTGCAGGGTGCTCT |
