# Supplementary material for: Cellular network modeling and single cell gene expression analysis reveals novel hepatic stellate cell phenotypes controlling liver regeneration dynamics
Source: BMC Syst Biol. 2018 Oct 3;12:86. doi: 10.1186/s12918-018-0605-7 (PMC6171157; doi:10.1186/s12918-018-0605-7)
Supplement: Supplementary file 5 — Zip file containing the raw mRNA expression data generated from single cells used in this manuscript. Data were manually inspected for reactions with CT values above those for water (below the limit of detection). These reactions were manually failed resulting in their removal from the data sets. Processed and unprocessed data are included. (ZIP 2160 kb) [file 12918_2018_605_MOESM5_ESM.zip › Tables S1 to S4.docx]

Table S1: Model parameter values for hepatocytes and physiological interpretations

| **Hepatocyte Parameters** | | |  |  |
| --- | --- | --- | --- | --- |
| **Parameter** | **Value** | **Physiological Interpretation** | **Effect of Parameter change on regeneration fraction (1/2 – 2x nominal)** | **Effect of Parameter change on regeneration fraction (1/10 – 10x nominal)** |
| M^hepatocyte^ | Species-specific 23.21 in rats | Metabolic demand | 0.89-0.0 | 0.9-0.0 |
| k_growth_ | Species-specific 8.29 x 10^-4^ in rats | Hepatocyte growth rate | 0.9-0.89 | 0-0.9 |
| k_IL6_^Hepatocyte^ | 0.3 | IL-6 production rate by hepatocytes | 0.87-0.92 | 0.84-0.98 |
| V_JAK_ | 6x10^4^ | JAK activation rate | 0.94-0.83 | 0.94-0.69 |
| K_m_^JAK^ | 10^4^ | JAK Michaelis constant | 0.83-0.94 | 0.69-0.94 |
| κ_JAK_ | 0.4 | JAK degradation rate | 0.94-0.82 | 0.98-0.0 |
| [STAT] | 2 | Concentration of monomeric STAT3 | 0.85-0.92 | 0.0-0.99 |
| V_STAT_ | 7.5x10^2^ | STAT3 activation rate | 0.88-0.9 | 0.8-0.91 |
| K_m_^STAT^ | 0.4 | STAT3 Michaelis constant | 0.91-0.87 | 0.95-0.79 |
| κ_STAT_ | 0.1 | STAT3 degradation rate | 0.89-0.89 | 0.89-0.89 |
| V_SOCS_ | 2.4x10^4^ | SOCS3 activation rate | 0.94-0.82 | 0.98-0.0 |
| K_m_^SOCS^ | 7x10^-4^ | SOCS3 Michaelis constant | 0.94-0.82 | 0.98-0.0 |
| κ_SOCS_ | 0.4 | SOCS3 degradation rate | 0.83-0.94 | 0.6-0.98 |
| K_I_^SOCS^ | 1.5x10^-2^ | SOCS3 inhibition of STAT3 signaling | 0.87-0.91 | 0.79-0.95 |
| V_IE_ | 2.5x10^2^ | Immediate early (IE) gene expression rate | 0.85-0.93 | 0.72-0.97 |
| K_m_^IE^ | 18 | IE gene Michaelis constant | 0.92-0.86 | 0.95-0.77 |
| κ_IE_ | 5 | IE gene degradation | 0.93-0.84 | 0.98-0.67 |
| k_Q→P_ | 7x10^-3^ | Hepatocyte priming rate | 0.84-0.93 | 0.67-0.98 |
| k_P→R_ | 4.4x10^-3^ | Hepatocyte replication transition rate | 0.85-0.93 | 0.75-0.98 |
| k_R→Q_ | 5.4x10^-2^ | Hepatocyte requiescence of replicating cells | 1.2-0.81 | 4.03-0.69 |
| k_prol_ | 2x10^-2^ | Hepatocyte proliferation rate | 0.8-1.24 | 0.7 – (>2.5) |
| k_req_ | 0.1 | Hepatocyte requiescence of primed cells | 0.92-0.86 | 0.96-0.77 |
| θ_req_ | 8 | Requiescence shape parameter | 0.93-0.86 | 0.96-0.86 |
| β_req_ | 3 | Requiescence scale parameter | 0.89-0.9 | 0.88-0.9 |
| k_ap_ | 0.1 | Hepatocyte apoptosis rate | 0.9-0.87 | 0.9-0.0 |
| θ_ap_ | 9x10^-3^ | Apoptosis shape parameter | 0.9-0.0 | 0.9-0.0 |
| β_ap_ | 4.5x10^-3^ | Apoptosis scale parameter | 0.92-0.0 | 0.92-0.0 |
| k_vas_ | 2x10^-2^ | Vascularization rate | 0.91-0.9 | 0.93-0.9 |

Table S2: Model parameter values for Kupffer cells and physiological interpretations

| **Kupffer cell parameters** | | |  |  |
| --- | --- | --- | --- | --- |
| **Parameter** | **Value** | **Physiological Interpretation** | **Effect of Parameter change on regeneration fraction (1/2–2x nominal)** | **Effect of Parameter change on regeneration fraction (1/10–10x nominal)** |
| M^KC^ | Species-specific 23.21 in rats | Metabolic demand | 0.83-0.87 | 0.72-0.7 |
| HL | 2 | Hypoxia load | 0.89-0.89 | 0.89-0.89 |
| k_TNF_ | 16 | TNFa production rate | 0.83-0.97 | 0.74-1.02 |
| κ_TNF_ | 2 | TNFa degradation rate | 0.97-0.83 | 1.02-0.74 |
| k_IL6_^KC^ | 180 | IL-6 production rate by Kupffer cells | 0.77-0.97 | 0.54-1.0 |
| κ_IL6_ | 0.9 | IL-6 degradation rate | 0.91-0.87 | 0.92-0.67 |
| k_IL10_ | 20 | IL-10 production rate | 0.91-0.87 | 0.93-0.83 |
| κ_IL10_ | 0.9 | IL-10 degradation rate | 0.87-0.91 | 0.83-0.94 |
| K_I_^IL10^ | 3 | IL-10 inhibition of TNFa production | 0.94-0.79 | 0.97-0.62 |
| k_TGF_^KC^ | 30 | TGF-β production rate by Kupffer cells | 0.89-0.89 | 0.89-0.89 |
| k_PDGF_ | 15 | PDGF production rate | 0.89-0.89 | 0.89-0.89 |
| κ_PDGF_ | 0.9 | PDGF degradation rate | 0.89-0.89 | 0.89-0.89 |
| k_Q→A_^KC^ | 7x10^-3^ | Kupffer cell activation rate | 0.82-0.96 | 0.71-1.0 |
| k_A→R_^KC^ | 2.2x10^-2^ | Kupffer cell replication transition rate | 0.89-0.9 | 0.88-0.95 |
| k_R→Q_^KC^ | 5.4x10^-2^ | Kupffer cell requiescence of replicating cells | 0.92-0.89 | 1.04-0.88 |
| k_prol_^KC^ | 2x10^-2^ | Kupffer cell proliferation rate | 0.89-0.93 | 0.88-1.04 |
| k_req_^KC^ | 0.3 | Kupffer cell requiescence of primed cells | 0.96-0.82 | 1.01-0.72 |
| θ_req_^KC^ | 8 | Kupffer cell requiescence shape parameter | 0.95-0.88 | 1.12-0.88 |
| β_req_^KC^ | 3 | Kupffer cell requiescence scale parameter | 0.89-0.91 | 0.89-0.94 |
| k_ap_^KC^ | 0.1 | Kupffer cell apoptosis rate | 0.89-0.89 | 0.89-0.87 |
| θ_ap_^KC^ | 9x10^-3^ | Kupffer cell apoptosis shape parameter | 0.89-0.82 | 0.9-0.69 |
| β_ap_^KC^ | 4.5x10^-3^ | Kupffer cell apoptosis scale parameter | 0.9-0.87 | 0.9-0.7 |

Table S3: Model parameter values for hepatic stellate cells and physiological interpretations

| **Hepatic stellate cell parameters** | | |  |  |
| --- | --- | --- | --- | --- |
| **Parameter** | **Value** | **Physiological Interpretation** | **Effect of Parameter change on regeneration fraction (1/2–2x nominal)** | **Effect of Parameter change on regeneration fraction (1/10–10x nominal)** |
| M^HSC^ | Species-specific 23.21 in rats | Metabolic demand | 0.89-0.87 | 0.89-0.79 |
| k_HGF_ | 25 | HGF production rate | 0.81-0.95 | 0.69-0.99 |
| κ_HGF_ | 0.23 | HGF degradation rate | 0.91-0.86 | 0.92-0.75 |
| k_up_ | 0.6 | HGF uptake by ECM | 0.94-0.83 | 0.97-0.71 |
| k_TGF_^HSC^ | 8 | TGF-β production rate by hepatic stellate cells | 0.91-0.85 | 0.92-0.68 |
| κ_TGF_ | 0.9 | TGF-β degradation rate | 0.76-0.95 | 0.63-0.97 |
| K_I_^TGF^ | 5 | TGF-β inhibition of HGF production | 0.93-0.82 | 0.95-0.61 |
| k_ECM_ | 100 | ECM production rate | 0.82-0.93 | 0.67-0.96 |
| k_degrad_ | 7 | ECM degradation rate by MMPs | 0.89-0.89 | 0.9-0.88 |
| κ_ECM_ | 3 | Constitutive ECM degradation rate | 0.81-0.95 | 0.66-0.99 |
| k_Q→PR_ | 7x10^-2^ | State transition rate: Quiescent to Pro-regenerative | 0.94-0.79 | 0.96-0.62 |
| k_Q→AR_ | 3x10^-2^ | State transition rate: Quiescent to Anti-regenerative | 0.89-0.89 | 0.89-0.89 |
| k_PR→PRR_ | 4.4x10^-3^ | State transition rate: Pro-regenerative to Replicating | 0.89-0.89 | 0.89-0.89 |
| k_AR→ARR_ | 4.4x10^-3^ | State transition rate: Anti-regenerative to Replicating | 0.89-0.89 | 0.89-0.89 |
| k_PRR→PR_ | 5.4x10^-2^ | State transition rate: Replicating to Pro-regenerative | 0.89-0.89 | 0.89-0.89 |
| k_ARR→AR_ | 5.4x10^-2^ | State transition rate: Replicating to Anti-regenerative | 0.89-0.89 | 0.89-0.9 |
| k_prol_ | 8.5x10^-3^ | Hepatic stellate cell proliferation rate | 0.89-0.88 | 0.88-0.84 |
| k_req_ | 0.2 | Requiescence rate of pro- and anti-regenerative cells | 0.89-0.89 | 0.89-0.89 |
| θ_req_^HSC^ | 8 | Hepatic stellate cell requiescence shape parameter | 0.89-0.89 | 0.89-0.89 |
| β_req_^HSC^ | 3 | Hepatic stellate cell requiescence scale parameter | 0.89-0.89 | 0.89-0.89 |
| k_ap_^HSC^ | 0.1 | Hepatic stellate cell apoptosis rate | 0.89-0.88 | 0.89-0.78 |
| θ_ap_^HSC^ | 9x10^-3^ | Hepatic stellate cell apoptosis shape parameter | 0.89-0.89 | 0.89-0.82 |
| β_ap_^HSC^ | 4.5x10^-3^ | Hepatic stellate cell apoptosis scale parameter | 0.85-0.94 | 0.81-0.99 |

Table S4: Primer sequences used in the single-cell gene expression experiments

| **Gene** | **RefSeq ID** | **Forward Sequence** |  | **Reverse Sequence** |
| --- | --- | --- | --- | --- |
| Actb | NM_031144 | AAGGCCAACCGTGAAAAGAT |  | ACCAGAGGCATACAGGGACA |
| Adamts1 | NM_024400 | GGACAGGTGCAAGCTTACCT |  | CACAGCCAGCTTTCACACAC |
| Adamts13 | XM_006233879 | ACCCTCTCAGGAGGCTAAAT |  | GTGACTGGGATTCTGGTTAGTG |
| Adh1a | NM_019286 | GATGCCGACTTGGACATTGC |  | TGGCTCGCTCAACACTCTTT |
| Alb | NM_134326 | TGGCACAATGAAGTGGGTAA |  | GGGCGATCTCACTCTTGTGT |
| Aldh1a1 | NM_022407 | GCCATCACTGTGTCTTCTGC |  | CATCTTGAATCCACCGAAGG |
| Aldh2 | NM_032416 | TTACCTGTCCCAAGCTCTGC |  | GCACGCCACTTTACGAGTTC |
| Aldh7a1 | NM_001271105 | GGAATCATCACTGCCTTCAAC |  | AGTTGTTGGTGCTCCTTTCC |
| Ang1 | NM_001006992 | CGTCCTCTGTTGTCGGTTTT |  | CGTGTACCTGGGGTCGTC |
| Apoa4 | NM_012737 | AGCCCCTGGGGGATAAGT |  | CCAGCTGCTGCCTGAACT |
| Arg1 | NM_017134 | GGTAGAGAAAGGTCCCGCAG |  | CAGACCGTGGGTTCTTCACA |
| Bambi | NM_139082 | AGCAGAAACCTCATCACTAAGG |  | GCTGTAGTGCAAACGAGAGA |
| Bmp6 | NM_013107 | CAGCAACAATCGCAACAGAC |  | GGGAGTTGTAGAGATCCAGCA |
| Casp3 | NM_012922 | TCTACCGCACCCGGTTACTA |  | TGACTGGATGAACCATGACC |
| Ccl3 | NM_013025 | CGCCATATGGAGCTGACAC |  | GTGGAATTTGCCGTCCATAG |
| Ccnd1 | NM_171992 | TGTGATATGTACCAGCCACAGG |  | CGAACAGACGACGGCATACT |
| Cdkn1a | NM_080782 | GCGCCCTCCGTTTCTTACTT |  | TCGCAGACCTCTAGCATCCA |
| Ch25h | NM_001025415 | CTGCACTGGAACAGGGCTAA |  | ACTGCCCAGCAGGAACAAAT |
| Clcn3 | NM_053363 | GACTGTCTCTCTGGTGGTTATTG |  | GCCAGGGTTGTATGAGTGAA |
| Col14a1 | NM_001130548 | CAGGCCAGAGGGGATTTC |  | AATGCCAATTGGTCCAGGT |
| Col3a1 | NM_032085 | ATGTGGGACCTGGTTTCTTC |  | CAGTCTAGTGGCTCATCATCAC |
| Col4A1 | NM_001135009 | CCAGCGGTGGTTATGACTTC |  | GGCCACCATCTTGAGACTTC |
| Col4A2 | XM_001076134 | CTGTCAGCAAATGGGCACT |  | TTAGGAGGTGGGTGTTAGCAG |
| Csf1 | NM_023981 | CTGACTCTGGTAGGGAAGGATA |  | GAGACCAAGGAGCAAGTAAGAG |
| Csf2 | NM_053852 | CTAATGAGTTCTCCATCCAGAGG |  | CCCGTAGACCCTGCTTGTAT |
| Csf3 | NM_017104 | GGTTTTCCTGACCCCGTAGG |  | TAGGCCAGCAAGCGCTAAAA |
| Csrp2 | NM_177425 | ACATGGACCGTGGTGAGAG |  | GTAGGCCTGTGAGGTTGAGC |
| Cxcl1 | NM_030845 | ACTCAAGAATGGTCGCGAGG |  | ACGCCATCGGTGCAATCTAT |
| Cxcl12 | NM_001033882 | GGCCTCTGGGCACAGTTA |  | TGGTGGAAGGTTGCTACTCC |
| Cyp1a1 | NM_012540 | CTCCCTGGGGTCCTAGAGAACA |  | CTCTGTGGCTGATGTGAAGGC |
| Cyp1a2 | NM_012541 | GGAACACTATCAAGACTTCAACAAGA |  | AATCCAGCTCCAAAGATGTCA |
| Cyp27b1 | NM_053763 | GGCTCCTATGCCCACCTC |  | CACAGCCTTTAGCAGGGGTA |
| Cyp2b1 | NM_001134844 | CGGACCTTTTCCCTCCTAAG |  | GGAACCCAGAGAAGAACTCAAA |
| Cyp2e1 | NM_031543 | CTGACTGTCTCCTCATAGAGATGG |  | TCACAGAAACATTTTCCATTGTGT |
| Dcn | NM_024129 | CGGTGGCAAATACCCGGATTA |  | TCTGCTCAAATGGTCCAGCC |
| Ecm1 | NM_053882 | TGACCCGTGACCAGTTCTTAC |  | GGTGCTGCATAGCCTACTTC |
| Erlin2 | NM_001106088 | CCCAGAAACAGAAGGTGGTG |  | GCAACCTGTGCCACTTTTTC |
| Fap | NM_138850 | GAAGAGGAAATGCTTGCTACAAA |  | TGGTATGTCCGAATCATTAAATTC |
| Fn1 | NM_019143 | CAGCCCCTGATTGGAGTC |  | TGGGTGACACCTGAGTGAAC |
| Fos | NM_022197 | GGGACAGCCTTTCCTACTACC |  | GATCTGCGCAAAAGTCCTGT |
| Gapdh | NM_017008 | TGGCCTCCAAGGAGTAAGAA |  | GGCCTCTCTCTTGCTCTCAG |
| Gfap | NM_017009 | AAGATCCCGAGGCAAAGAAT |  | TCGTCAGGGTTCTTCCAGAT |
| Got1 | NM_012571 | ACGAATCACCTGGTCCAATC |  | GCCATTGTCTTCACGTTTCC |
| Hgf | NM_017017 | TGATCCAAACATCCGAGTTG |  | CCATTGCCACGATAACAATCT |
| Hif1a | NM_024359 | CATGATGGCTCCCTTTTTCA |  | CATAGTAGGGGCACGGTCAC |
| Igf1 | NM_001082477 | CACACTGACATGCCCAAGAC |  | TCTCCTTTGCAGCTTCCTTT |
| Il10 | NM_012854 | CAGATTCCTTACTGCAGGACTTTA |  | CAAATGCTCCTTGATTTCTGG |
| Il1a | NM_017019 | AAATACTCAGCTCTTTGTGAGTGC |  | TGTGATGAGTTTTGGTGTTTCC |
| Il1r1 | NM_013123 | ATAGACAGACATAGAGGCTTTGGGG |  | CAGTGTAGCTTGGGATTTCACC |
| Il6 | NM_012589 | CACTTCACAAGTCGGAGGCT |  | TCTGACAGTGCATCATCGCT |
| Irf1 | NM_012591 | GAGCTGGGCCATTCACAC |  | CGATGTCTGGTAGGGAGTTCA |
| Itgad | NM_031691 | CCGGTGGAGTTGTGATCCTC |  | CGATGGGTTCCTCCACATCC |
| Itgam | NM_012711 | ATTGGGGCCCCTCATCACTA |  | CCACCGTGCTCTCCCCCTA |
| Klf6 | NM_031642 | TTGAAAGCACATCAGCGCAC |  | AGGTGGTCAGACCTGGAGAA |
| Kras | NM_031515 | GGAGGGCTTTCTTTGTGTATTTG |  | CCCATAACTCCTTGCTAACTCC |
| Lama1 | NM_001108237 | AGATTGGCTAAGACCGCACA |  | AGCTGCTTCAGCATTAGGGG |
| Lep | NM_013076 | TGTCTTCAACGGAGGAGAAAG |  | GTCCCGAGCACTTTGGATAA |
| Lrat | NM_022280 | TCCTGATAGTCAATTTGCTAGGC |  | CAACCAATCCAAACTTCCTTACA |
| Mapk1 | NM_053842 | GGCATGGTTTGTTCTGCTTATG |  | GTCTCCATGAGGTCCTGTACTA |
| Mmp13 | NM_133530 | TTGAGTTGGACTCACTGTTGGT |  | CTTCCTCAGACAAGTCATCATCA |
| Mmp14 | NM_031056 | ACAAAGATGCCCCCTCAAC |  | CCATAGGTGGGGTTTCTGG |
| Mmp2 | NM_031054 | CTGGTTGGAGGAGAACCAAG |  | TCCCATGGGGAACTGTTAAA |
| Mmp3 | NM_133523 | TGTGTTTCAGCTGACCCTGAT |  | TGCTAGAGTAAGGAAACCACTTCA |
| Mrc1 | NM_001106123 | CCCTGCTCCTGGCTTTTATCT |  | CTGAACGGAGATGGCGCTTA |
| Nfkb2 | NM_001008349 | CCACTGCATCTAGCCACAGA |  | ATTCACATTAGCATGGAGCTTG |
| Npy | NM_012614 | TGTGAAACCAGTCTGCCTGT |  | GAAATGGGTCGGAATCCAG |
| Pdgfa | NM_012801 | GGACAGGACGCGTAGAACAA |  | CGGGTTGCTCGAGGTCTTAG |
| Pdgfc | NM_031317 | GCAAGTTGCAGCTCTCCAG |  | TGGATGCTCCCATTACCAG |
| Pecam1 | NM_031591 | GCCTCACCAAGAGAACGGAA |  | ATTGGATGGCTTGGCCTGAA |
| Pklr | NM_012624 | GATACGAACCGGAGTCTTGC |  | ACCTGTGAGCCCTTCACAAT |
| Ppara | NM_013196 | CTGTCCCCAAAATGCCTGTG |  | ATTTTTCGCAAGGCCACGTT |
| Ptn | NM_017066 | GAAGCAGTTTGGAGCTGAGTG |  | GCTTGGGCTTGGTGAGTTTG |
| Rara | NM_031528 | CGGCTGAGTGACGAGAGC |  | GACTTCTACACTTTCGTACATCTTGC |
| Rbp1 | NM_012733 | CACGCTGAGCACTTTTCG |  | CCCAGCTCACTGTGGTCA |
| Rbp2 | NM_012640 | AGTGGGTCGAGGGAGACAA |  | TTGAACACTTGTCGACACACC |
| Rdh10 | NM_181478 | TTCAGAGGCTGCCGAATCAG |  | GTACATGAGACGAGGGGTGC |
| Serpine1 | NM_012620 | CACCCTTTGAAAAAGATGTGC |  | ATGAGCTCAGCGTCCAAAAT |
| Serpinh1 | NM_017173 | TTTTTGAGTTTTTCAAGGAATGG |  | TGTTTTGAAAGCAATAAAGGCTTC |
| Smad1 | NM_013130 | AGAAAGGGGCCATGGAAG |  | AGCGAGGAATGGTGACACA |
| Smad4 | NM_019275 | TCACAATGAGCTTGCATTCC |  | TCAAAGTAAGCAATGGAACACC |
| Smad7 | NM_030858 | CCCTGCTGTTGTTGCTGTC |  | ATGACCTCCGCACACCAT |
| Sosc3 | NM_053565 | AATCCAGCCCCAATGGTC |  | GGCCTGAGGAAGAAGCCTAT |
| Spp1 | NM_012881 | ATCGACAGTCAGGCGAGTTC |  | GCTGTGAAACTCGTGGCTCT |
| Stat3 | NM_012747 | GGGCCATCCTAAGCACAAA |  | AGACTGGATCTGGGTCTTGC |
| Tbp | NM_001004198 | CCCACCAGCAGTTCAGTAGC |  | CAATTCTGGGTTTGATCATTCTG |
| Tgfb1 | NM_021578 | GTCAACTGTGGAGCAACACG |  | GACAGCCACTCAGGCGTATC |
| Tgfb2 | NM_031131 | CCATACAGTCCCAGGTGCTC |  | GCAAGCGAAAGACCCTGAAC |
| Tgfbr2 | NM_031132 | AGAAGCCGCAGGAAGTCTG |  | GGCAAACGGTCTCCAGAGTA |
| Timp2 | NM_021989 | GTAGTGATCAGGGCCAAAGC |  | GATGGGGTTGCCATAGATGT |
| Tlr9 | NM_198131 | ACCTGTCTCGGAACAACCTG |  | AGATGGGAGAGGTTGACGAA |
| Tnf | NM_012675 | CCCTGGTACTAACTCCCAGAAA |  | TGTATGAGAGGGACGGAACC |
| Tnfr1 | NM_013091 | AATGAGTGCACCCCTTGC |  | CCTGGGGGTTTGTGACATT |
| Ubqln1 | NM_053747 | GAAGGAAGAGTTCGCTGTGC |  | TGAAACGTTTTGAGATTTCCTCT |
| Vcl | NM_001107248 | TACCAAGCGGGCACTTATTC |  | CCTTCACTGTGGACAGGATTT |
| Vegfa | NM_001110333 | AAAAACGAAAGCGCAAGAAA |  | TTTCTCCGCTCTGAACAAGG |
| Vim | NM_031140 | CGAGAAAAATTGCAGGAGGA |  | GAATGACTGCAGGGTGCTCT |
